# Enzyme modification using mutation site prediction method for enhancing the regioselectivity of substrate reaction sites

**DOI:** 10.1038/s41598-021-98433-7

**Published:** 2021-10-04

**Authors:** Jinzen Ikebe, Munenori Suzuki, Aya Komori, Kaito Kobayashi, Tomoshi Kameda

**Affiliations:** 1 Artificial Intelligence Research Center, National Institute of Advanced Industrial Science and Technology (AIST), 2-4-7 Aomi, Koto-ku, Tokyo, 135-0064 Japan; 2 KNC Bio-Research Center, KNC Laboratories Co., Ltd., 1-1-1 Murotani, Nishi-ku, Kobe, Hyogo 651-2241 Japan

**Keywords:** Biocatalysis, Computational biophysics, Protein design, Biocatalysis, Computational chemistry, Protein design

## Abstract

Enzymes with low regioselectivity of substrate reaction sites may produce multiple products from a single substrate. When a target product is produced industrially using these enzymes, the production of non-target products (byproducts) causes adverse effects such as increased processing costs for purification and the amount of raw material. Thus it is required the development of modified enzymes to reduce the amount of byproducts’ production. In this paper, we report a method called mutation site prediction for enhancing the regioselectivity of substrate reaction sites (MSPER). MSPER takes conformational data for docking poses of an enzyme and a substrate as input and automatically generates a ranked list of mutation sites to destabilize docking poses for byproducts while maintaining those for target products in silico. We applied MSPER to the enzyme cytochrome P450 CYP102A1 (BM3) and the two substrates to enhance the regioselectivity for four target products with different reaction sites. The 13 of the total 14 top-ranked mutation sites predicted by MSPER for the four target products succeeded in selectively enhancing the regioselectivity up to 6.4-fold. The results indicate that MSPER can distinguish differences of substrate structures and the reaction sites, and can accurately predict mutation sites to enhance regioselectivity without selection by directed evolution screening.

## Introduction

Enzymes are well known to play a major role in brewing and fermentation. Various enzymatic reactions have been extensively used in industrial processing, including in the laundry, food, fiber, tannery, and pharmaceutical and chemical industries, due to the application of enzyme modification technology^1^. Since ecological awareness and the use of practices such as green sustainable chemistry have increased, attention has been given to the development of enzymatic reactions rather than to conventional chemical methods^2^. Early in enzyme modification research, enzymes were chemically modified to improve aminolysis (rearrangement reaction to amino groups), such as by thiolation^3^. Since then, the development of genetic engineering has made it possible to modify enzymes by changing amino acids through gene recombination^4^. Activity, stability, and substrate selectivity are major considerations when screening or modifying enzymes for enzymatic reactions for industrial use. Another important consideration is the regioselectivity of substrate reaction sites. Enzymes with low regioselectivity produce multiple products from a single substrate. When these enzymes are used to produce a target product industrially, the processing cost of extracting the target product from the mixture with non-target products (byproducts) is incurred. To control the cost and produce the target product efficiently, it is required the development of modified enzymes to reduce the amount of byproducts’ production while maintaining the target product’s production.

Directed evolution^5–9^ widely used as an enzymatic modification technique mimics the process of natural selection and identifies promising enzyme mutants by repeating two processes: the creation of numerous mutants randomly mutated and the selection of valid mutants by screening. For efficient screening, simple and fast methods for evaluating the enzymatic function of mutants are required. High-throughput bioassays that can be used to evaluate a target compound’s optical properties such as absorption spectroscopy and spectrophotometric and fluorescence detection methods are used extensively for selection improvement. However, these methods cannot easily distinguish position isomers with similar physical properties. To evaluate the regioselectivity of mutants, relatively laborious bioassays, such as mass spectrometry (MS), gas chromatography MS (GC–MS), and liquid chromatography MS (LC–MS), are required to investigate the chemical structures of the target compound and byproducts in detail. Therefore, enhancing enzyme regioselectivity by directed evolution is time-consuming and labor-intensive.

For the regioselectivity enhancement that requires laborious bioassay, it is necessary to reduce the number of mutants to be evaluated as much as possible. Rational design^10–18^ identifies promising mutations based on detailed information about enzyme conformation, evolutionary information, and the catalytic mechanism. Whereas directed evolution introduces mutations randomly, rational design reduces the number of mutants to be evaluated by targeting only “hot spots”, which are residues that may alter functions of enzymes, located in the active site or access tunnels to the active site. In terms of rational design for enhancing regioselectivity, it is interesting that Seifert et al*.* have developed an effective mutant that selectively generates the target product from a less regioselective cytochrome P450 CYP102A1 (BM3)^19^. They proposed amino acid substitutions to narrow down the space of the enzyme active site and controlled the regioselectivity so that only the smallest methyl group in the substrate could access the active site. Though their strategy using the effect of steric hindrance is very effective for the reaction of exocyclic or terminal structures, it would be constructed laboriously for the regioselective reaction of endocyclic or internal structures due to demand the accurate control of docking conformation. In contrast, we would like to develop a method that allows us to freely control reaction sites of substrates and automatically predict mutation sites to enhance regioselectivity.

Here, we report a method called mutation site prediction for Enhancing the Regioselectivity of substrate reaction sites (MSPER). Enzymes with low regioselectivity form multiple complex enzyme–substrate structures (docking poses) for each reaction product. MSPER takes these docking pose data as input and automatically generates a ranked list of residue substitution sites to destabilize docking poses for byproducts while maintaining those for the target product. We selected CYP102A1 (BM3), a cytochrome P450 family member for which the crystal structure of the N-terminal P450 heme domain has been reported, as a model enzyme for regioselectivity control of the enzymatic reaction^20^. CYP102A1, which catalyzes γ-2 hydroxylation of saturated fatty acids and hydroxylation and epoxidation of unsaturated fatty acids, was isolated from *Bacillus megaterium* in 1986^21^. Various substrates that are catalyzed by CYP102A1 and its various mutant oxidized compounds, such as monoterpenoids, sesquiterpenoids, steroids, and alkaloids, have since been reported^22^. In particular, monoterpenoids have been extensively used as flavors, fragrances, antiseptic agents, and pharmaceutical intermediates. Since the chemical structures of monoterpenoids affect their flavor and fragrance notes, high regioselectivity is an important issue related to enzymatic reactions used in industrial manufacturing. We focused on oxidized monoterpenoids, such as carveol, isopiperitenol, thymol, and carvacrol, which are used for flavor and intermediate applications, and tried to improve their production rates.

In the current study, we generated docking poses of CYP102A1 and the two substrates, (*S*)-(−)-limonene and *p*-cymene, using molecular dynamics (MD) simulations and applied MSPER on them to predict mutation sites to improve production rates of four target products (*trans*-carveol and *cis*-isopiperitenol from (*S*)-(−)-limonene, and carvacrol and thymol from *p*-cymene), which are internal ring hydroxylation products rather than terminal ones. MSPER significantly reduced the number of mutants tested by reducing the number of candidate substitution residues from the 455 residues that make up the enzyme to just a few residues. Mutation sites top-ranked by MSPER selectively increased the production rates at 13 out of 14 sites in total for the four target products with different substrate reaction sites up to 6.4-fold. On the other hand, the substitution of the lowest-ranked residues caused a significant decrease in the production rates. These results indicate that MSPER can distinguish differences of substrate structures and the reaction sites, and can accurately predict mutant sites to enhance regioselectivity.

## Results

We describe the definition of the docking poses used in this work and the details of MSPER below. When an enzyme catalyzes target products or byproducts, it must form the appropriate docking poses (target product docking poses or byproduct poses) in which the enzyme active site contacts the corresponding substrate reaction sites. It is believed that residues in contact with the substrate in a docking pose (contact residues) are important for stabilizing the pose. To enhance the substrate reaction site’s regioselectivity, contact residues in target product docking poses must not be substituted to maintain target product production. Contact residues in byproduct docking poses should actively be substituted to decrease the production of byproducts. Our prediction method, MSPER, identifies candidate substitution residues that maintain the target product docking poses and destabilize the byproduct docking poses.

**Figure 1:**
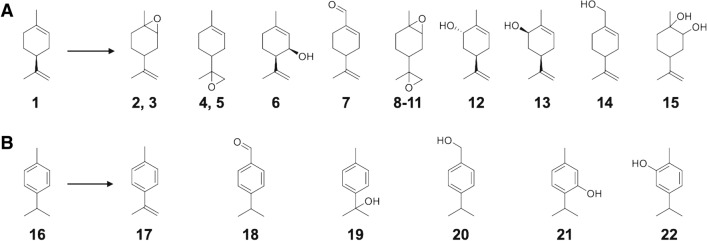
Various oxidized compounds detected from reaction products converted by wild-type CYP102A1. (**A**) (*S*)-(−)-Limonene (**1**) was converted to 14 oxidized compounds, i.e., limonene-1,2-epoxide and its isomer (**2**, **3**), limonene-8,9-epoxide and its isomer (**4**, **5**), *cis*-isopiperitenol (**6**), (−)-perillaldehyde (**7**), limonene diepoxide and its isomers (**8**–**11**), *trans*-carveol (**12**), *cis*-carveol (**13**), perillyl alcohol (**14**) and limonene-1,2-diol (**15**). (**B**) The enzyme converted *p*-cymene (**16**) to six compounds, i.e., *p*-cymenene (**17**), cuminaldehyde (**18**), *p*,*α*,*α*-trimethylbenzyl alcohol (**19**), 4-isopropylbenzyl alcohol (**20**), thymol (**21**), and carvacrol (**22**).

**Figure 2:**
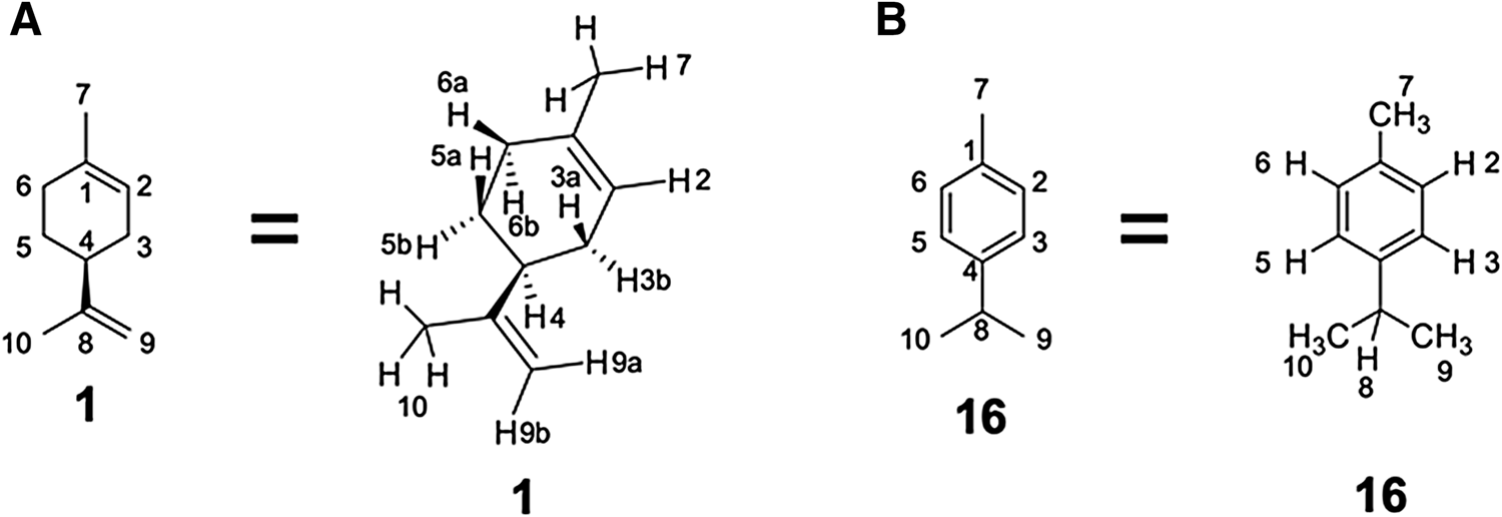
Chemical structures of substrates, such as (**A**) (*S*)-(−)-limonene (**1**) and (B) *p*-cymene (**16**), with a number to each carbon and hydrogen atom. All 16 and 14 hydrogen atoms in (S)-(-)-limonene and p-cymene (**16**), respectively, were used to define the docking pose groups.

Enzymes with low regioselectivity of substrate reaction sites such as a cytochrome P450 CYP102A1 can assume multiple docking poses corresponding to each product (Fig. 1). We defined the docking poses as follows. For each conformation, we calculated the distances between the active site of the enzyme and substrate reaction sites (in this study, the oxygen atom covalently bonded to the Fe atom in heme and the hydrogen atoms located at the substrate reaction sites [16 and 14 hydrogen atoms in (*S*)-(−)-limonene (**1**) and *p*-cymene (**16**), respectively, as shown in Fig. 2)]. When the minimum distance between these sites was less than the sum of the radii of the two atoms and the tolerance (oxygen vdW radius 1.48 Å + hydrogen’s radius 1.00 Å + tolerance 1.00 Å = 3.48 Å in this study), the conformation was considered a docking pose in which the closest reaction site contacts the active site. For example, if the closest hydrogen was the H2 atom, the docking pose was called the H2 docking pose and belonged to the H2 docking pose group. Note that a docking pose could not belong to multiple docking pose groups simultaneously. Thus, the simulated ensemble was divided into two docking pose groups, i.e., the target product docking pose group and the sum of the other docking pose groups (the byproduct docking group), and the substrate was not docked in the other conformations.

To identify candidate substitution residues, we obtained enzyme–substrate contacts to stabilize the docking poses. We calculated the distances between an atom (a heavy atom, not hydrogen) in the enzyme (an enzyme heavy atom) and heavy atoms in the substrate (substrate heavy atoms). When the minimum distance was less than the sum of vdW radii of the enzyme heavy atom and the closest substrate atom + tolerance of 1.00 Å, the enzyme heavy atom was considered in contact with the substrate. For each docking pose group, the average contact rate of an enzyme heavy atom (*C*) was calculated as follows:

1 $$\begin{array}{*{20}c} {C = \mathop \sum \limits_{i = 1}^{N} cw_{i} /\mathop \sum \limits_{i = 1}^{N} w_{i} } \\ \end{array} ,$$

where *N* is the total number of conformations in the docking pose group, *c* is the contact condition (when the enzyme heavy atom is in contact with the substrate, *c* = 1, otherwise *c* = 0). Though *w*_*i*_ is a weight coefficient for the *i*-th conformation (see the reweighting factor of ALSD simulation in “Methods” section for the details), it is 1 if the docking pose is from X-ray crystal conformation, an NMR model, docking simulation, or a conventional MD simulation. To identify atoms with low contact rates in the target product docking pose and high contact rates in the byproduct docking pose, we calculated the difference in contact rate (*D*) as follows:

2 $$\begin{array}{*{20}c} {D = C_{{{\text{bp}}}} - C_{{{\text{tp}}}} } \\ \end{array} ,$$

where C_bp_ and C_tp_ are the contact rates of the byproduct docking pose group and the target product group, respectively. Finally, the substitution candidate residue score (*S*_scr_) for each residue was calculated as

3 $$\begin{array}{*{20}c} {S_{{{\text{scr}}}} = \mathop \sum \limits_{i = 1}^{N} D_{i} } \\ \end{array} ,$$

where *N* is the number of heavy atoms in the residue, and *D*_*i*_ is the difference in contact rate of the *i*-th heavy atom in the residue. In the current study, two simulations with different initial conformations were performed to check the convergence of the simulation results, and amino acid residues were ranked according to *S*_scr_ for each simulation. Note that residues corresponding to the active site (CYS 400 covalently bonded to heme in this study) were removed from the ranked list. The residues with the highest average rank from the two simulations were considered candidate substitution residues.

Wild-type CYP102A1 converted (*S*)-(−)-limonene (**1**) to 14 oxidized compounds (Fig. 1A and Supplementary Figs. 1 and 2). Epoxide compounds, i.e., limonene-1,2-epoxide (**2** and **3**), limonene-8,9-epoxide (**4** and **5**), limonene diepoxide (**8**, **9**, **10**, and **11**), and these diastereomers comprised approximately 80% of the total reaction products. On the other hand, the composition of the target alcohols was as follows: 5.4% were composed of *cis*-isopiperitenol (**6**) and 9.8% were composed of *trans*-carveol (**12**) (Supplementary Table 1). Additionally, the wild-type enzyme catalyzed the conversion of 6 reaction products from *p*-cymene (**16**) (Fig. 1B and Supplementary Figs. 3 and 4). *p*,α,α-Trimethylbenzyl alcohol (**19**) was the main component, comprising 64.5% of the total reaction compounds, while thymol (**21**) and carvacrol (**22**), as target alcohols, comprised 4.7% and 13.5% of these compounds, respectively (Supplementary Table 14). See Fig. 2A,B for the names of the hydrogen atoms located at the substrate reaction sites (7 and 14 hydrogen atoms for (*S*)-(−)-limonene (**1**) and *p*-cymene (**16**), respectively.

**Figure 3:**
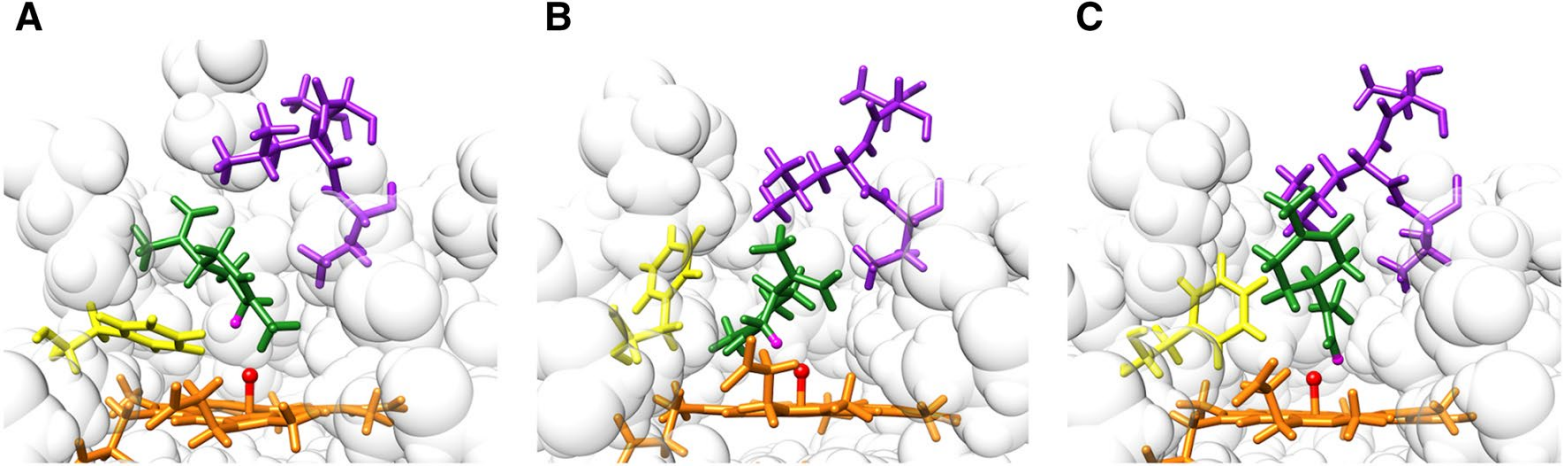
Conformational examples of docking poses in which H6b [corresponding to *trans*-carveol (**12**), (**A**)], H3a [corresponding to *cis*-isopiperitenol (**6**), (**B**)], and H9b [the largest component of the byproduct docking pose group, (**C**)] of (*S*)-(−)-limonene contact the O atom bound to the Fe atom in heme. A view of the active site from the pocket entrance side. The heme and (*S*)-(−)-limonene are represented by orange and green sticks, respectively. The active site, heme oxygen is a red ball. The modification sites corresponding to H6b, H3a, and H9b are taken as magenta spheres. The orientation of the side chain ring of F87 (yellow stick) and the β-turn of T436-T438 (purple stick) changed depending on the modification sites on the substrate.

**Figure 4:**
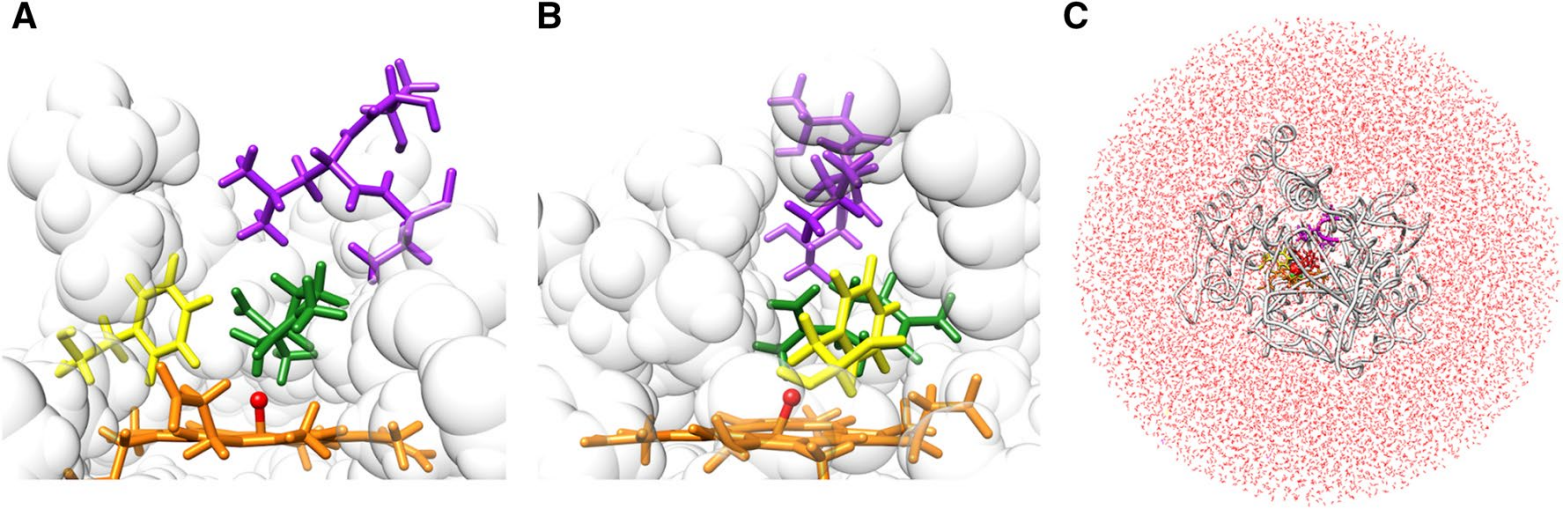
(**A**) A tentative complex conformation of the (*S*)-(−)-limonene docked into the BMP X-ray crystal conformation with the resolution of 1.65 Å (PDB ID: 1BU7) using ZDOCK software. The coordinates of the missing hydrogen atoms in the BMP crystal structure were automatically generated by an MD simulation program PRESTO ver. 3. See the legend in Fig. 3 for the coloring and representation in the figure. This conformation was used as a pre-initial conformation to generate the initial different conformations for 72 MD simulation runs. A view of the active site from the pocket entrance side. (**B**) A view of the active site from the F87 side. (**C**) The whole simulation system with water molecules and ions.

To enhance the regioselectivity of (*S*)-(−)-limonene oxidization, we generated docking poses of the N-terminal P450 heme domain (BMP) of cytochrome P450 BM3 (CYP102A1) and (*S*)-(−)-limonene complex with MD simulation. In this study, we used Adaptive Lambda Square Dynamics (ALSD) simulation^23,24^, which is one of MD simulation techniques that enhances conformational changes of substrates and/or enzyme active sites to achieve efficient docking pose search in a shorter time than conventional MD simulation (see “Methods” section for the details). Examples of docking poses corresponding to modification sites for the two target products, H6b (for *trans*-carveol (**12**) and H3a (for *cis*-isopiperitenol (**6**)), and the largest component of the byproduct docking pose group, H9b, are shown in Fig. 3A–C, respectively. In these docking poses, conformational differences are observed in the β-turn and the side chain ring of F87 in the pocket. For example, in Fig. 3A, the β-turn moves toward the pocket entrance side, and the ring of F87 moves to be parallel to the heme plane to invite the substrate to the backside. On the other hand, in Fig. 3C, the β-turn moves toward the backside, and the ring of F87 becomes perpendicular, similar to the X-ray crystal structure (Fig. 4B), preventing the substrate from entering the backside. These results indicate that it is necessary to consider not only the arrangement of the substrate relative to the enzyme but also the conformational change in the enzyme required to obtain the correct docking poses.

**Figure 5:**
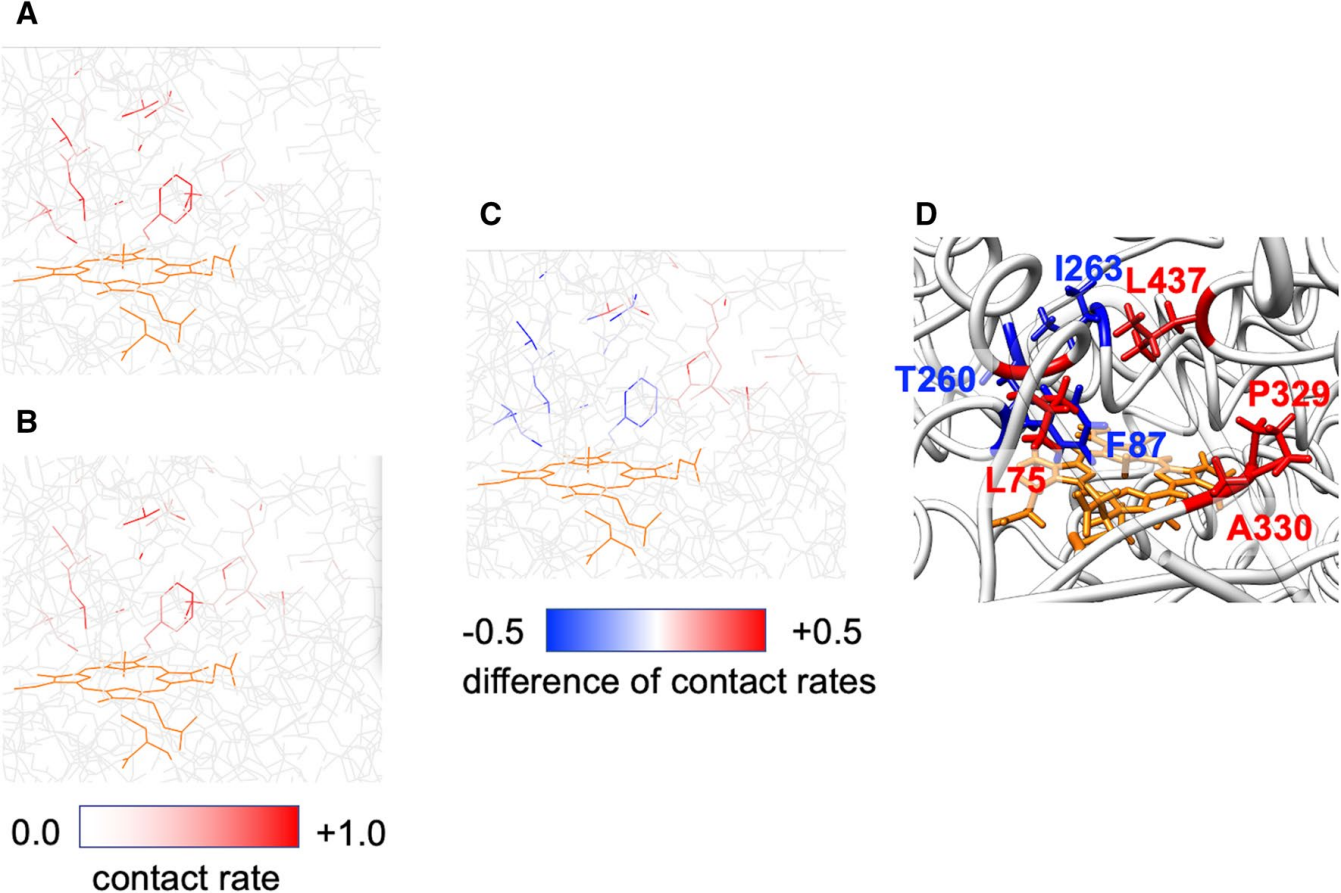
Contact rate maps of BMP atoms with (*S*)-(−)-limonene. The higher the contact rate of the atoms is, the redder the atoms are. A view of the active site from the F87 side. (**A**) The map from the docking poses where H6b of (*S*)-(−)-limonene contact the O atom bound to the Fe atom in heme, which corresponds to the generation of *trans*-carveol (**12**). (**B**) The map from the docking poses where one of the 15 hydrogen atoms located in the other oxidation sites (see Fig. 2) contacts the O atom, which corresponds to the generation of byproducts (**B**). (**C**) The difference map between (**B**) and (**A**). Red-colored residues are identified as candidate substitution residues for enhancing regioselectivity. These maps were calculated from the first ALSD simulation and projected on the X-ray crystal structure. (**D**) Positions of the top 4 (red) and the bottom 3 (blue) residues were determined by MSPER. A view of the active site from the pocket entrance side. A β-turn (K41-T49), the lid of the pocket entrance, is transparently displayed to make it easier to see around the active site.

To identify candidate substitution residues, we calculated the contact rates of BMP atoms with (*S*)-(−)-limonene in docking poses where the H6b of (*S*)-(−)-limonene contacts the O atom bound to the Fe atom in heme, which corresponds to *trans*-carveol (**12**) (Fig. 5A) and those in the docking poses where one of the 15 hydrogen atoms located in the other oxidation sites (see Fig. 2) contacts the O atom, which corresponds to the generation of byproducts (Fig. 5B). The examples of contact rate maps (Fig. 5A,B) and the difference in the maps (Fig. 5C) are shown. To ensure the prediction accuracy of MSPER, a ranked list was generated from each of the two independent simulations, and residues with the highest average rank were selected as candidate substitution residues. We selected the top four residues (A330, L75, P329, and L437, see Fig. 5D) from the ranked list (Table 1, Supplementary Table 2) as candidate substitution residues. In addition, we conducted proof experiments for the bottom three residues (F87, I263, and T260, see Fig. 5D) to confirm whether substitution of the bottom-ranked residues resulted in the reduction of *trans*-carveol. We also applied MSPER on the docking poses generated by a docking simulation software, ZDOCK (https://zlab.umassmed.edu/zdockconv3d/)^25^, to evaluate the effect of calculation methods used to generate docking pose data on the generated poses and the mutation site rankings. Although there are some differences between docking poses by ZDOCK and those by ALSD, the ranked list using ZDOCK is almost equivalent to that using ALSD in this case. The candidate substitution residues we selected above (A330, L75, P329, and L437) are also ranked relatively high in that using ZDOCK (Supplementary Table 22; 1st, 6th, 5th, and 2nd, respectively). The 3rd (F331) and 4th (A328) mutation sites in the ranked list using ZDOCK are 9th and 6th in that using ALSD, respectively. For details of the ZDOCK calculation, see Supplementary Information.

**Table 1:** *S*_scr_s and mutation site rankings for *trans-*carveol (**12**) based on *S*_scr_s.

Residue name	Ranking average	S_scr_ ranking		S_scr_	
		1	2	1	2
A330	1.5	1	2	0.806	0.855
L75	2.5	4	1	0.492	1.266
P329	3.5	3	4	0.508	0.654
L437	3.5	2	5	0.599	0.640

**Figure 6:**
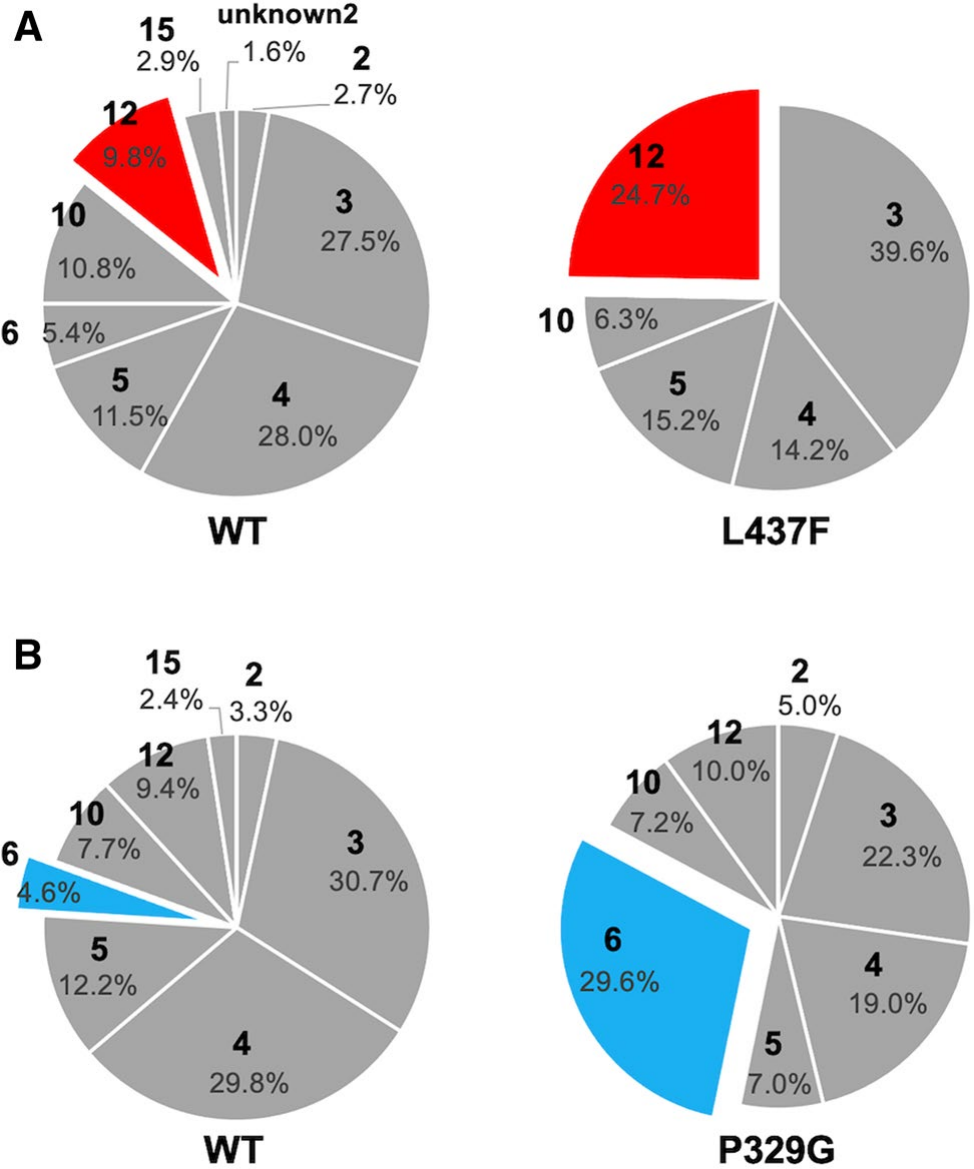
Comparison of reaction product compositions between wild-type CYP102A1 and CYP102A1 mutants. The percentages of oxidized compounds, *cis*-isopiperitenol (**6**) and *trans*-carveol (**12**), were calculated from the peak area of converted total products. These compound ratios of mutants were compared to those of wild type as the positive control for each experiment. (**A**) The data for the L437F mutant are presented as the averages of three independent experiments. The standard deviations for the percentages are included in Supplementary Table 1. (**B**) The data for the P329G mutant are presented as the averages of two independent experiments (Supplementary Table 11).

To investigate whether substitution of the top 4 residues enhances regioselective C6 (H6b as the hydrogen position) oxidation corresponding to *trans*-carveol as the reaction product (**12**), all single mutants for each position, the total number was 76, were constructed. The enzymatic activity of BMP in 53 of the single mutants yielded levels of *trans*-carveol above the experimental quantification limit. The *trans*-carveol reaction product ratio was increased by 81.1% (43 mutants) of the total activated mutants compared with the wild-type control (Supplementary Tables 7, 11, 12, and 13). At the top 4 residues, the A330P, L75F, P329L, and L437F mutants converted the maximum *trans*-carveol ratios, with *trans*-carveol comprising 16.1, 13.3, 22.5, and 24.7% of the reaction compounds, respectively, produced by these mutants (Table 2, Supplementary Fig. 5, and Supplementary Table 1). In particular, the L437F mutant produced 2.5-fold more *trans*-carveol (**12**) than the wild-type enzyme (Fig. 6A). In contrast, all mutants with substitutions at the bottom-ranked F87, I263, and T260 (i.e., 57 mutants) converted (*S*)-limonene, whereas 55 of these mutants yielded a lower *trans*-carveol ratio than the wild-type enzyme (Supplementary Table 8, 9, and 10). These results suggest that our method succeeds in identifying proper modification sites for regioselective C6 oxidation.

**Table 2:** (*S*)-(−)-Limonene (**1**) C6 oxidation activity of the proposed mutants determined by MD simulation.

Sample	S_scr_ ranking average	12 (%)
WT		9.8 ± 0.1
A330P	1.5	16.1 ± 0.1
L75F	2.5	13.3 ± 0.4
P329L	3.5	22.5 ± 0.3
L437F	3.5	24.7 ± 0.2

Furthermore, we made ranked lists for regioselective C3 oxidation corresponding to *cis*-isopiperitenol (**6**) and H3a of (*S*)-(−)-limonene as the hydrogen position, and the top two residues (A74 and P329, Supplementary Table 3) were selected as candidate substitution residues. A total of 63.1% of all single mutants (24 of 38) yielded a higher reaction product ratio of *cis*-isopiperiteol (**6**), and the maximum ratio of *cis*-isopiperiteol, which was 6.4-fold more than that produced by the wild-type ratio, was produced by P329G, with *cis*-isopiperiteol comprising 29.6% of the reaction products (Fig. 6B and Supplementary Tables 6 and 11). Therefore, these results suggest that MSPER can control regioselectivity not only for C6 but also for C3 oxidation.

Next, we show the simulation results of the BMP-*p*-cymene (**16**) complex. We identified candidate substitution residues for two target alcohols, carvacrol (**22**) and thymol (**21**), as shown in Supplementary Tables 4 and 5, respectively. Conversion of *p*-cymene to carvacrol was observed for 92 of 95 mutants (Supplementary Tables 14, 15, 17, 18, and 19). Substitutions at 5 candidate residues, namely, L75, A74, A330, P329, and M354, yielded the maximum carvacrol ratios, with carvacrol comprising 15.7%, 25.8%, 22.9%, > 99%, and 19.6% of the reaction compounds produced by the L75W, A74H, A330P, P329K, and M354R mutants, respectively. The carvacrol ratios for all P329 mutants were higher than the carvacrol ratio for the wild-type enzyme, although the amount of carvacrol was decreased for 5 mutants, i.e., as P329K, P329Q, P329R, P329M, and P329H (Supplementary Table 17). Moreover, the ratio and amount of carvacrol produced by the P329Y and P329G mutants were threefold higher than those produced by the wild-type enzyme. Meanwhile, the maximum ratio of carvacrol produced by mutants with substitutions at the bottom-ranked I263 for carvacrol was 0.8-fold that produced by the wild-type enzyme. The amount of carvacrol produced was below the quantification limit for 10 mutants (Supplementary Table 16).

Subsequently, the regioselectivity of C3 oxidation corresponding to H3 and H5 as the hydrogen positions and thymol as the product (**21**) by the mutants modified at the top-ranked 3 residues, L437, P329, and A74, was evaluated (Supplementary Tables 14, 17, and 20). Although the thymol ratio for the L437 mutants was reduced by > 30%, the thymol ratio for the P329R and A74H mutants was the highest, reaching 4.5- and 2.4-fold of the ratio for the wild-type control, respectively. These results demonstrated that MSPER can identify mutation sites for controlling the regioselectivity of CYP102A1 despite a structural difference in the substrates.

## Discussion

In the current study, we applied MSPER to a low-regioselectivity enzyme, CYP102A1, and the two substrates, (*S*)-(−)-limonene (**1**) and *p*-cymene (**16**), to predict mutation sites to enhance the regioselectivity for four target products with different substrate reaction sites. MSPER significantly reduced the number of mutants tested by reducing the number of candidate substitution residues from the 455 residues that make up the enzyme to just a few residues. The 13 of the total 14 top-ranked mutation sites predicted by MSPER for the four target products succeeded in selectively enhancing the regioselectivity up to 6.4-fold. On the other hand, the substitution of the lowest-ranked residues caused a significant decrease in the production rates. As shown in Fig. 5D, even though the bottom-ranked F87 is spatially close to the top-ranked L75 and L437, their substitutions have opposite effects on the regioselectivity for the target product. These results indicate that MSPER can distinguish very detailed differences of substrate structures and the reaction sites, and can accurately predict mutation sites to enhance regioselectivity. In addition, although conventional rational designs may predict residues near the substrate as candidate substitution residues, our study suggests that substitution of residues in direct contact with the substrate in docking poses for target products may reduce the production.

The P329 appeared in both ranked lists for *trans*-carveol (**12**) and *cis*-isopiperitenol (**6**). When the internal ring of the *S*-(−)-limonene is hydroxidized (Fig. 3A,B), the *S*-(−)-limonene locates at the far side of the β-turn and does not make direct contact with P329, which is located at the entrance side of the active site. On the other hand, when the H9b, the terminal of the *S*-(−)-limonene, is hydroxidized (Fig. 3C), P329 has high contact rates with (*S*)-(−)-limonene. The substitution of P329 is expected to destabilize the H9b docking pose group, which is the largest component of the byproduct docking pose group, leading to a decrease in the production rate of byproducts. Thus, MSPER will be an effective method for enzyme modification to enhance regioselectivity, as it can automatically predict a ranking list of mutation sites without the need for a detailed analysis by experts.

MSPER is an analytical method for identifying candidate substitution residues based on conformational differences from docking poses between a target product and byproducts. The accuracy of MSPER depends on the accuracy of docking pose data. If accurate docking pose data for the target and byproducts are obtained by X-ray crystallography or NMR and so on, MSPER can identify appropriate candidate substitution residues. If not, docking simulations^26^ would be useful ways to generate docking pose data. (see Supplementary Information for an example of applying docking poses generated by a docking simulation software, ZDOCK 3.0.2 software^25^, on MSPER). In this case, the ranked list using ZDOCK is almost equivalent to that using ALSD. When docking a flexible substrate, or when the target enzyme must undergo large conformational changes to bind to the substrate, such as induced-fit binding, it is difficult to accurately predict the docking poses by docking simulations because proteins and compounds are regarded as rigid bodies in docking simulations. In such cases, it should be better to apply MD simulation, which can consider conformational changes of enzymes and the substrates. In this study, we generated docking poses by ALSD simulations^23,24^, which are one of MD simulation techniques that enhance conformational changes of substrates and/or enzyme active sites to achieve efficient docking pose search in a shorter time than conventional MD simulation. REST2^27^ simulation is more accessible as it can enhance conformational changes similar to ALSD and can execute on a free MD program, Gromacs^28^.

MSPER is sensitive to subtle changes in enzyme–substrate contact patterns in docking poses. Therefore, if you use computational methods such as docking simulations or MD simulations to generate docking poses, rather than X-ray crystal structures or NMR models, we recommend that you confirm whether the conformational sampling of the system is carried out well. In the current study, we have confirmed the convergence of MSPER ranking lists obtained from two independent MD simulations. Since MSPER scores, *S*_scr_, varies in value with small differences in docking poses, it is not advisable to look for an exact match of the scores. We recommend checking whether the top-ranked residues from the two simulations match rather than the scores themselves. If the ranked lists do not match well, more extended simulations are required until sufficient docking pose sampling is achieved.

To confirm whether the effective mutation sites predicted by MSPER can also be predicted by a conventional rational design method, we compared our results with those of HotSpot Wizard^18^. HotSpot Wizard uses protein structure information as input and predicts evolutionarily changing amino acid positions in the active site and the access tunnel to the site as hotspots (see Supplementary Information for the detailed setting). HotSpot Wizard proposed 17 residues as functional hotspots using PDB data of CYP102A1 (PDB ID: 1BU7)^20^ as input (Supplementary Fig. 16). P329, which improved production rates of all four of our target products with different substrate reaction sites on the internal ring, was ranked 5th. M354 and L437, which were predicted as candidate substitution residues to enhance the regioselectivity of carvacrol (**22**) and *trans*-carveol (**12**) were 7th and 14th, respectively. However, no other residues (A74, L75, and A330) were predicted that successfully increased production rates of the target products in our experiments. Note that it does not mean that HotSpot Wizard does not work correctly. HotSpot Wizard assigns highly mutable functional residues, of which substitutions are unlikely to impair the enzyme function as hotspots. Therefore, it does not specify whether the residues predicted as hotspots will increase the production of either the target product or the byproducts, and the final selection of effective mutation sites will depend on the results of directed evolution screening experiments. On the other hand, MSPER aims to increase the production rate of the target product, or in other words, to decrease the production of byproducts while maintaining the production of the target one as much as possible. The difference in the design philosophy of the two methods is reflected in the difference in the results of the predicted candidate substitution residues. As a result, MSPER could predict effective mutation sites which were not predicted by HotSpot Wizard, to enhance the regioselectivity with high accuracy.

When identifying substitution residues by MSPER, it is essential to ensure that the suggested residues are involved in the enzyme’s catalytic activity. It should be pointed out that MSPER does not consider quantum chemical effects in the catalytic activity of enzymes because it suggests substitution residues only regarding conformational information. Even if enzyme mutants suggested by MSPER reduce the formation of docking poses for byproducts, the mutants may not generate the target product if residues in the active site are substituted. In this work, the residue corresponding to the active site (CYS 400 covalently bonded to heme) was removed from the ranked list in advance.

In this study, we evaluated the enzymatic reaction activity of single mutant enzymes suggested by MSPER. If the performance of the enzymes is insufficient for industrial use, it is necessary to develop multiple mutant enzymes by further substitutions. Based on docking poses of single mutants identified the high performance by experiments, MSPER can predict the candidate substitution residues for effective double mutants. By repeating the identification of effective mutants by experiments and the prediction of candidate substitution residues by MSPER, it is possible to propose effective multiple mutants.

MSPER is a mutation site prediction method and does not specify residue species substituted. In enzyme modification studies, in addition to the selection of mutation sites, it is also important to determine amino acid species to be introduced. For example, in the study of P450 by Seifert et al*.*^19^, amino acid species substituted were limited only to five hydrophobic residues (alanine, valine, phenylalanine, leucine, and isoleucine) because hydrophobic residues are preferentially found at substrate-interaction hotspot positions. Such a method is an effective way to reduce the size of mutant libraries for directed evolution, and when combined with MSPER, it may further reduce the number of mutants evaluated.

## Methods

### Generation of docking poses

In cytochrome P450 BM3 (CYP102A1) from *Bacillus megaterium*, the N-terminal P450 heme domain (BMP), which catalyzes substrates, and the C-terminal reductase domain (BMR), which transfer electrons from NADPH to BMP, are fused in a single polypeptide chain. Although the crystal structures of BMP and BMR have already been determined, the structure of full-length CYP102A1 has not been determined. Thus, we used the conformation of the BMP with the resolution of 1.65 Å (PDB ID: 1BU7)^20^, which is deposited in the RCSB Protein Data Bank (PDB) (http://www.rcsb.org/). Since the conformation does not include our target substrates, we generated the docking poses by a computational method.

Among computational methods, docking simulation^26^ is the most widely used method for predicting docking poses. Most docking simulations predict energetically stable docking poses by generating substrate conformations on the surfaces of rigid apoenzyme structures. Although docking simulation can predict docking poses quickly, it is difficult to consider conformational changes in enzymes and substrates. If an enzyme must undergo a conformational change to bind to the substrate, docking poses cannot be accurately predicted. For our target systems, we had difficulty making accurate docking poses for the generation of the target product and byproducts by docking simulations because the docking required conformational changes of the enzyme, as shown in Fig. 3. Thus, we applied molecular dynamics (MD) simulation^29,30^ to generate the docking poses. MD simulations can be used to calculate forces acting on atoms constituting molecules, track conformational changes over time, and consider conformational changes in enzymes and substrates, In the current work, we used adaptive lambda square dynamics (ALSD) simulation^23,24^, which is an improved MD simulation technique, to search for docking poses more quickly while considering conformational changes. The ALSD simulation allows an exhaustive search of docking poses in a practical simulation time of several days to a week (less than 5 days for each system in the current study) by promoting conformational changes in the substrate.

### MD simulation

We carried out MD simulations of a complex consisting of the 455 residues of BMP and its substrate, (*S*)-(−)-limonene (**1**), to determine the docking poses. A tentative conformation of the BMP and (*S*)-(−)-limonene complex was generated by inserting (*S*)-(−)-limonene into the BMP binding pocket (Fig. 4A,B) with ZDOCK 3.0.2 software^25^. The coordinates of the missing hydrogen atoms in the BMP crystal structure were automatically generated by an MD simulation program PRESTO ver. 3^31^. The complex was immersed in a water sphere: the center was the geometric center of mass, the radius was 49.5 Å, and the water molecules were equilibrated at 300 K and 1.0 g/cc in advance. Water molecules overlapping the complex were removed. Some water molecules were replaced with Na^+^ and Cl^−^ ions to neutralize the net charge and bring the ion concentration closer to the physiological one (0.153 M). Ultimately, the complex system consisted of 49,940 atoms (7390 atoms for BMP, 26 atoms for (*S*)-(−)-limonene, 31 Cl^−^ ions, 46 Na^+^ ions, and 42,447 atoms for water) (Fig. 4C). After energy minimization calculation, the system’s conformation was used as a pre-initial conformation to generate initial conformations for MD simulations. Similarly, the pre-initial conformation of another substrate, *p*-cymene (**16**), consisted of 49,935 atoms (7390 atoms for BMP, 24 atoms for *p*-cymene, 31 Cl^-^ ions, 46 Na^+^ ions, and 42,444 atoms for water).

We used an MD simulation program PRESTO ver. 3^31^ and the force field parameters for the BMP, substrates, water molecules, and ions were taken from the Amber-based hybrid force field (ω = 0.75)^32^, Amber gaff^33^, TIP3P^34^, and Joung-Cheatham^35^, respectively. The parameter for heme with an O atom bound to the Fe atom in the active site was taken from Shahrokh et al*.* 2012^36^. The substrate charges were determined as the restrained electrostatic potential (RESP) charges (see Supplementary Data 1 for details)^37^. During the MD simulations, a harmonic potential was applied to the water oxygen atoms only when they were outside the boundary to avoid evaporation of water molecules from the water sphere boundary (Fig. 4C). The simulation time step was 2 fs. The zero-dipole method^38,39^ was used to compute long-range electrostatic interactions. The cutoff distance for vdW and the electrostatic interaction was 12.0 Å. The SHAKE algorithm^40^ constrained covalent bonds involving hydrogen atoms. The constant-temperature method^41^ controlled the simulation temperature at 300 K.

### ALSD simulation

ALSD simulation^23,24^ is an MD simulation technique for an efficient conformational sampling of molecules such as multicanonical MD^42^ and replica exchange MD^43^. Here, we briefly explain the ALSD simulation technique. Please refer to the original paper^23^ for more information. ALSD simulation is an MD simulation technique used to enhance conformational changes in a predefined partial system (Region A, such as substrates). In contrast, the conformation of the rest of the system (Region B, such as the BMP and solvent) changes in the same way as in conventional MD. ALSD simulation enhances conformational sampling by introducing a fictitious *λ* particle with a fictitious mass, *m*_*λ*_. The atoms in the system and the *λ* particle move on Cartesian coordinates and a one-dimensional *λ* axis, respectively, obeying ALSD Hamiltonian (*H*_ALSD_),

4 $$\begin{array}{*{20}c} {H_{ALSD} = \lambda^{2} E_{AA} + \lambda E_{AB} + E_{BB} + K + m_{\lambda } \dot{\lambda }^{2} /2 + RTlnP\left( {\lambda ,{\text{T}}} \right)} \\ \end{array} ,$$

where *λ* is a coordinate of the *λ* particle; *E*_AA_, *E*_AB_, and *E*_BB_ are potential energy terms for intra-Region A, inter-Region AB, and intra-Region B, respectively; *K* and ${m}_{\lambda }{\dot{\lambda }}^{2}/2$ are the kinetic energy of the system and the *λ* particle, respectively; $\dot{\lambda }$ is the velocity of the *λ* particle; R is the gas constant; and T is the simulation temperature. In *H*_ALSD_, the potential energy terms related to Region A are scaled by *λ*^*2*^ and *λ* for *E*_AA_ and *E*_AB_, respectively. When $\lambda =1$, the potential energy in *H*_ALSD_ is the same as that in conventional MD. When $0<\lambda <1$, interactions involving Region A weaken, and conformational changes in Region A are enhanced. The last term in Eq. (4), $\mathrm{RTln}P\left(\lambda ,\mathrm{T}\right)$, is an artificial potential (umbrella potential^44^) that regulates the movement of the *λ* particle. If the a priori unknown function $P(\lambda ,\mathrm{T})$ is accurately estimated, the *λ* particle does a random walk, which enhances both the conformational change and sampling of the stable conformation for Region A in the predefined *λ* range ($0.6<\lambda <1.03$ in this work). In practical ALSD simulations, iterative runs of simulations are performed to estimate $P(\lambda ,\mathrm{T})$ before productive runs to obtain a conformational ensemble. The ensemble obtained from the productive run includes various conformations from unstable conformations at $\lambda <1$ to stable conformations at $\lambda \approx 1$. A realistic conformational ensemble at $\lambda =1$ can be reconstructed using a reweighting scheme^23^. We used the ensemble reweighted at $\lambda =1$ for the following analyses.

### Simulation procedure

We first carried out a trial ALSD simulation of the BMP and (*S*)-(−)-limonene (**1**) system, in which Region A was set as the substrate and the heme Fe and O atoms of the active site in BMP were given a fixed *λ* = 0.6 [Constant Lambda MD (CLMD)] to confirm whether the substrate can move enough around the active site. The results indicated that a side chain of F87 and a β-turn (T436–T438) protruding near the active site hindered the motion of (*S*)-(−)-limonene (data not shown). Thus, we finally set Region A as not only the substrate and the active site but also these protruding regions (Fig. 4A,B). The same setting for Region A was applied to the *p*-cymene (**16**) system.

To speed up the sampling, the ALSD simulation was combined with a parallel computing method, trivial trajectory parallelization (TTP)^45^. TTP allows highly efficient sampling of a conformational ensemble using *N* = 72 multiple independent simulations (in this work) starting from different initial conformations. To generate the initial conformations of the iterative ALSD simulation runs, we carried out 72 CLMD (*λ* = 0.6) simulations starting from pre-initial conformation with different initial velocities for 4 ns. Then, 30 cycles of the iterative ALSD runs and the following production ALSD run were performed to estimate $P(\lambda ,\mathrm{T})$ and to sample a conformational ensemble (the total simulation times of the iterative and production runs were $14.4 \mathrm{ns}\times 72 \mathrm{runs}=1036.8 \mathrm{ns}$ and $10 \mathrm{ns}\times 72 \mathrm{runs}=720 \mathrm{ns}$, respectively). A conformation was output every 2 ps, and the total number of output conformations was 360,000. To evaluate the statistical properties of the simulation results, we performed the ALSD simulation twice with different initial conformations for each substrate, and the total simulation execution time was 4.8 days for each system of the supercomputer system ITO at Kyushu University with 1,296 CPUs (Intel Xeon Gold 6154 (Skylake-SP) 3.0 GHz).

## Supplementary Information

Supplementary Information.
