# Author Correction: Enzyme modification using mutation site prediction method for enhancing the regioselectivity of substrate reaction sites

**DOI:** 10.1038/s41598-022-12278-2

**Published:** 2022-05-16

**Authors:** Jinzen Ikebe, Munenori Suzuki, Aya Komori, Kaito Kobayashi, Tomoshi Kameda

**Affiliations:** 1 Artificial Intelligence Research Center, National Institute of Advanced Industrial Science and Technology (AIST), 2-4-7 Aomi, Koto-ku, Tokyo, 135-0064 Japan; 2 KNC Bio-Research Center, KNC Laboratories Co., Ltd, 1-1-1 Murotani, Nishi-ku, Kobe, Hyogo 651-2241 Japan

Correction to: *Scientific Reports* https://doi.org/10.1038/s41598-021-98433-7, published online 04 October 2021

The original version of this Article contained errors in the Figure legends of Figure 2 and Figure 5b and in the Results section.

The legend of Figure 2

“Chemical structures of substrates, such as (**A**) (*S*)-(−)-limonene (**1**) and (B) *p*-cymene (**16**), with a number to each carbon and hydrogen atom. Seven hydrogen atoms (H2, H3a, H3b, H6a, H6b, H9a, and H9b) in (*S*)-(−)-limonene and all 14 hydrogen atoms in *p*-cymene (**16**) corresponding to experimentally detected oxidation sites were used to define the docking pose groups.”

now reads:

“Chemical structures of substrates, such as (**A**) (*S*)-(−)-limonene (**1**) and (B) *p*-cymene (**16**), with a number to each carbon and hydrogen atom. All 16 and 14 hydrogen atoms in (*S*)-(-)-limonene and *p*-cymene (**16**), respectively, were used to define the docking pose groups.”

The legend of Figure 5

“(**B**) The map from the docking poses where one of the six hydrogen atoms located in the other oxidation sites (see Fig. 2) contacts the O atom, which corresponds to the generation of byproducts (**B**).”

now reads:

“(**B**) The map from the docking poses where one of the 15 hydrogen atoms located in the other oxidation sites (see Fig. 2) contacts the O atom, which corresponds to the generation of byproducts (**B**).”

Furthermore, in the second paragraph of the Results section,

“For each conformation, we calculated the distances between the active site of the enzyme and substrate reaction sites (in this study, the oxygen atom covalently bonded to the Fe atom in heme and the hydrogen atoms located at the substrate reaction sites [7 and 14 hydrogen atoms in (*S*)-(−)-limonene (**1**) and *p*-cymene (**16**), respectively, as shown in Fig. 2)].”

now reads:

“For each conformation, we calculated the distances between the active site of the enzyme and substrate reaction sites (in this study, the oxygen atom covalently bonded to the Fe atom in heme and the hydrogen atoms located at the substrate reaction sites [16 and 14 hydrogen atoms in (*S*)-(−)-limonene (**1**) and *p*-cymene (**16**), respectively, as shown in Fig. 2)].”

And in the sixth paragraph of the Results section,

“To identify candidate substitution residues, we calculated the contact rates of BMP atoms with (*S*)-(−)- limonene in docking poses where the H6b of (*S*)-(−)-limonene contacts the O atom bound to the Fe atom in heme, which corresponds to *trans*-carveol (**12**) (Fig. 5A) and those in the docking poses where one of the six hydrogen atoms located in the other oxidation sites (see Fig. 2) contacts the O atom, which corresponds to the generation of byproducts (Fig. 5B).”

now reads:

“To identify candidate substitution residues, we calculated the contact rates of BMP atoms with (*S*)-(−)- limonene in docking poses where the H6b of (*S*)-(−)-limonene contacts the O atom bound to the Fe atom in heme, which corresponds to *trans*-carveol (**12**) (Fig. 5A) and those in the docking poses where one of the 15 hydrogen atoms located in the other oxidation sites (see Fig. 2) contacts the O atom, which corresponds to the generation of byproducts (Fig. 5B).”

The original Article has been corrected.

