# Supplementary material for: Enzyme modification using mutation site prediction method for enhancing the regioselectivity of substrate reaction sites
Source: Sci Rep. 2021 Oct 4;11:19004. doi: 10.1038/s41598-021-98433-7 (PMC8488038; doi:10.1038/s41598-021-98433-7)
Supplement: Supplementary file 1 — Supplementary Information. [file 41598_2021_98433_MOESM1_ESM.docx]

**Supporting Information**

**Enzyme modification using mutation site prediction method for enhancing the regioselectivity of substrate reaction sites**

Jinzen Ikebe^1^, Munenori Suzuki^2^, Aya Komori^2^, Kaito Kobayashi^1^, and Tomoshi Kameda^1*^

^1^Artificial Intelligence Research Center, National Institute of Advanced Industrial Science and Technology (AIST), 2-4-7 Aomi, Koto-ku, Tokyo 135-0064, Japan.

^2^KNC Bio-Research Center, KNC Laboratories Co., Ltd., 1-1-1 Murotani, Nishi-ku, Kobe, Hyogo, 651-2241, Japan.

**E-mail:** [**kameda-tomoshi@aist.go.jp**](mailto:kameda-tomoshi@aist.go.jp)

Chemicals

(*S*)-(-)-Limonene (**1**), (-)-carveol as a mixture of *trans*- and *cis*-isomers (**12**,**13**), (*R*)-(-)-limonene oxide, synonym of limonene 1,2-epoxide, as a mixture of *cis*- and *trans*-isomers (**2**, **3**), (*S*)-perillyl alcohol (**14**), *p*-cymene (**16**) and carvacrol (**22**) were purchased from FUJIFILM Wako Pure Chemical Corporation. (-)-Perillaldehyde (**7**), 4-isopropenyltoluene as a synonym of *p*-cymenene (**17**), cuminaldehyde (**18**), 4-isopropylbenzyl alcohol (**20**), and thymol (**21**) were purchased from Tokyo Chemical Industry Co., Ltd. Dipentene dioxide, a synonym of limonene diepoxide, as a mixture of isomers (**8**, **9**, **10**, **11**), (1*S*,2*S*,4*R*)-(+)-limonene-1,2-diol (**15**) and *p,α,α*-trimethylbenzyl alcohol (**19**) were purchased from Sigma-Aldrich.

Constructs

In the current study, BMP and BMR were expressed separately to correctly evaluate MSPER using an MD simulation in which BMR was removed. A DNA fragment encoding BMP was amplified via PCR with primers 1 and 2 (Supplementary Table 21). A fragment expressing the TEV cleavage site and a His6-tag was fused to the C-terminus of BMP (Supplementary Fig. 17) by PCR with primer 3. To construct the pRSF-BMPtevHis plasmid, the obtained fragment was introduced into multiple cloning site 1 of the pRSFDuet-1 vector (Merck) between the NcoI and BamHI restriction sites by using the NEBuilder Hifi DNA assembly system (New England Biolabs). Then, a DNA fragment encoding BMR of CYP102A1 amplified with the *lacZ* sequence (7 residue peptide) at the N-terminus (Supplementary Fig. 18) using primers 4 and 5 was introduced into multiple cloning site 2 of pRSF-BMPtevHis between the BglII and AvrII restriction sites by using the NEBuilder Hifi DNA assembly system (New England Biolabs). The obtained plasmid, pRSF-BMPtevHis/BMR, was used for functional analysis.

CYP102A1 mutants were constructed via two PCR steps using one forward and one reverse mutagenic primer for each site-directed mutant. The forward primers incorporating point mutations are described in Supplementary Table 21. The reverse primers were exactly complementary to the forward primers. The coding regions were fully sequenced to verify that PCR had introduced no undesired mutations.

Bioconversion of (*S*)-(-)-limonene and *p*-cymene by CYP102A1 and its mutants

*Escherichia coli* BL21(DE3) harboring pRSF-BMPtevHis/BMR or pRSF-mutated BMPtevHis/BMR was cultivated at 28 °C with shaking in 3 mL LB medium (10 g/L tryptone, 5 g/L yeast extract, 10 g/L sodium chloride) containing kanamycin (100 µg/mL). After 15 h of cultivation, 200 µL of culture was added to 20 mL of TB medium (12 g/L tryptone, 24 g/L yeast extract, 10 ml/L glycerol, 14 g/L dipotassium hydrogen phosphate, and 1.2 g/L potassium dihydrogen phosphate) supplemented with kanamycin (100 µg/mL), 5-aminolevulinic acid (80 µg/mL), ammonium iron(II) sulfate hexahydrate (100 µM) and isopropyl-*β*-D(-)-thiogalactopyranoside (50 µM). After 24 h of cultivation at 25 °C with shaking, the cells were collected (8,000 rpm, 10 °C, 10 min) and suspended in CV buffer (50 mM potassium phosphate buffer, pH 7.2, with 5% glycerol). The cell suspension analyzed by SDS-PAGE (Supplementary Fig. 6-15). (*S*)-(-)-Limonene or *p*-cymene in ethanol was added at a final concentration of 1 mg/mL to 250 µL of the cell suspension (approximately OD600 of 150). To avoid product volatilization, the reactions were performed in 1.5-mL vials. The reaction mixtures were incubated for 20 h at 20 °C with shaking. After incubation on ice, 50 µL brine and 375 µL ethyl acetate were added and mixed for 10 min. After centrifugation at 10,000 g for 5 min at 4 °C, the organic solvent phase was recovered for GC-MS.

GC-MS

GC-MS data for monoterpenoid analysis were corrected with a GC-MS-QP2010Ultra instrument (Shimadzu Corporation) operated in electron impact mode (70 eV). An HP-Innowax capillary column (30 m, 0.25 mm i.d., 0.25 µm film thickness, Agilent Technologies) was used under the following conditions: injection port temperature, 250 °C; interface temperature, 225 °C; ion source temperature, 250 °C; oven temperature program: 46 °C to 220 °C at a rate of 3 °C/min, 220 °C to 250 °C at a rate of 30 °C/min, and hold for 5 min; split ratio, 1:10; carrier gas, helium; and linear velocity, 36.4 cm/sec. The mass spectrometer scanning speed was 4 scans/sec from m/z 40 to m/z 400. (*S*)-(-)-Limonene (**1**), *p*-cymene (**16**), and their oxidized products were identified by comparing their GC retention times and mass spectra to those of authentic compounds and reference data (Adams 2009^1^, Davis 1990^2^, the commercial database NIST2014 and Seifert, A. 2009^3^). The amount of (*S*)-(-)-limonene reaction compounds (**2**~**15**) was calculated by determining the amount from the peak areas of the total ion chromatogram based on the *trans*-carveol external standards (**12**). The quantitation limit of each compound 3 μg/mL of the equivalent amount for *trans*-carveol (**12**). Similarly, the amount of converted *p*-cymene compounds (**17**~**22**) was calculated based on carvacrol (**22**). The quantitation limit of each compound (**17**~**22**) was 1 µg/mL.

Generation of docking poses and mutation site rankings for *trans*-carveol (**12**) with ZDOCK

To evaluate the impact of calculation methods to generate docking pose data on the generated poses and the mutation site rankings, we performed docking pose generation with a docking simulation using ZDOCK software^4^. A cocrystal structure contained (*S*)-(-)-limonene^5^ (CCDC number: 1970877) was obtained from the Cambridge Crystallographic Data Centre (CCDC) (<https://www.ccdc.cam.ac.uk/>), and a structure of (*S*)-(-)-limonene was isolated for the docking simulation. In the docking simulation, we treated the crystal structures of the BMP (PDB ID: 1BU7)^6^ and (*S*)-(-)-limonene as rigid bodies and used the top 100 of the 1200 docking poses generated for the following analysis. We applied MSPER on these docking poses to predict mutation sites to improve the production rate of *trans*-carveol (**12**). Of these 100 docking poses, three poses belonged to the H6b docking pose group for *trans*-carveol (**12**), and 85 ones did to the byproduct one. In the H6b docking pose group, the small methyl group (carbon 7 in Fig. 2A) always faces the far side of the active site, because the β-turn and the ring of F87 in the crystal structure narrow the entrance as shown in Fig. 4. On the other hand, in the H6b docking pose group from ALSD simulation, the methyl group faces the entrance side of the active site in many docking poses as shown in Fig. 3A, unlike docking poses from ZDOCK, because ALSD simulation can consider conformational changes of the β-turn and the ring of F87. Thus, there are differences in docking poses generated by the two computational methods.

Despite the differences in docking poses, the top-ranked four mutation sites (A330, L75, P329, and L437) in the ranked list with ALSD (Supplementary Table 2) also rank relatively high (1st, 6th, 5th, and 2nd, respectively) in that by ZDOCK (Supplementary Table 22). As described in Discussion section, destabilizing of the H9b docking poses, which is the largest component of the byproduct docking pose group, is the most effective in increasing the production rate of *trans*-carveol (**12**) where the internal ring of the (*S*)-(-)-limonene is hydroxylated. The biggest difference between the H6b and H9b docking poses is the substrate location in the active site, and the difference in the substrate orientation between ALSD and ZDOCK do not seem to have a significant effect on the ranked list. Although the result shows that the docking poses generated by ZDOCK can be used to predict mutation sites in this case, we would like to emphasize that more accurate prediction of docking poses is required in cases where more detailed differences among docking poses would have a significant impact on the ranked list, such as the orientation of the substrate in this study. When docking a flexible substrate or when the target enzyme must undergo large conformational changes to bind to the substrate, such as induced-fit binding, it is difficult to accurately predict the docking poses by docking simulations because proteins and compounds are regarded as rigid bodies in docking simulations.

HotSpot Wizard

To compare MSPER with a conventional rational design, we predicted hotspots for CYP102A1 with HotSpot Wizard^7^ v3.1 developed by Damborsky’s group. HotSpot Wizard is a web server for automatic identification of hotspots and is available at https://loschmidt.chemi.muni.cz/hotspotwizard/. It implements the protein engineering protocol, which targets evolutionarily variable amino acid positions located in the active site or lining the access tunnels, and requires the protein structure as input. We used as input CYP102A1 conformation (PDB ID: 1BU7 chain A) the same as we used for MSPER. We used the default values for the options to run HotSpot Wizard are as follows: probe radius 2.8 Å for calculation of pocket, minimum probe radius 1.4 Å for calculation of tunnels and 3.5 Å for clustering threshold. Standard amino acids and HEM were included in the analysis.

**References**

1. Adams, R. P. *Identification of Essential Oil Components By Gas Chromatography/Mass Spectrometry*. (Allured Pub Corp, 2007).

2. Davies, N. W. Gas chromatographic retention indices of monoterpenes and sesquiterpenes on methyl silicon and Carbowax 20M phases. *J. Chromatogr. A* **503**, 1–24 (1990).

3. Seifert, A. *et al.* Rational design of a minimal and highly enriched CYP102A1 mutant library with improved regio-, stereo- and chemoselectivity. *ChemBioChem* **10**, 853–861 (2009).

4. Pierce, B. G., Hourai, Y. & Weng, Z. Accelerating protein docking in ZDOCK using an advanced 3D convolution library. *PLoS One* **6**, e24657 (2011).

5. Krupp, F., Frey, W. & Richert, C. Absolute Configuration of Small Molecules by Co-Crystallization. *Angew. Chemie - Int. Ed.* **59**, 15875–15879 (2020).

6. Sevrioukova, I. F., Li, H., Zhang, H., Peterson, J. A. & Poulos, T. L. Structure of a cytochrome P450-redox partner electron-transfer complex. *Proc. Natl. Acad. Sci. U. S. A.* **96**, 1863–1868 (1999).

7. Pavelka, A., Chovancova, E. & Damborsky, J. HotSpot Wizard: A web server for identification of hot spots in protein engineering. *Nucleic Acids Res.* **37**, (2009).

**
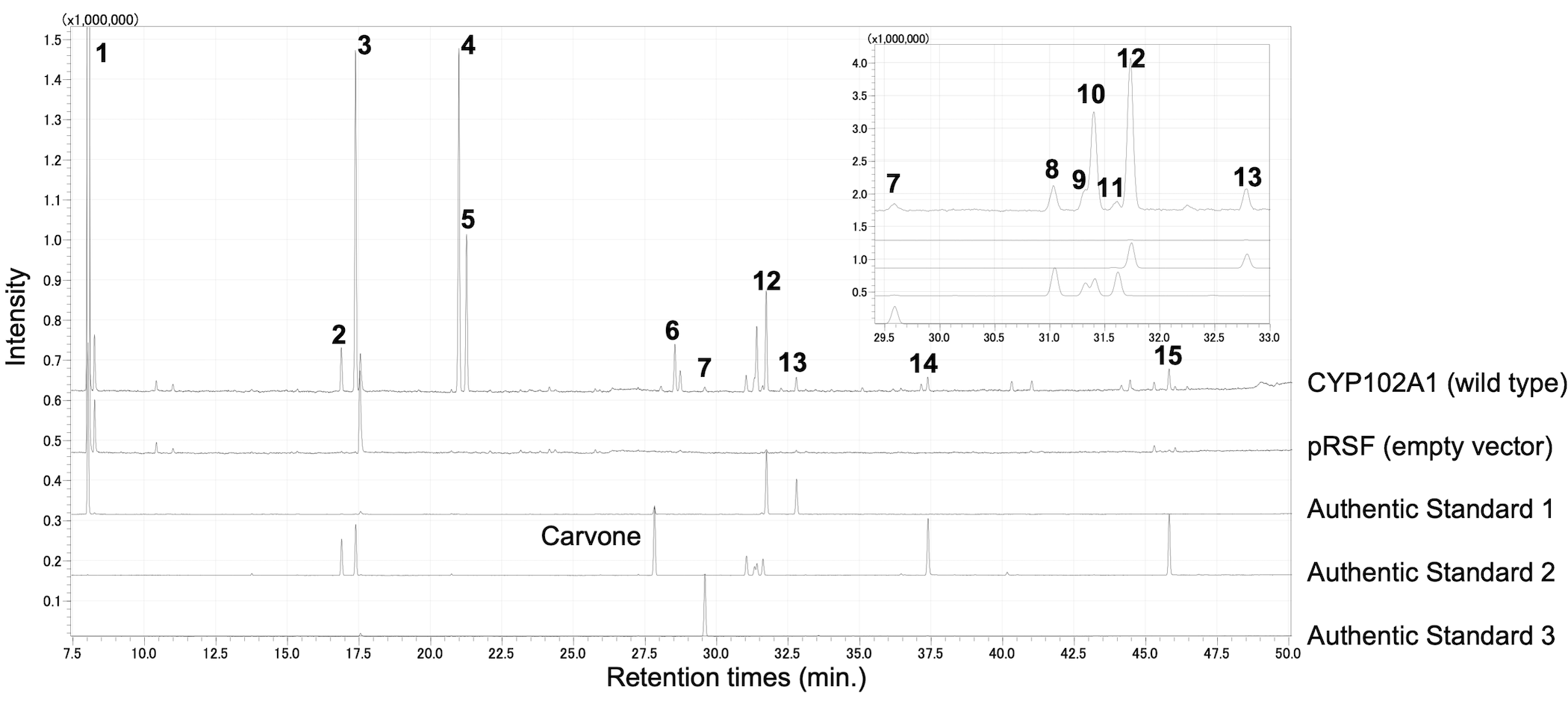
**

**Supplementary Fig. 1.** GC-MS analysis of biotransformation products from *E. coli* strains expressing CYP102A1 (wild type). (*S*)-(-)-Limonene (**1**) was converted to 14 oxidized compounds, i.e., limonene-1,2-epoxide and its isomer (**2**, **3**), limonene-8,9-epoxide and its isomer (**4**, **5**), *cis*-isopiperitenol (**6**), (-)-perillaldehyde (**7**), limonene diepoxide and its isomers (**8**-**11**), *trans*-carveol (**12**), *cis*-carveol (**13**), perillyl alcohol (**14**) and limonene-1,2-diol (**15**). Monoterpenoids were identified by comparing their GC retention times and mass spectra (Supplementary Fig. 2) to authentic compounds (**1**-**3** and **7**-**15**) or reference data (**4**-**6**). Authentic standard mixtures were as follows; mixture 1 included **1**, **12**, and **13**, mixture 2 included **2**-**3**, carvone, **8**-**11**, **14**, and **15**, mixture 3 included **7**.

**
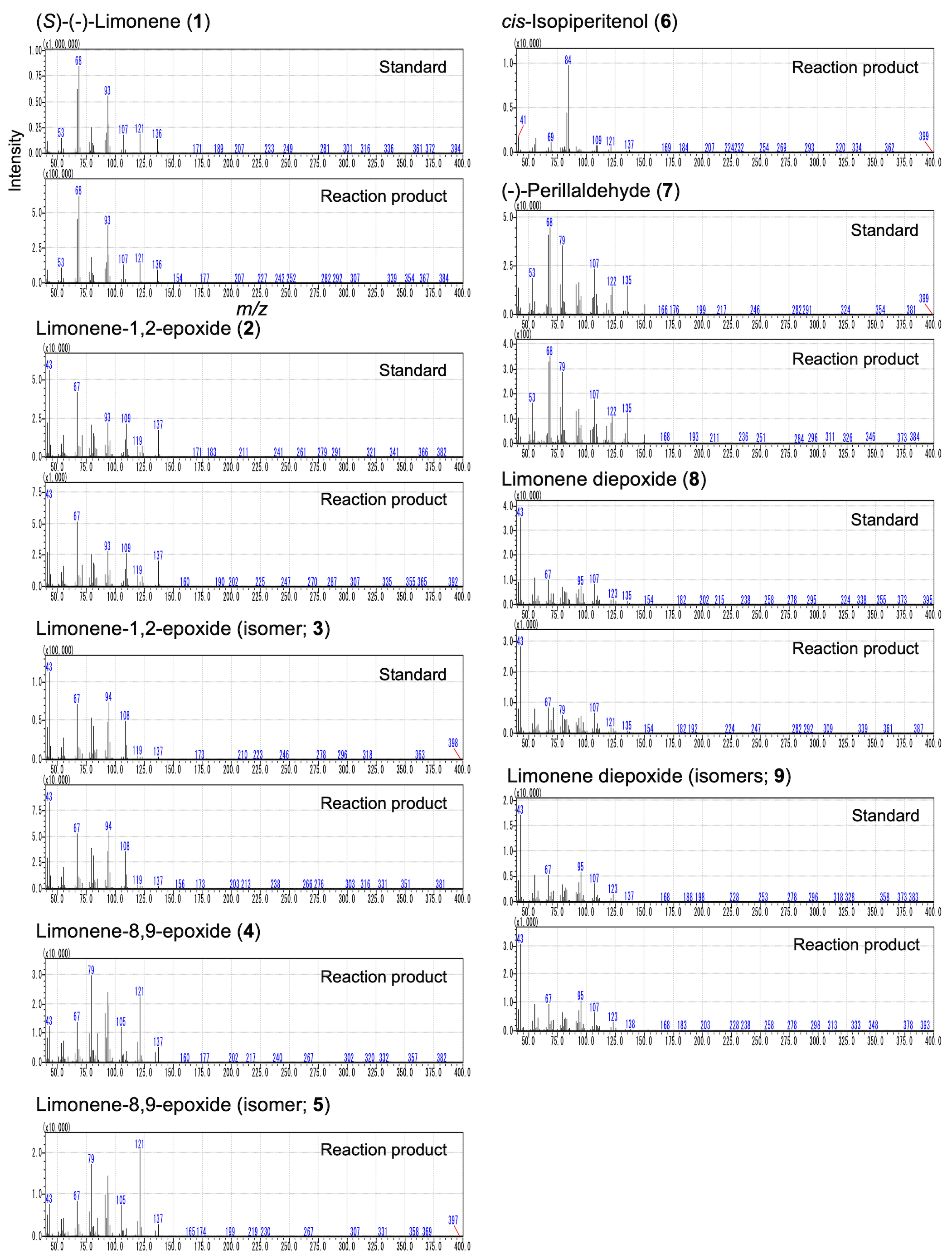
**

**Supplementary Fig. 2.** Fragmentation patterns in mass spectrometry with authentic standard and reaction product from *E. coli* strains expressing CYP102A1 (wild type).

(*continued*)


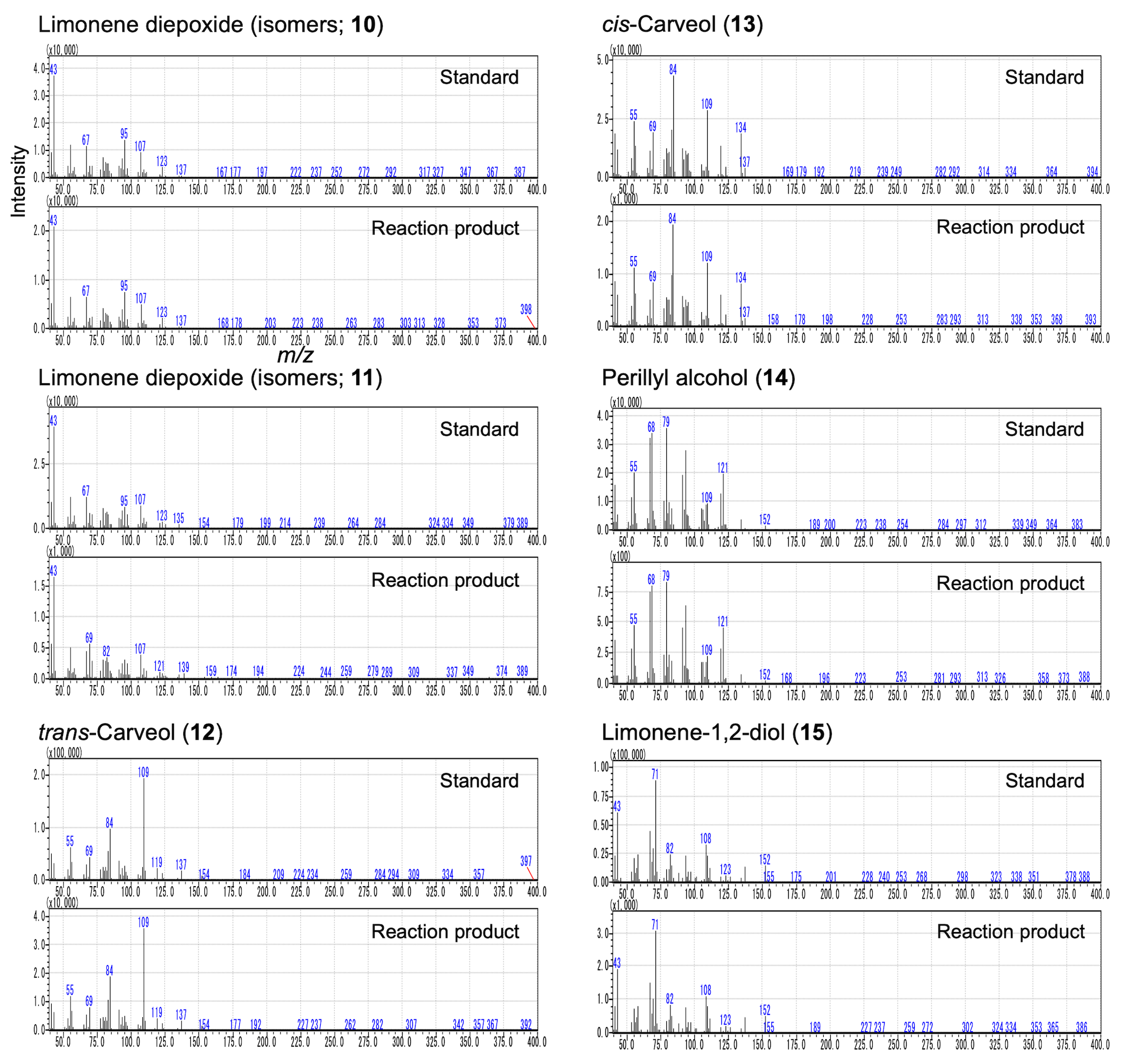


**Supplementary Fig. 2**. (*continued*)


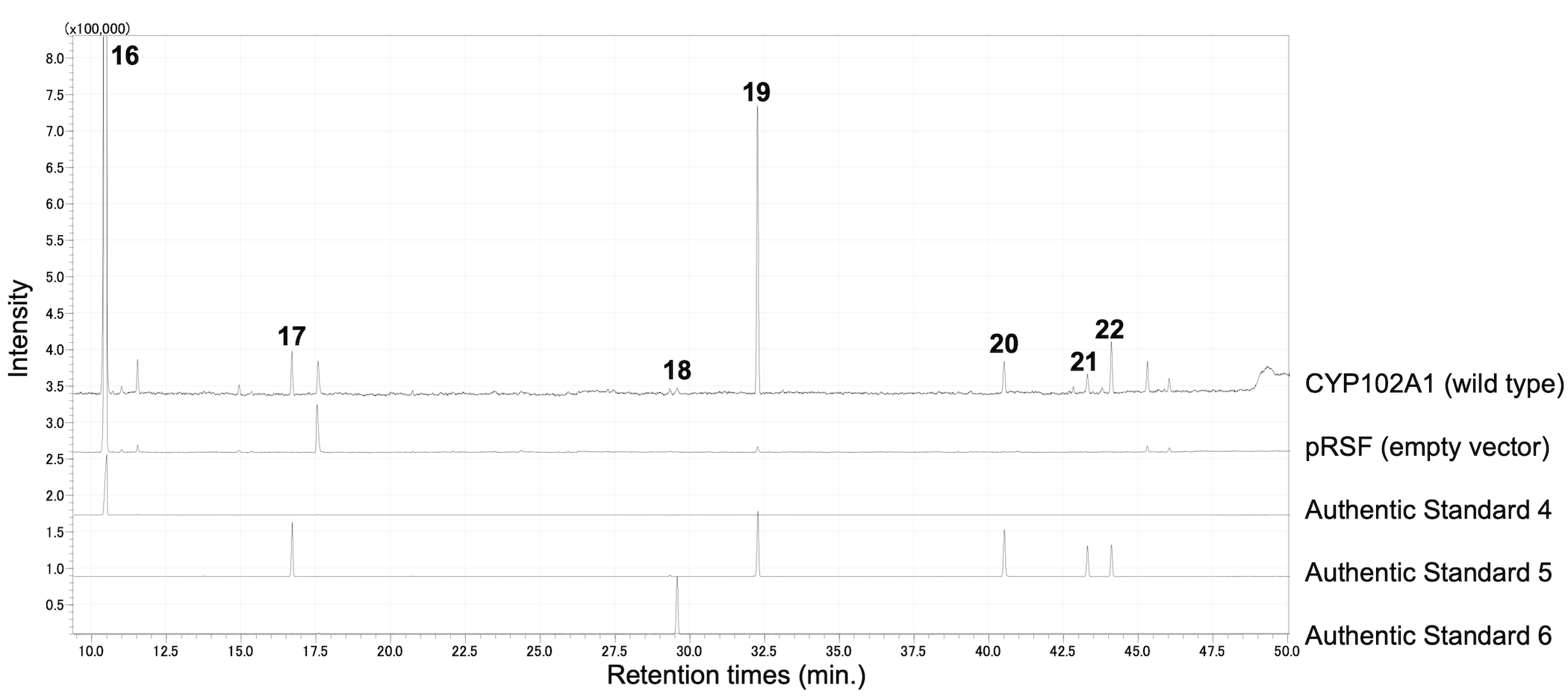


**Supplementary Fig. 3**. GC-MS analysis of biotransformation products from *E. coli* strains expressing CYP102A1 (wild type). The enzyme converted *p*-cymene (**16**) to six compounds, i.e., *p*-cymenene (**17**), cuminaldehyde (**18**), *p*,*α*,*α*-trimethylbenzyl alcohol (**19**), 4-isopropylbenzyl alcohol (**20**), thymol (**21**), and carvacrol (**22**). Substrate and reaction products were identified by comparing their GC retention times and mass spectra to authentic compounds (Supplementary Fig. 4). Authentic standard mixtures were as follows; mixture 4 included **16**, mixture 5 included **17** and **19**-**22**, mixture 6 included **18**.


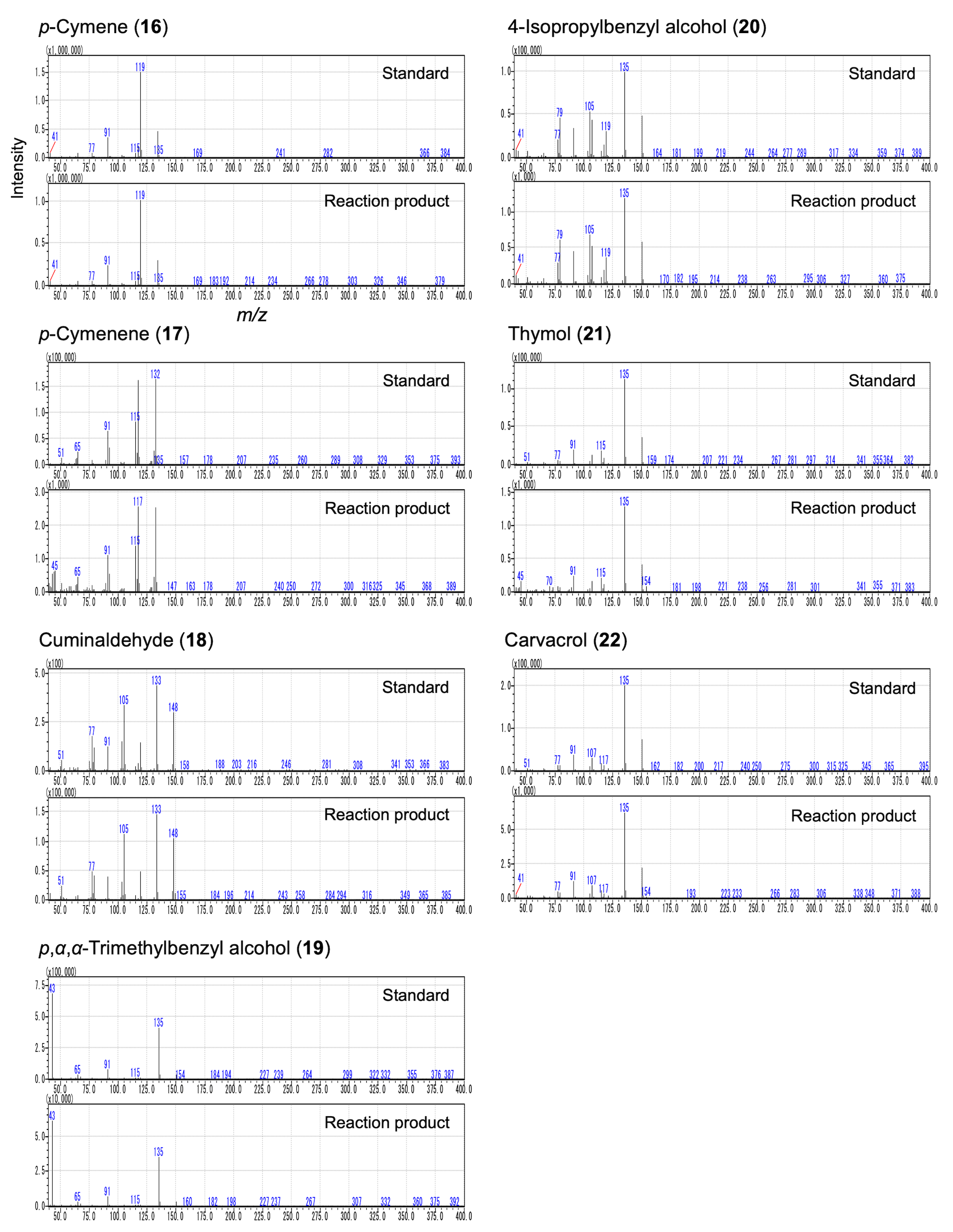


**Supplementary Fig. 4**. Comparison of fragmentation patterns in mass spectrometry between authentic standard and reaction product from *E. coli* strains expressing CYP102A1 (wild type).


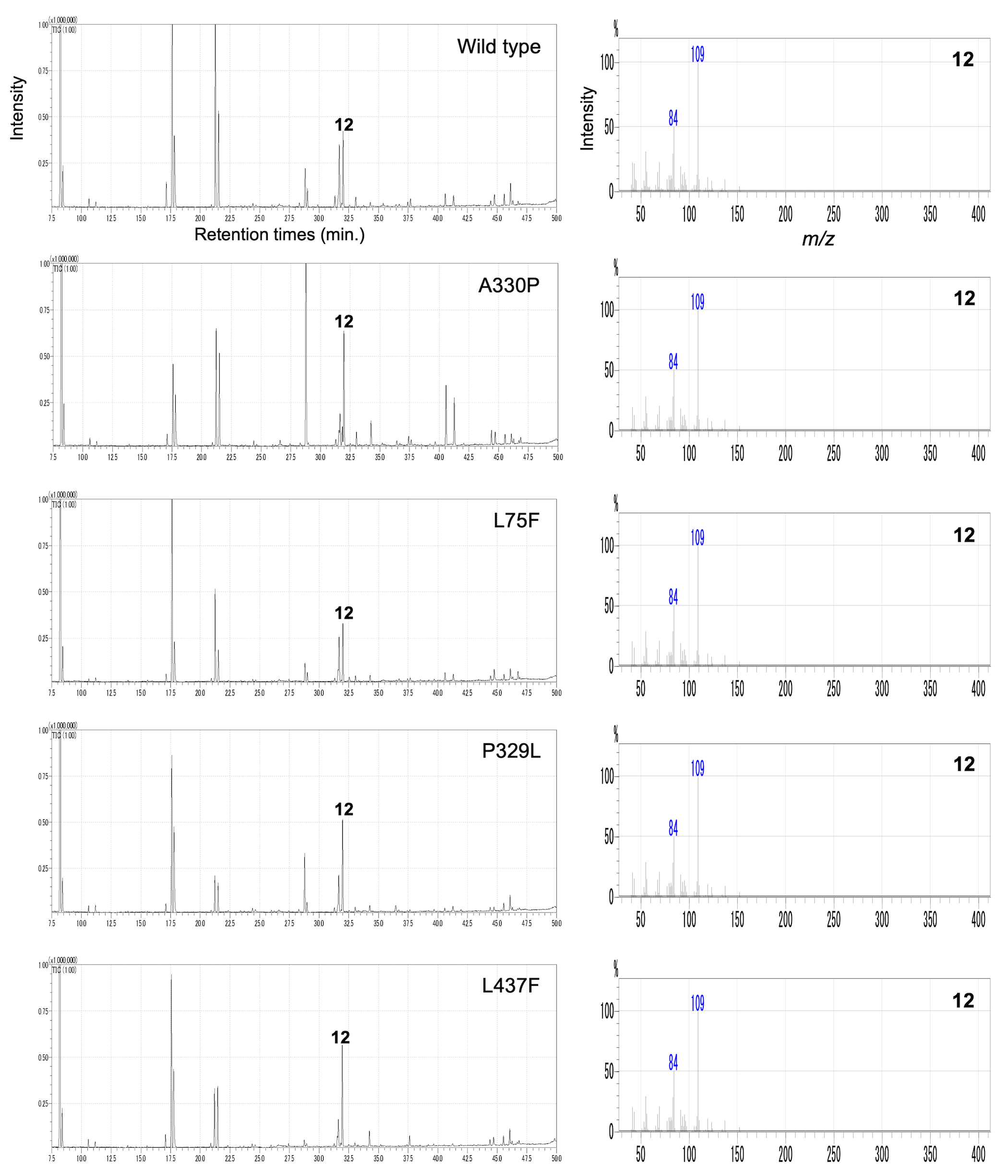


**Supplementary Fig. 5** Comparison of biotransformation product profiles with CYP102A1 wild type and its mutants by using GC-MS. (*S*)-(-)-Limonene C6 oxidized compound, as *trans*-carveol (**12**), was identified by retention time and mass fragment patterns.

**
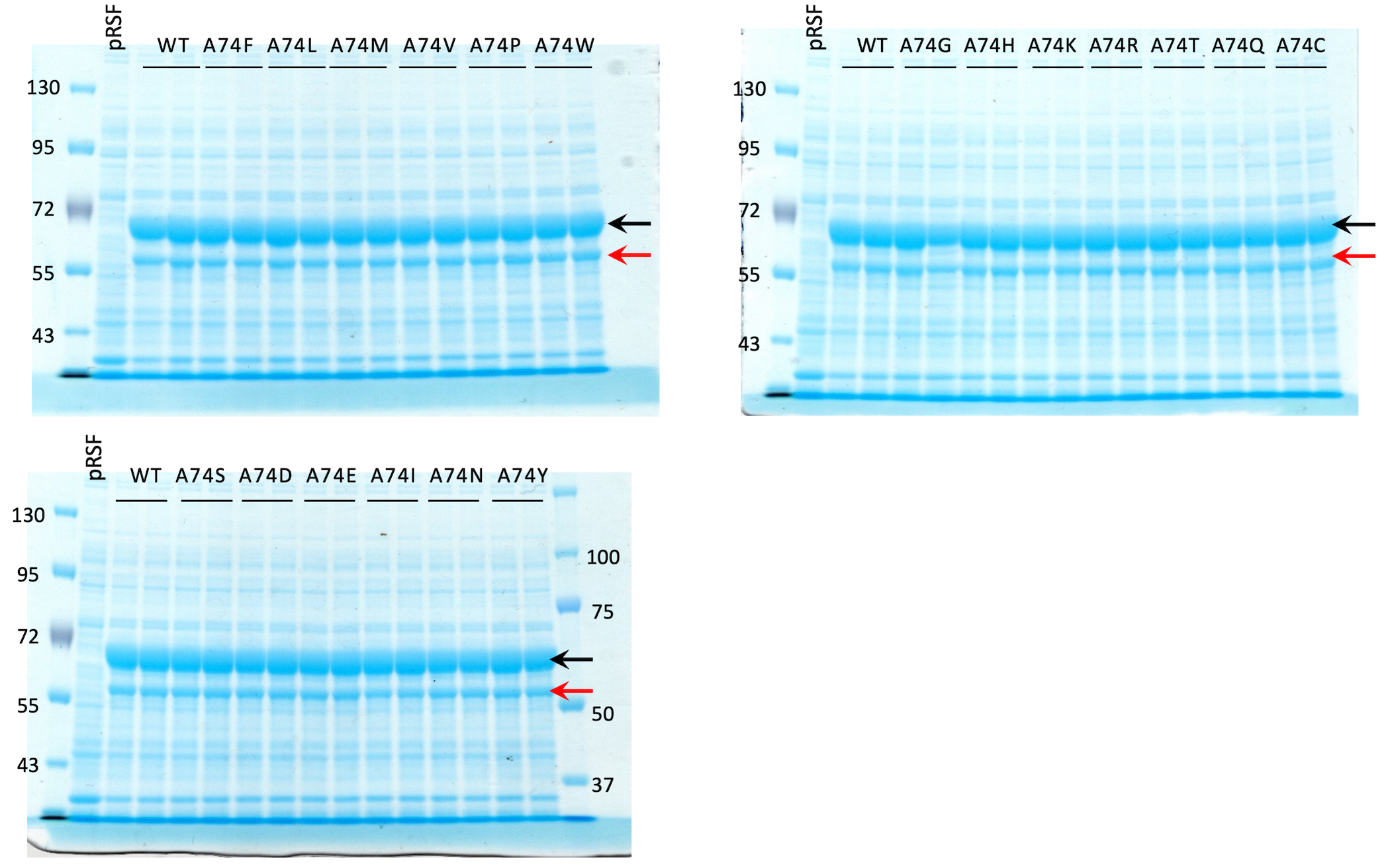
**

**Supplementary Fig. 6.** SDS-PAGE analysis of *E. coli* expressed CYP102A1 A74 mutants for (*S*)-(-)-limonene (**1**) and *p*-cymene (**16**) conversion. The expected positions of BMP and BMR are indicated by red and black arrows respectively. Lane pRSF: empty vector. Lane WT; wild type protein.

**
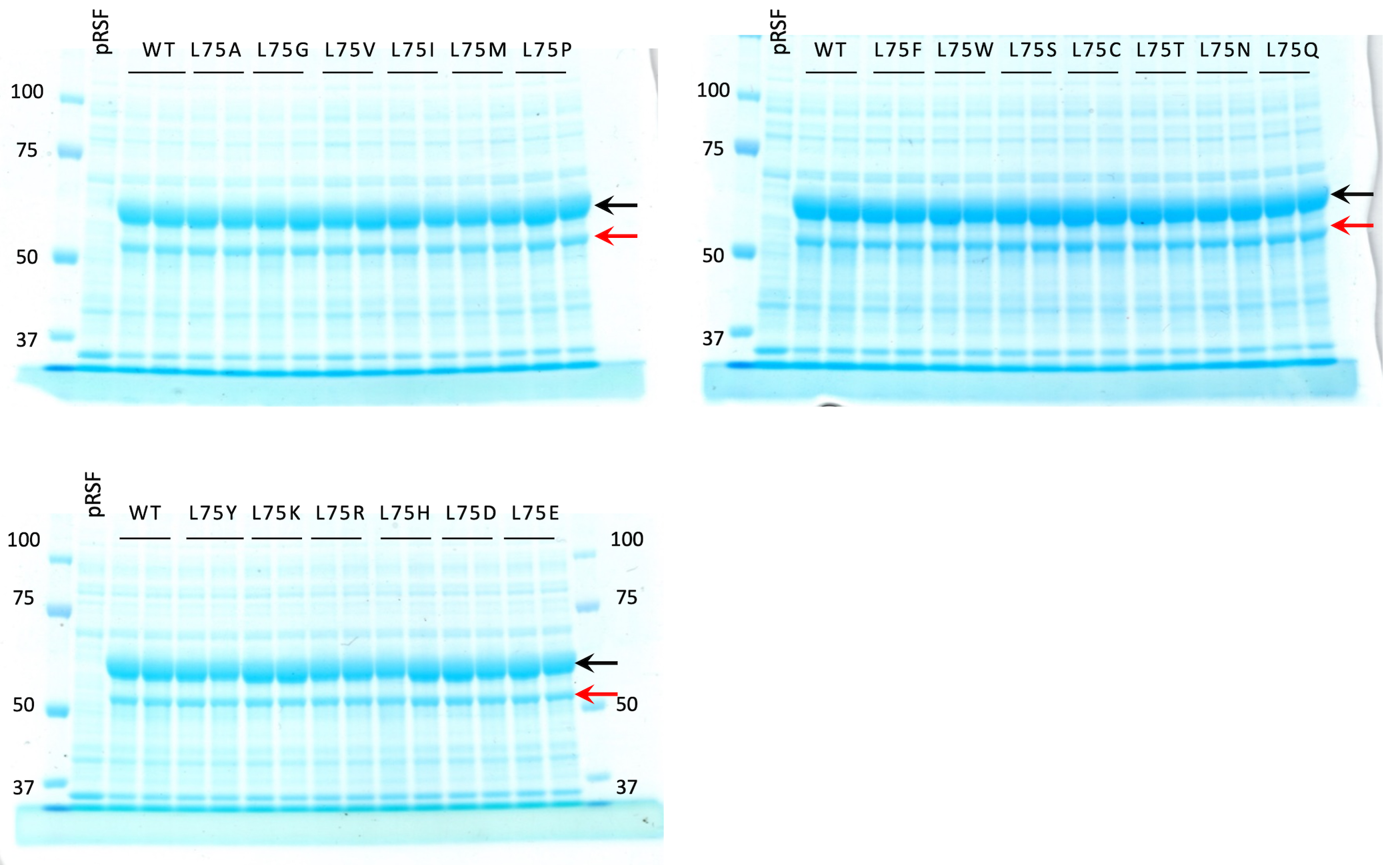
**

**Supplementary Fig. 7.** SDS-PAGE analysis of *E. coli* expressed CYP102A1 L75 mutants for (*S*)-(-)-limonene (**1**) and *p*-cymene (**16**) conversion. The expected positions of BMP and BMR are indicated by red and black arrows respectively. Lane pRSF: empty vector. Lane WT; wild type protein.


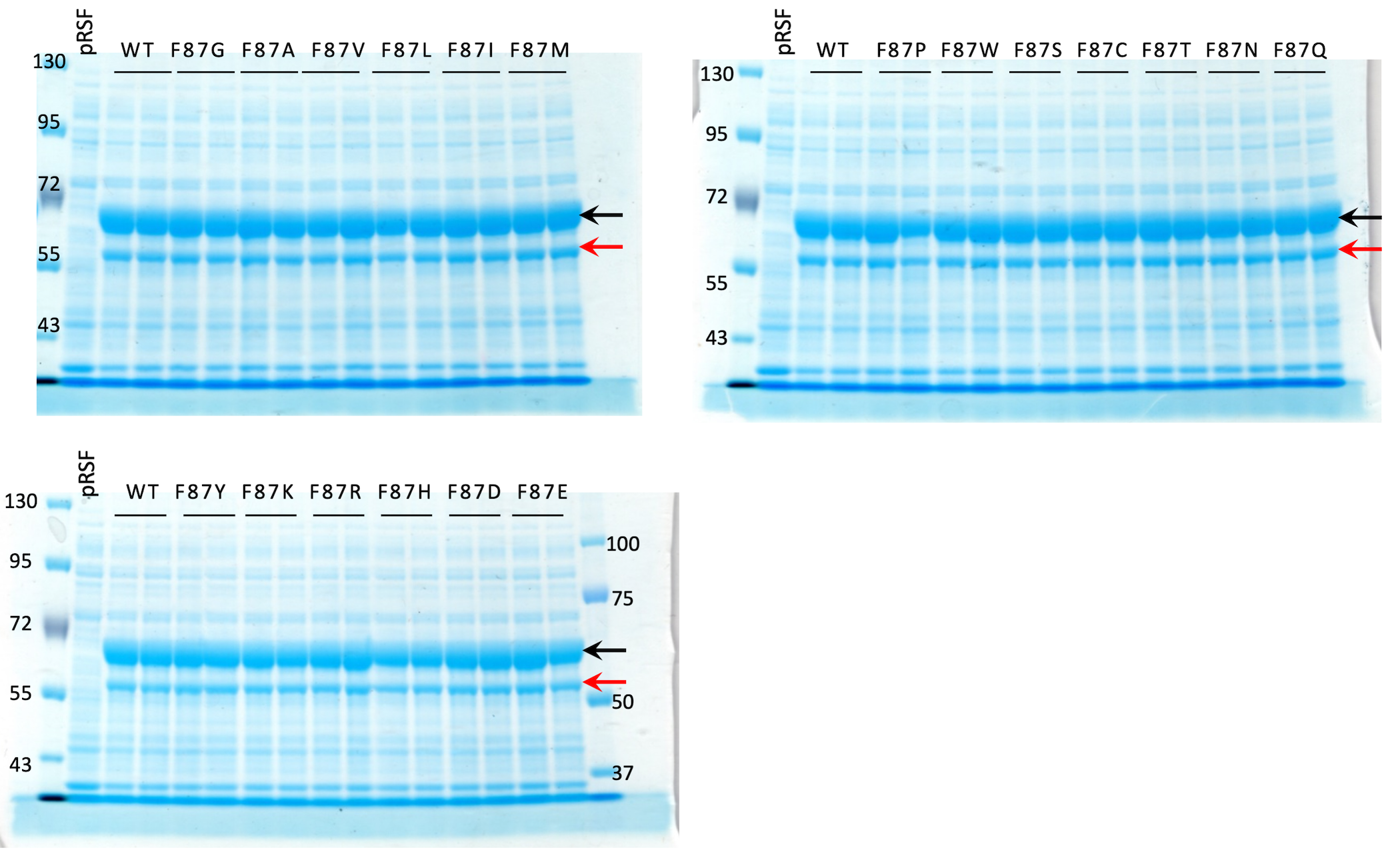


**Supplementary Fig. 8.** SDS-PAGE analysis of *E. coli* expressed CYP102A1 F87 mutants for (*S*)-(-)-limonene (**1**) and *p*-cymene (**16**) conversion. The expected positions of BMP and BMR are indicated by red and black arrows respectively. Lane pRSF: empty vector. Lane WT; wild type protein.


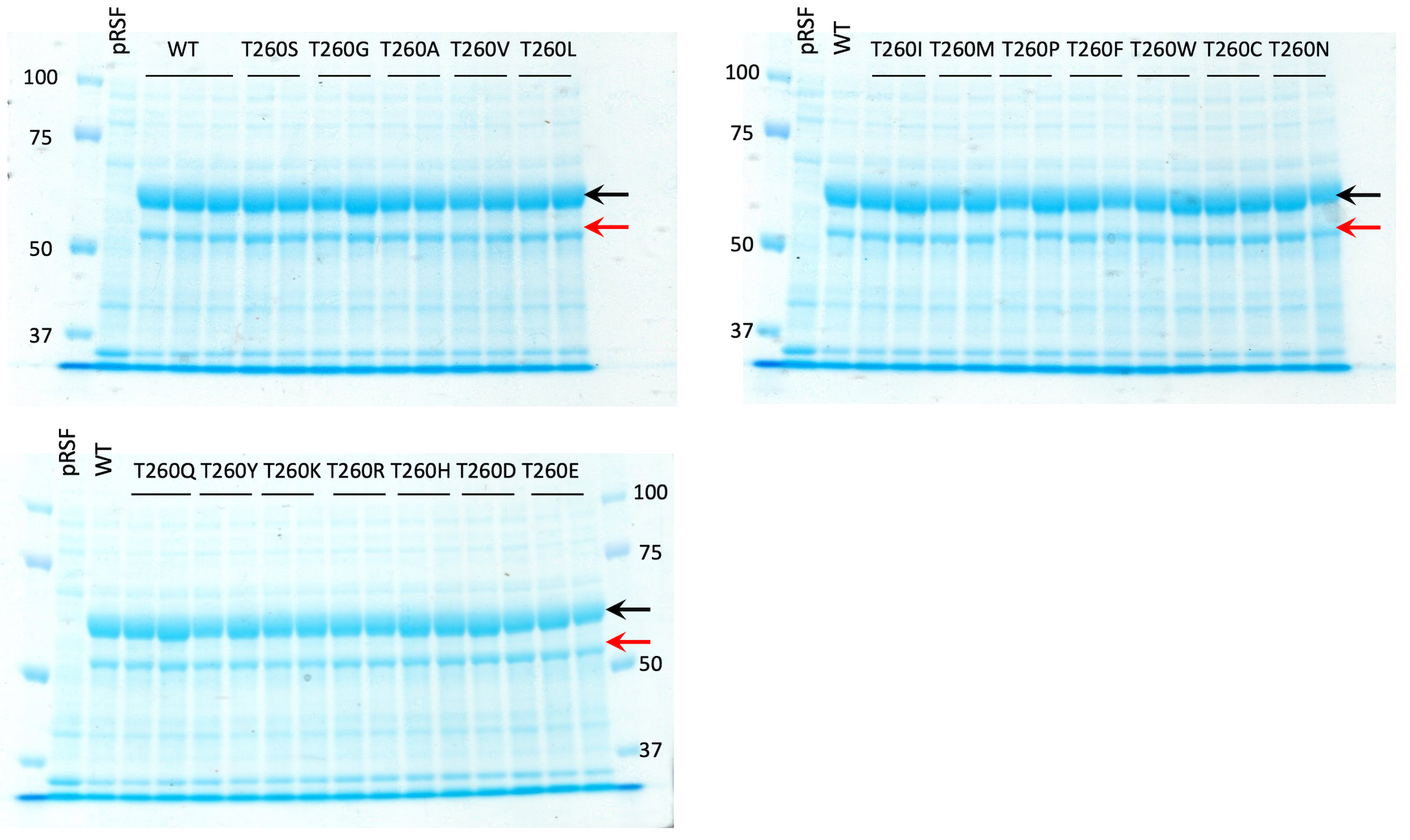


**Supplementary Fig. 9.** SDS-PAGE analysis of *E. coli* expressed CYP102A1 T260 mutants for (*S*)-(-)-limonene (**1**) and *p*-cymene (**16**) conversion. The expected positions of BMP and BMR are indicated by red and black arrows respectively. Lane pRSF: empty vector. Lane WT; wild type protein.


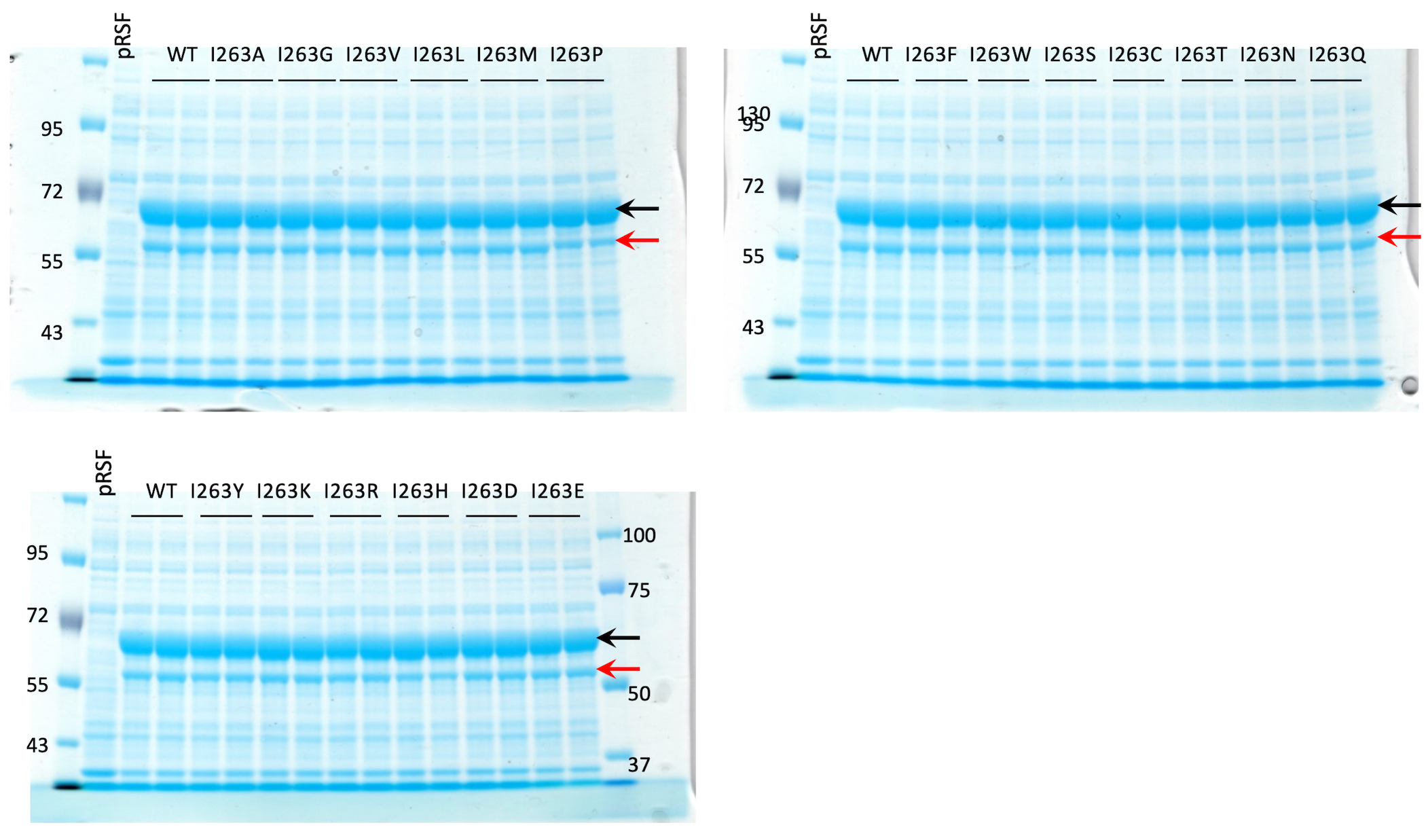


**Supplementary Fig. 10.** SDS-PAGE analysis of *E. coli* expressed CYP102A1 I263 mutants for (*S*)-(-)-limonene (**1**) and *p*-cymene (**16**) conversion. The expected positions of BMP and BMR are indicated by red and black arrows respectively. Lane pRSF: empty vector. Lane WT; wild type protein.


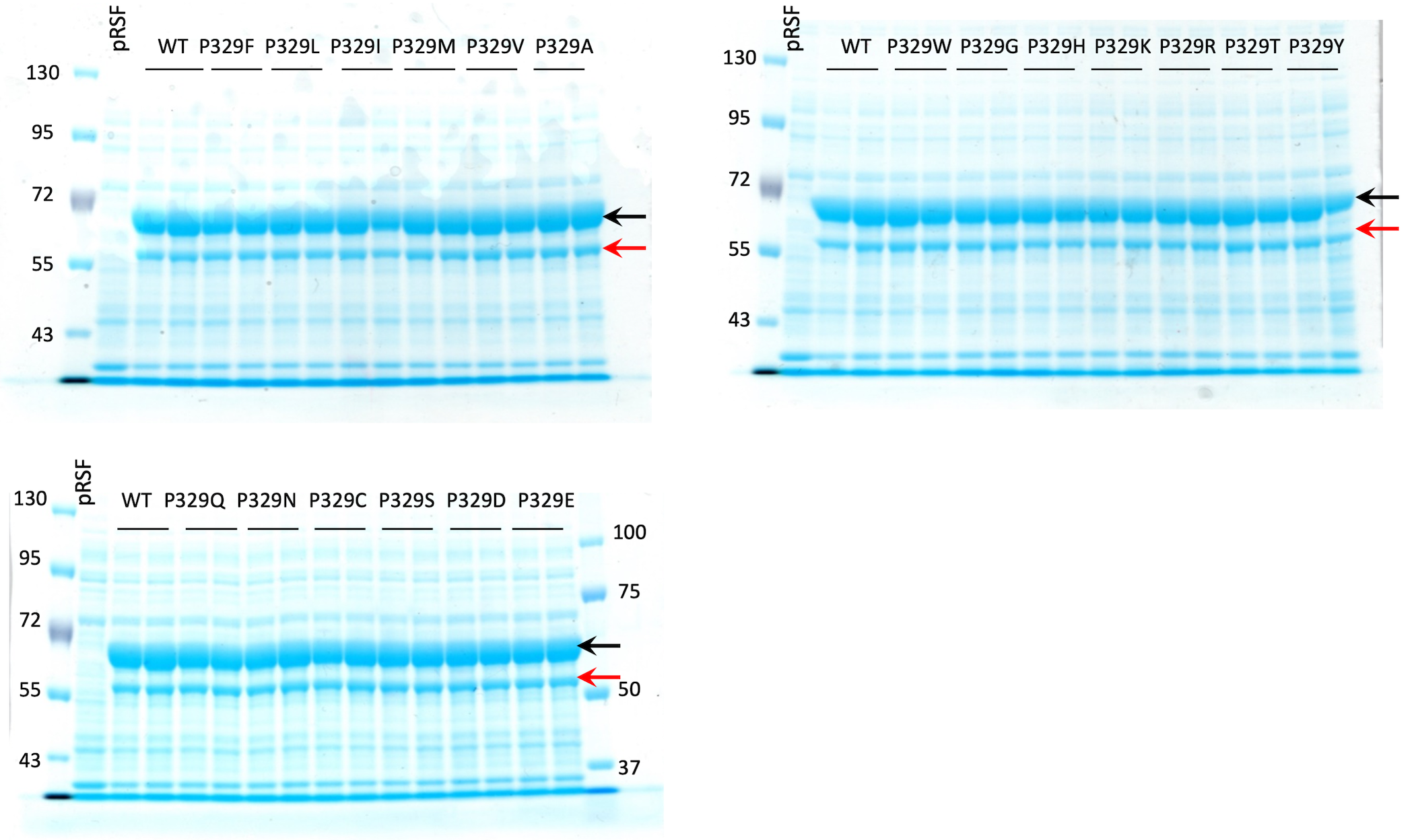


**Supplementary Fig. 11.** SDS-PAGE analysis of *E. coli* expressed CYP102A1 P329 mutants for (*S*)-(-)-limonene (**1**) conversion. The expected positions of BMP and BMR are indicated by red and black arrows respectively. Lane pRSF: empty vector. Lane WT; wild type protein.

**
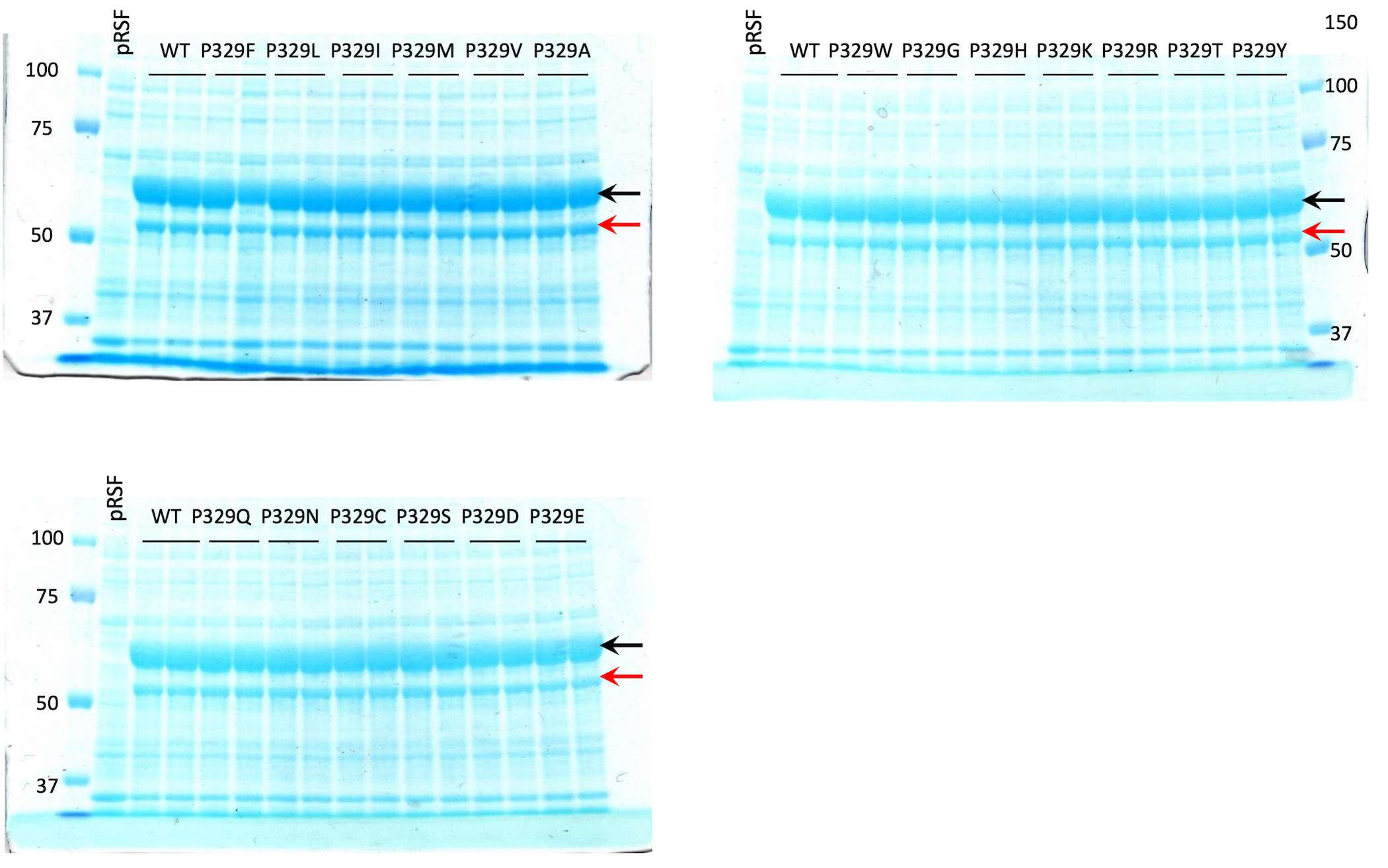
**

**Supplementary Fig. 12.** SDS-PAGE analysis of *E. coli* expressed CYP102A1 P329 mutants for *p*-cymene (**16**) conversion. The expected positions of BMP and BMR are indicated by red and black arrows respectively. Lane pRSF: empty vector. Lane WT; wild type protein.


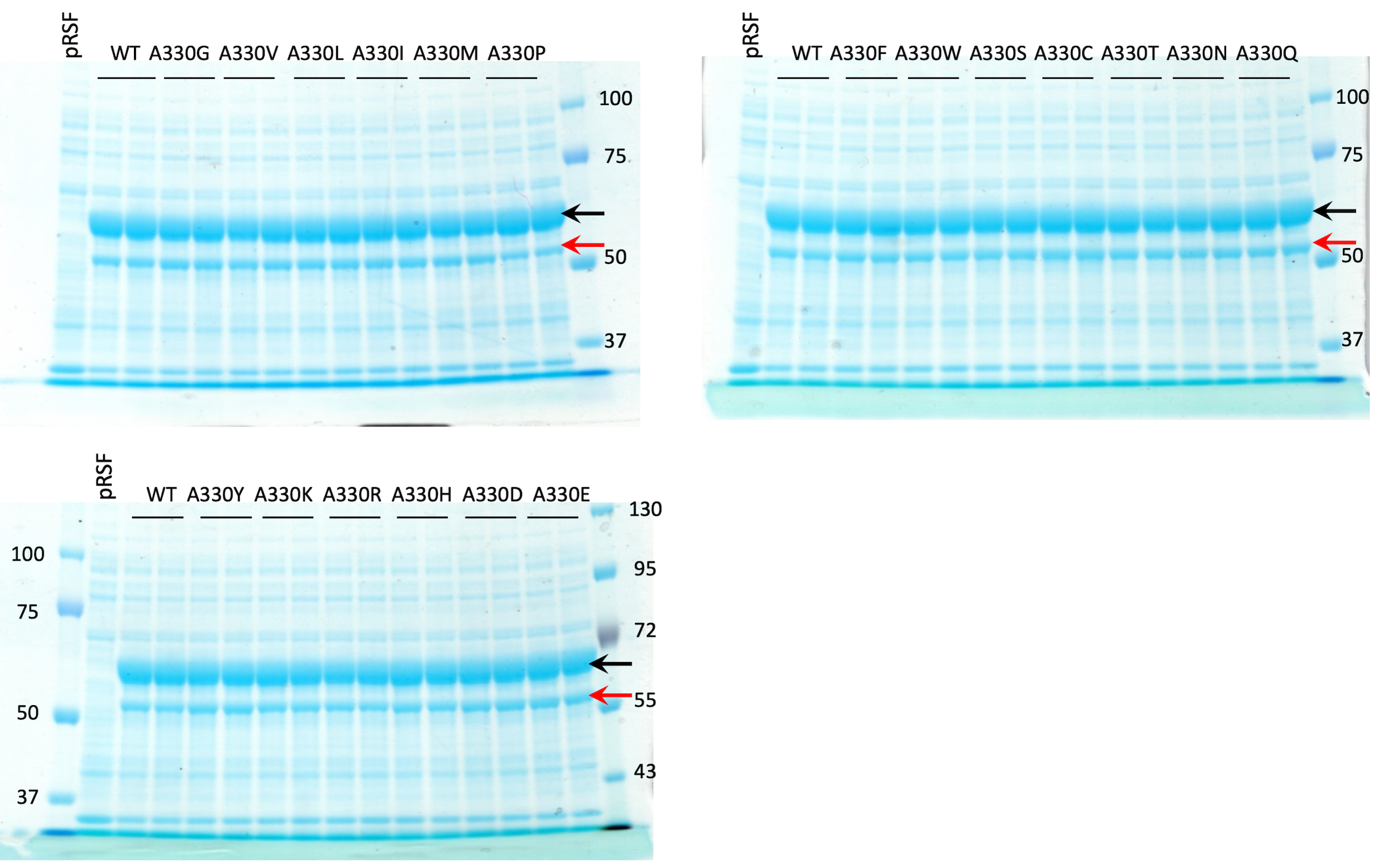


**Supplementary Fig. 13.** SDS-PAGE analysis of *E. coli* expressed CYP102A1 A330 mutants for (*S*)-(-)-limonene (**1**) and *p*-cymene (**16**) conversion. The expected positions of BMP and BMR are indicated by red and black arrows respectively. Lane pRSF: empty vector. Lane WT; wild type protein.


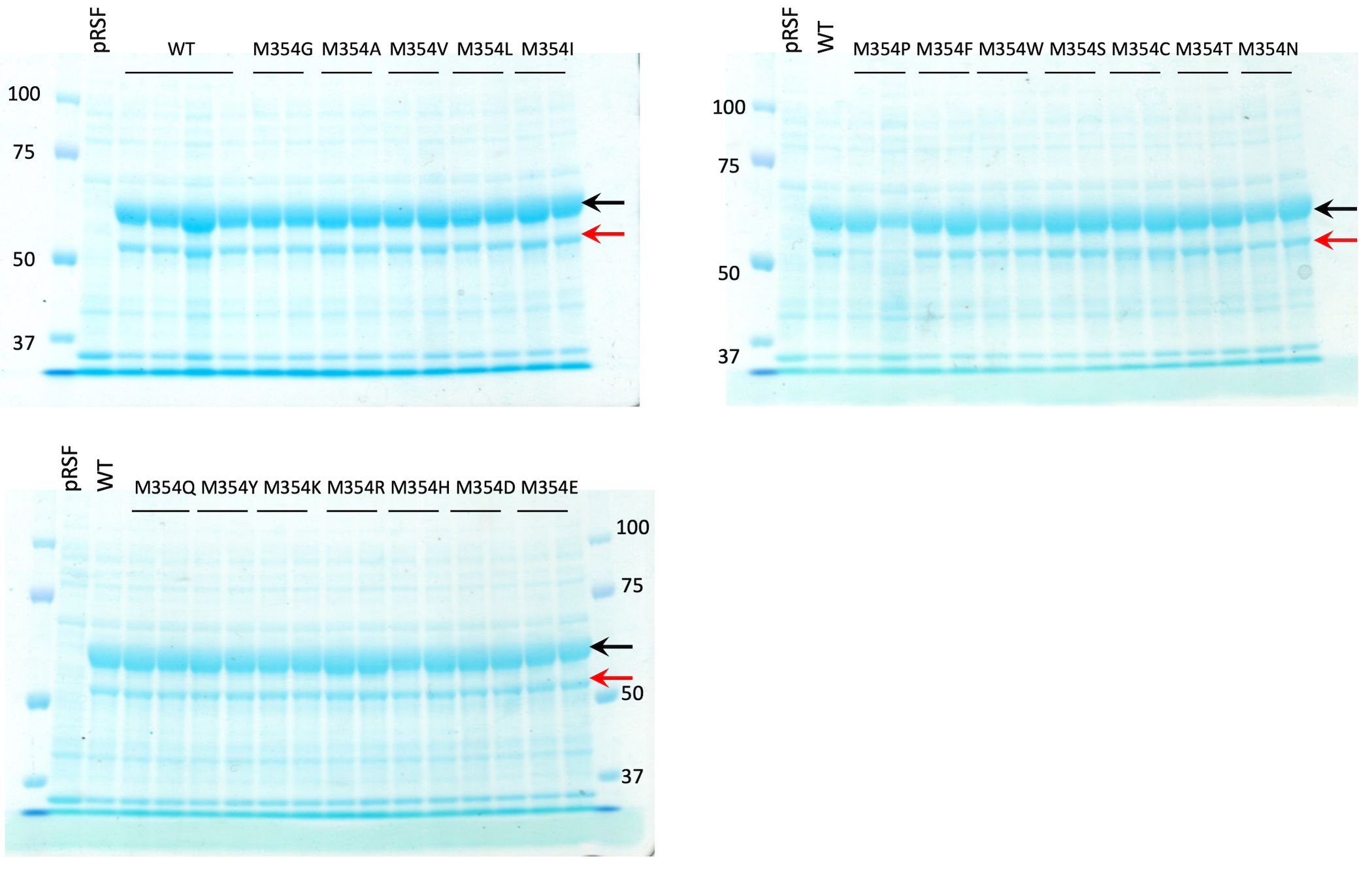


**Supplementary Fig. 14.** SDS-PAGE analysis of *E. coli* expressed CYP102A1 M354 mutants for (*S*)-(-)-limonene (**1**) and *p*-cymene (**16**) conversion. The expected positions of BMP and BMR are indicated by red and black arrows respectively. Lane pRSF: empty vector. Lane WT; wild type protein.


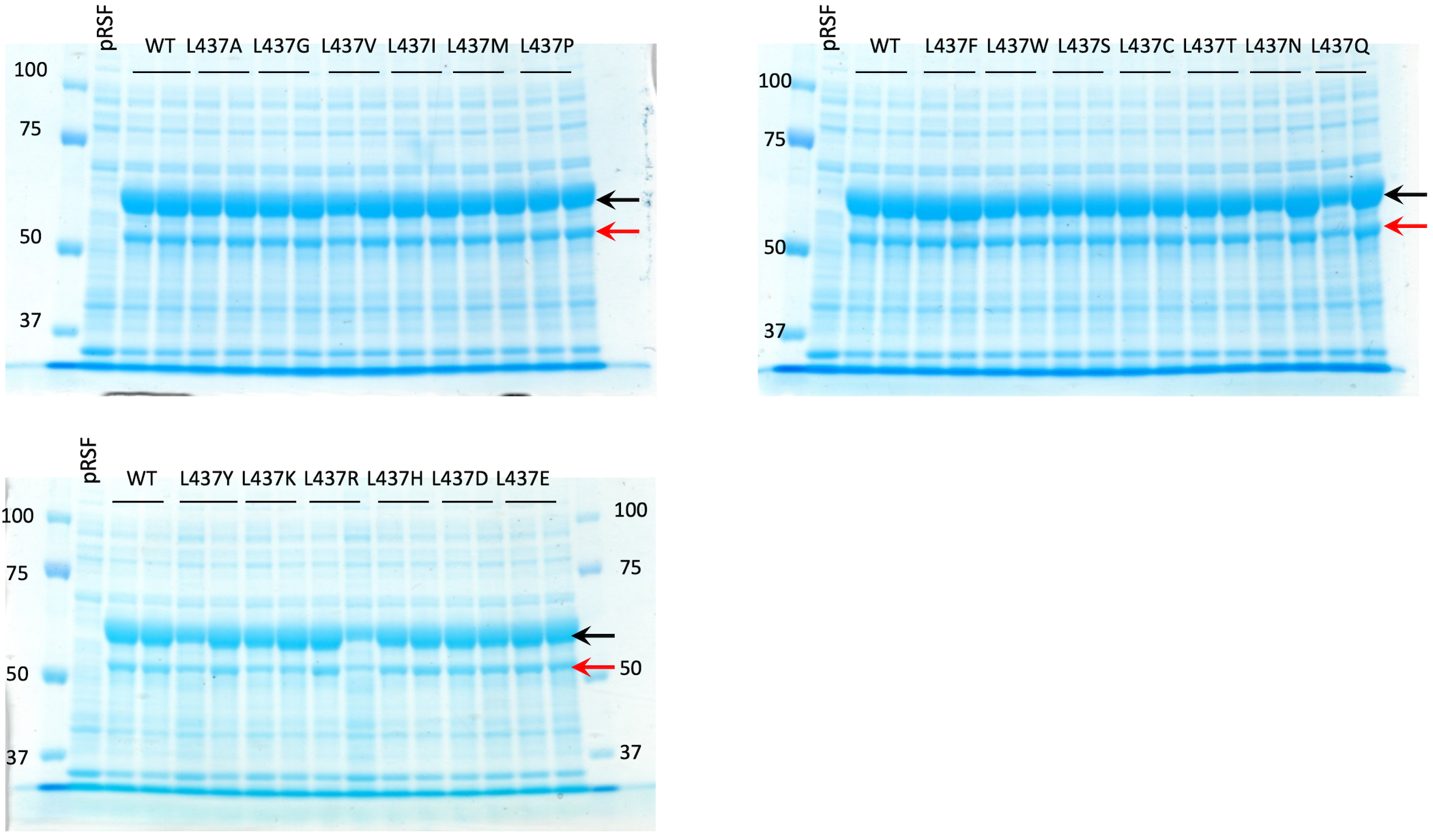


**Supplementary Fig. 15.** SDS-PAGE analysis of *E. coli* expressed CYP102A1 L437 mutants for (*S*)-(-)-limonene (**1**) and *p*-cymene (**16**) conversion. The expected positions of BMP and BMR are indicated by red and black arrows respectively. Lane pRSF: empty vector. Lane WT; wild type protein.

**
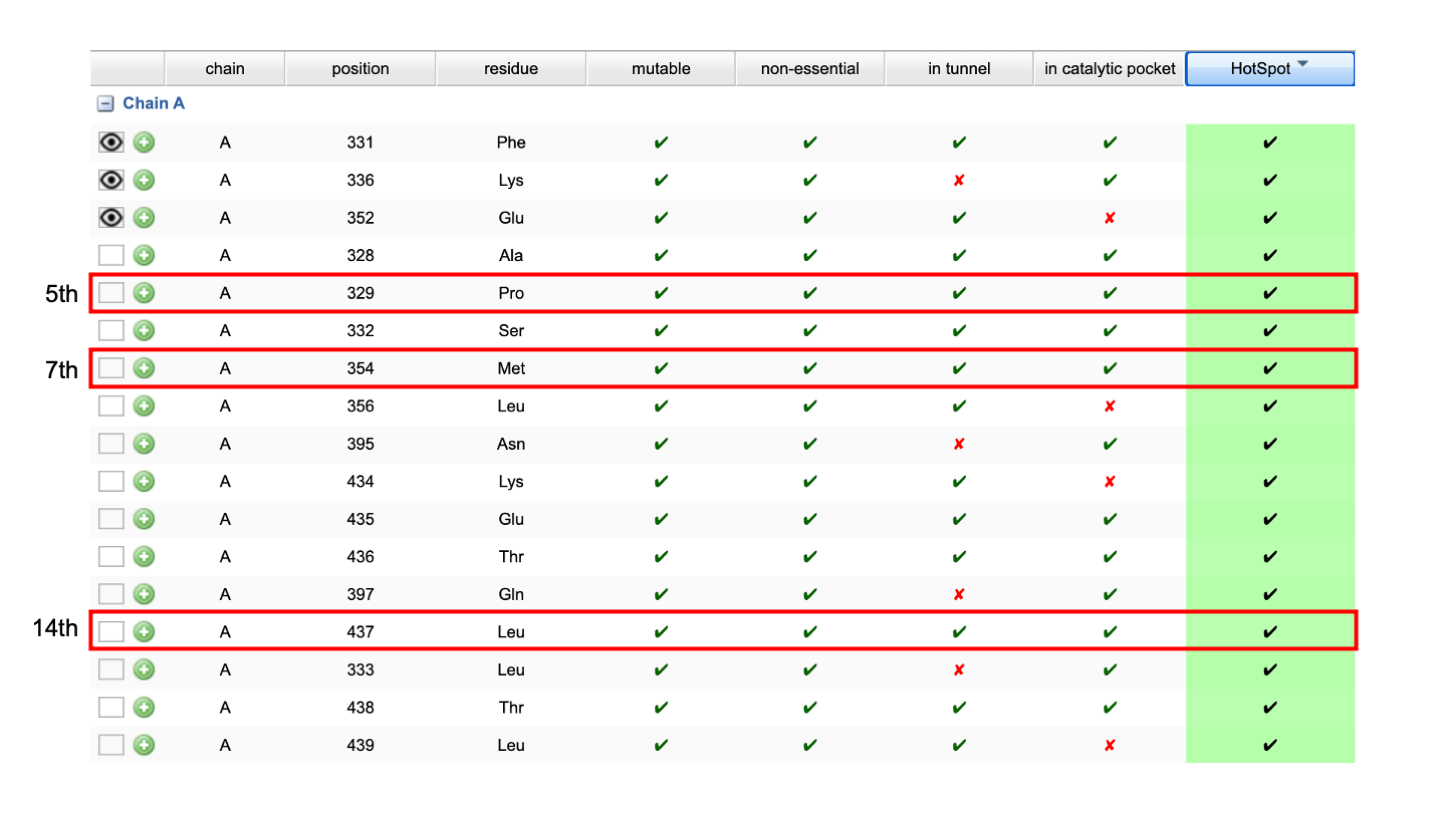
**

**Supplementary Fig. 16**. The ranked list predicted by HowSpot Wizard for CYP102A1.

Output screen of the ranked list of functional hotspots predicted by the HotSpot Wizard web server from the CYP102A1 (PDB ID: 1BU7) input structure. The 17 residues predicted as hotspots are ranked, and the other ones are removed from the list. The red boxes represent three of the six effective mutation sites (P329 at 5th, M354 at 7th, and L437 at 14th in the HotSpot Wizard list) that enhance regioselectivity predicted by MSPER. The remaining validated mutation sites (A74, L75, and A330) were not predicted as hotspots by HotSpot wizard.

**
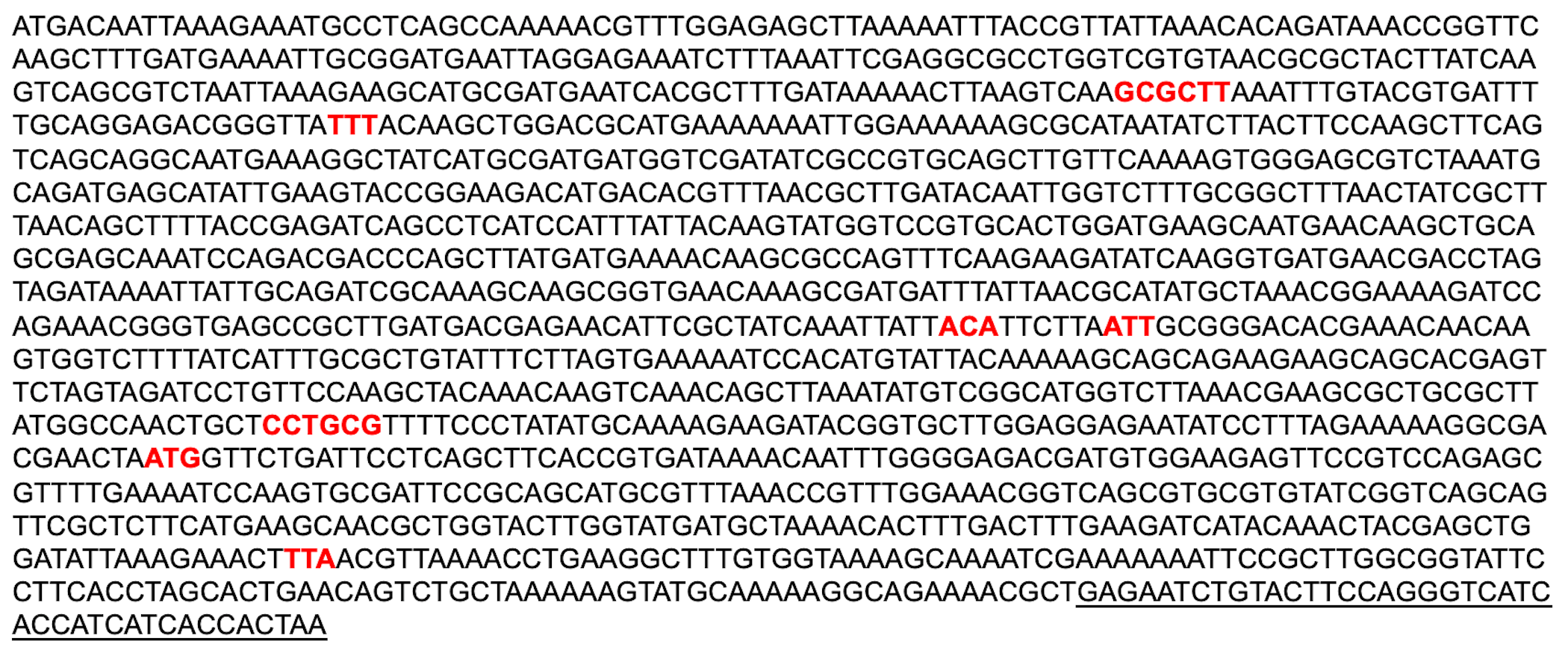
**

**Supplementary Fig. 17.** The sequence of BMPtevHis.

The underlined sequences show the positions of the TEV cleavage site (GAGAATCTGTACTTCCAGGGT) and His6-tag (CATCACCATCATCACCACTAA). The nine mutated positions (A74 GCG, L75 CTT, F87 TTT, T260 ACA, I263 ATT, P329 CCT, A330 GCG, M354 ATG, L437 TTA) are highlighted in red and bolded.

**
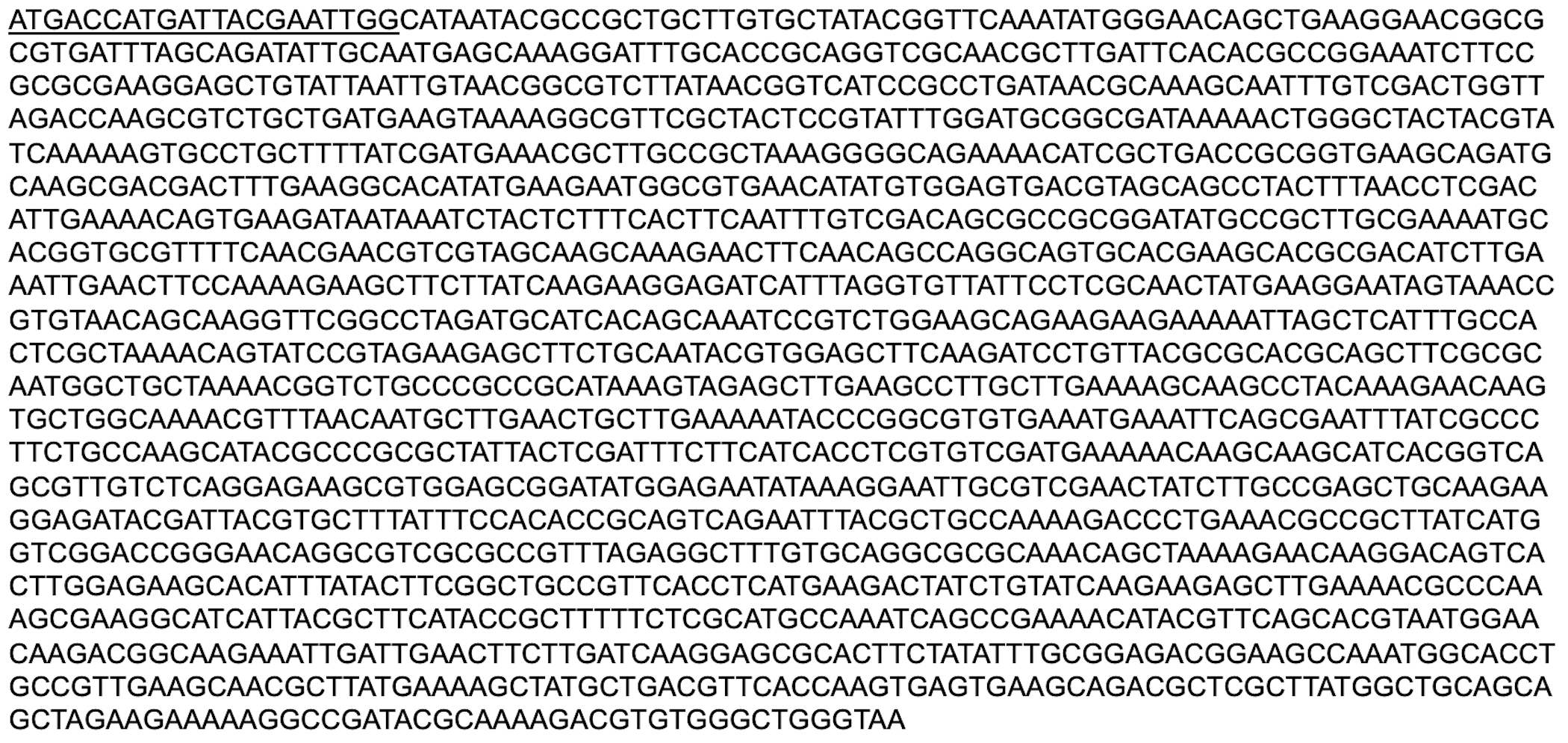
**

**Supplementary Fig. 18**. The sequence of BMR.

The underlined sequences show an additional *lacZ* sequence derived from the pSTV28 vector.

**Supplementary Table 1.** Distribution of products of the oxidation of (*S*)-(-)*-*limonene (**1**) by CYP102A1 and the proposed mutants determined by MD simulation.


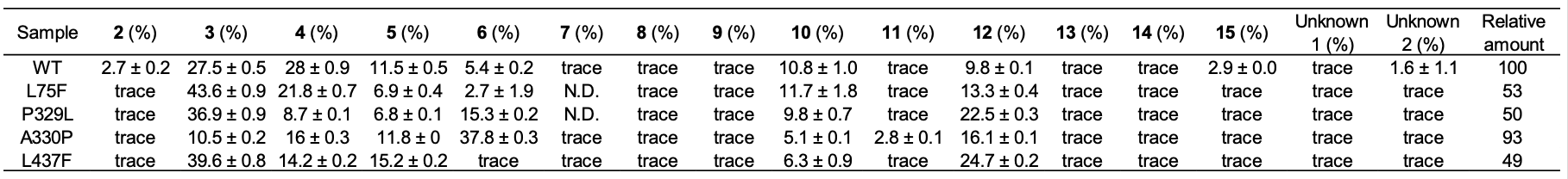


The percentage of derivatives and the relative amount of total reaction products were calculated from the peak area of the total ion chromatogram. The data are presented as the averages of three independent experiments and the standard deviation.

**Supplementary Table 2.** Substitution candidate residue scores (*S*_scr_s) and the rankings for *trans*-carveol (**12**). Note that residues with a *S*_scr_ = 0 and C400, the enzyme’s active site, were removed from the ranking.


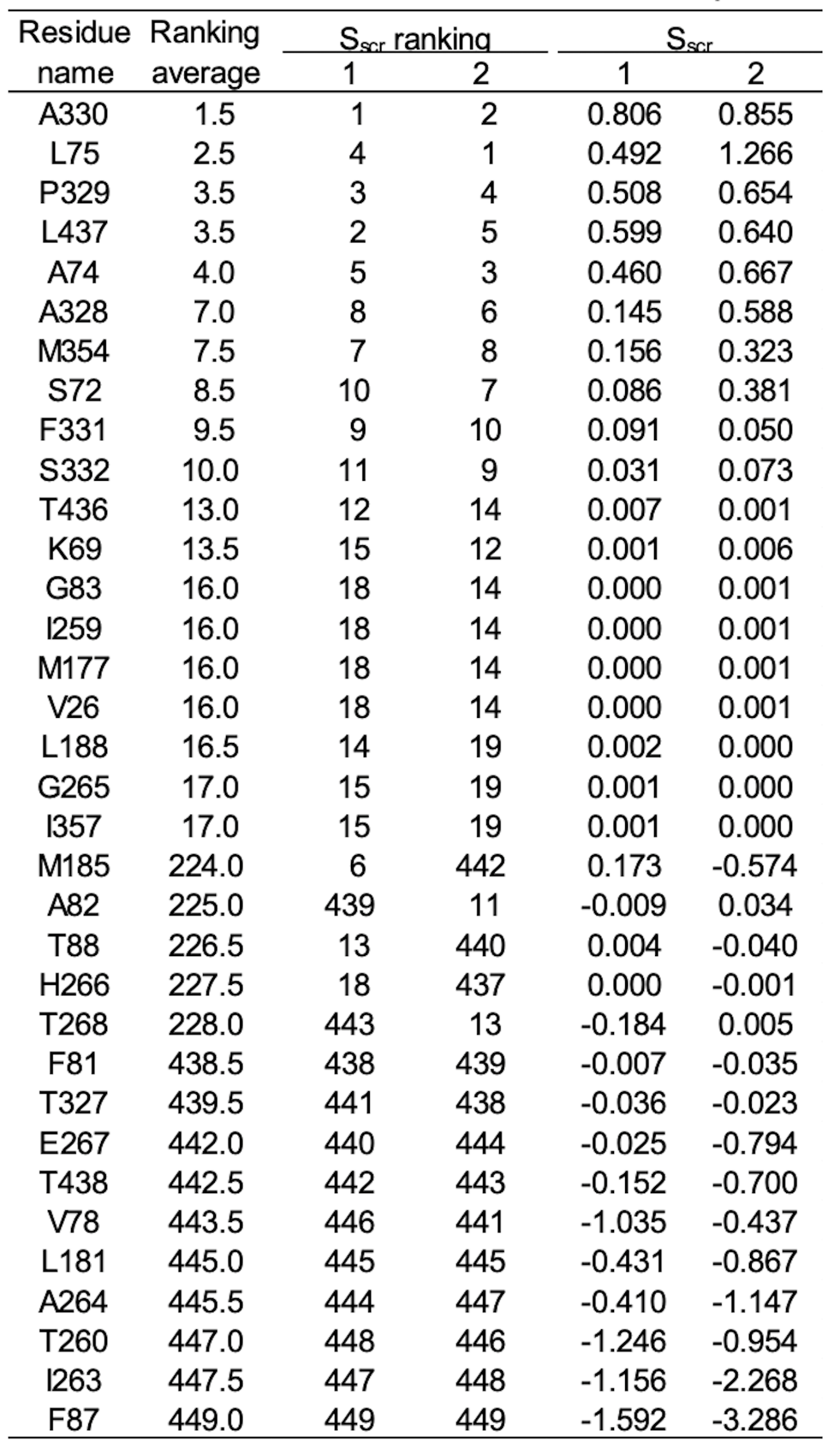


**Supplementary Table 3.** S_scr_s and the rankings for *cis*-isopiperitenol (**6**).


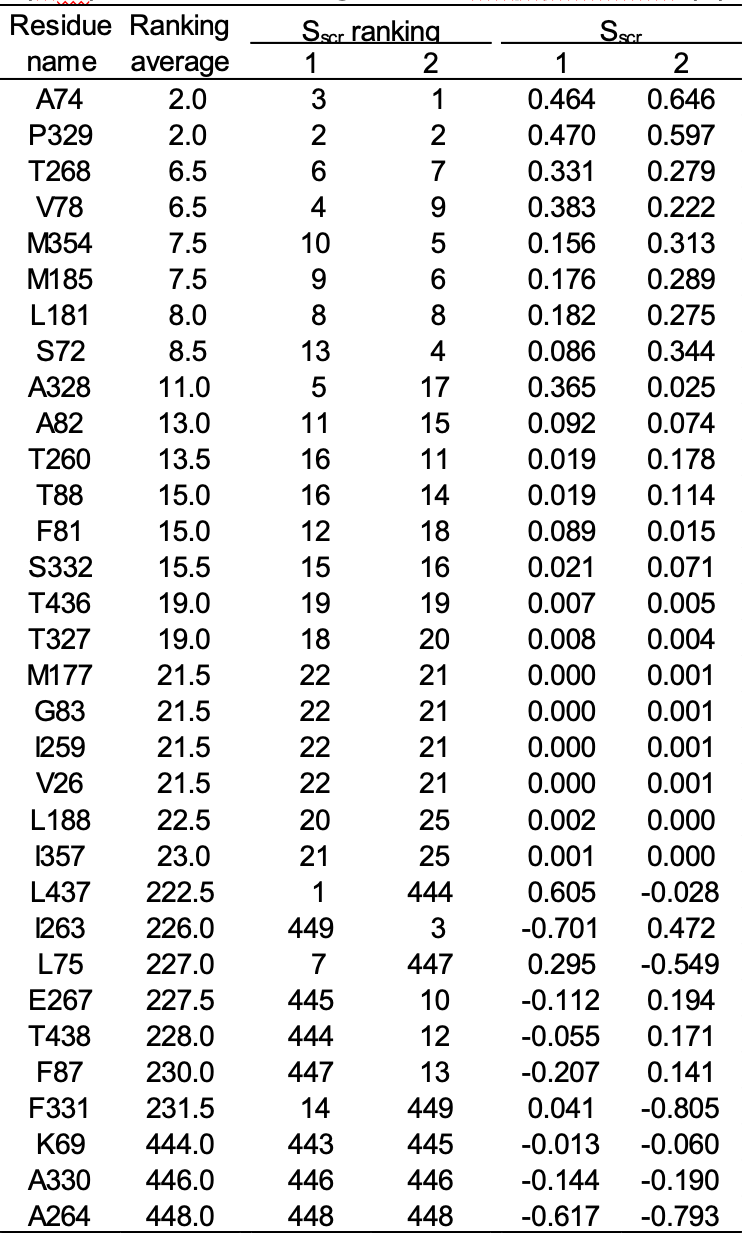


**Supplementary Table 4.** S_scr_s and the rankings for carvacrol (**22**).


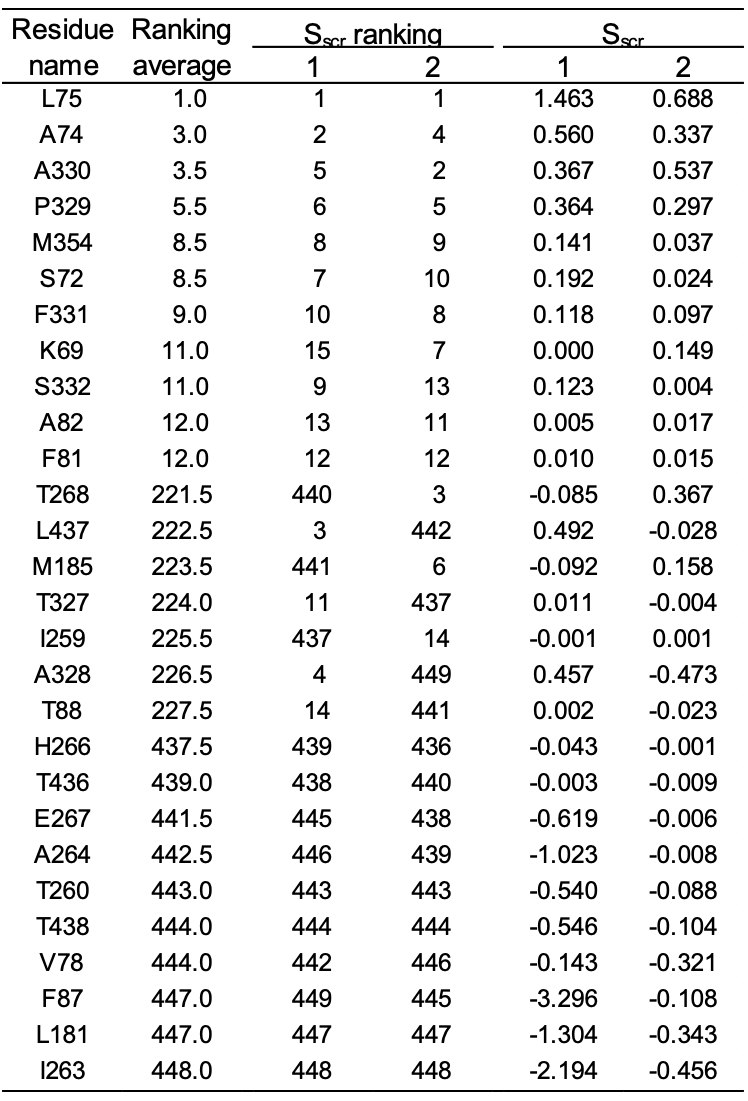


**Supplementary Table 5.** S_scr_s and the rankings for thymol (**21**).


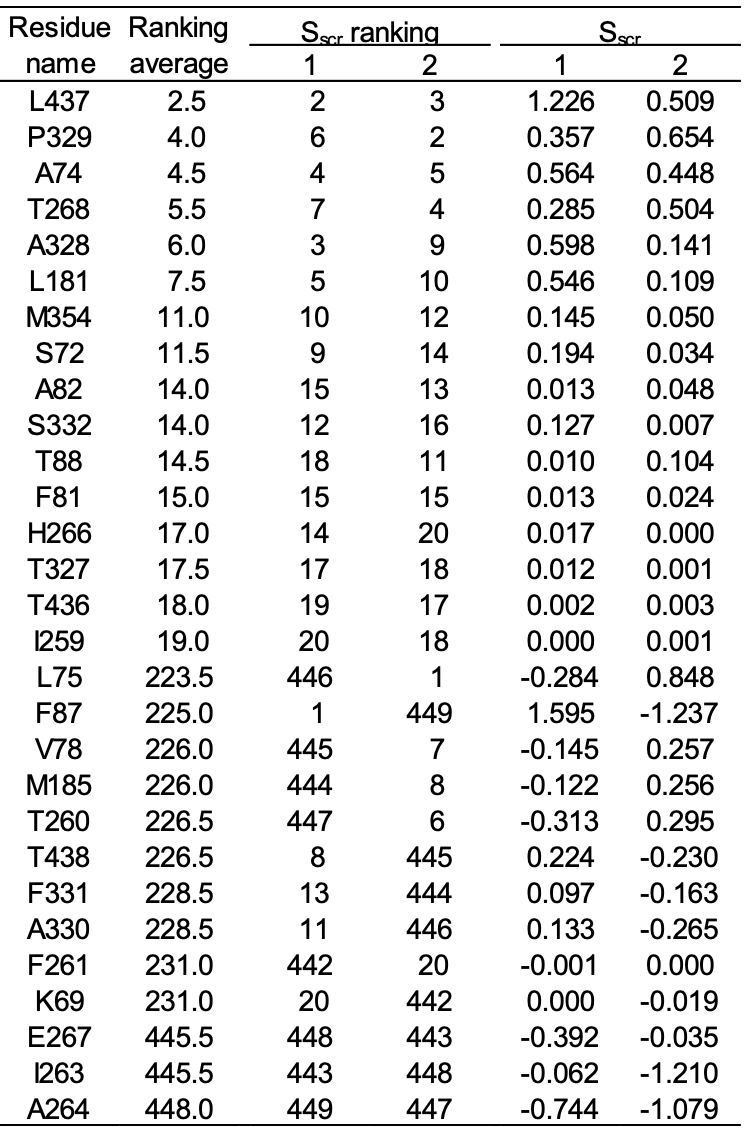


**Supplementary Table 6.** Distribution of products of the oxidation of (*S*)-(-)*-*limonene (**1**) by A74 mutants.


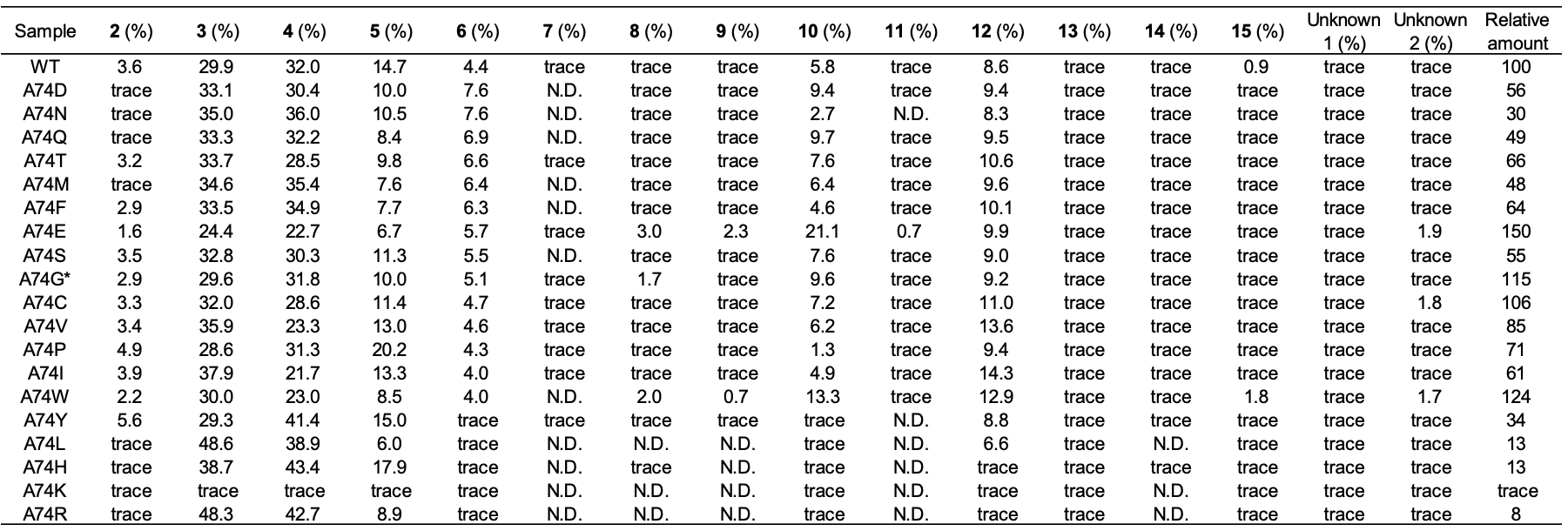


The percentage of derivatives and the relative amount of total reaction products were calculated from the peak area of the total ion chromatogram. The data for mutants with an asterisk are presented as the percentage calculated in one experiment, and the other data are presented as the averages of two independent experiments.

**Supplementary Table 7.** Distribution of products of the oxidation of (*S*)-(-)*-*limonene (**1**) by L75 mutants.


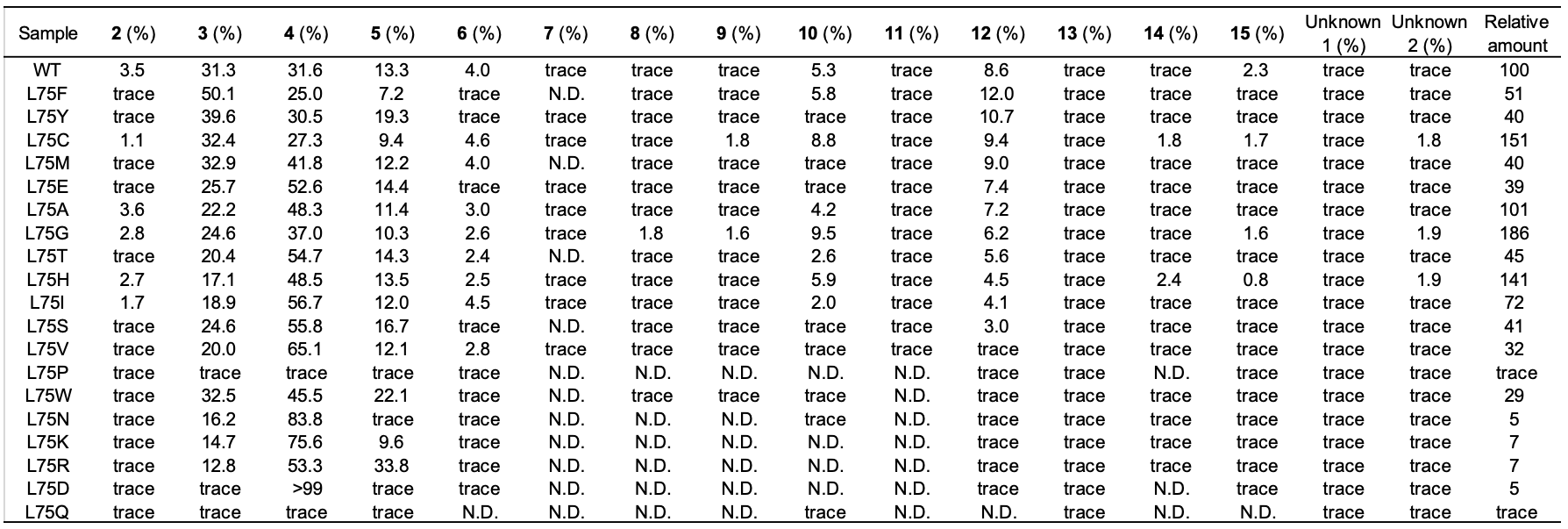


The percentage of derivatives and the relative amount of total reaction products were calculated from the peak area of the total ion chromatogram. The data are presented as the averages of two independent experiments.

**Supplementary Table 8.** Distribution of products of the oxidation of (*S*)-(-)*-*limonene (**1**) by F87 mutants.


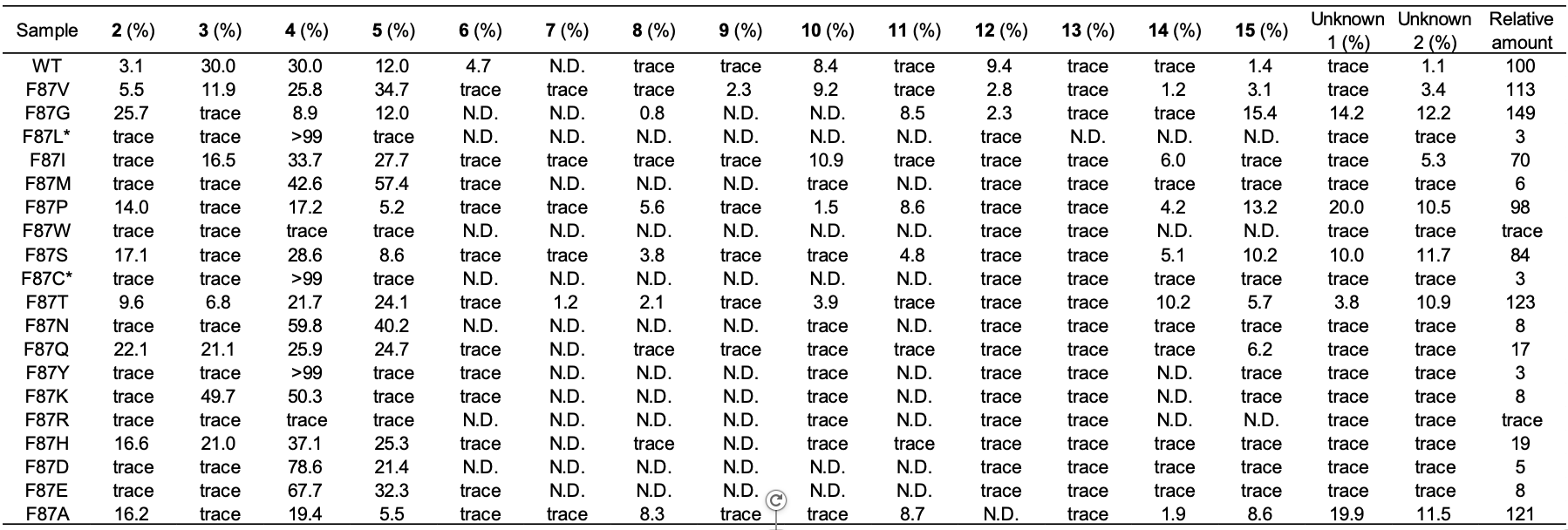


The percentage of derivatives and the relative amount of total reaction products were calculated from the peak area of the total ion chromatogram. The data for mutants with an asterisk are presented as the percentage calculated from one experiment, and the other data are presented as the averages of two independent experiments.

**Supplementary Table 9.** Distribution of products of the oxidation of (*S*)-(-)*-*limonene (**1**) by T260 mutants.


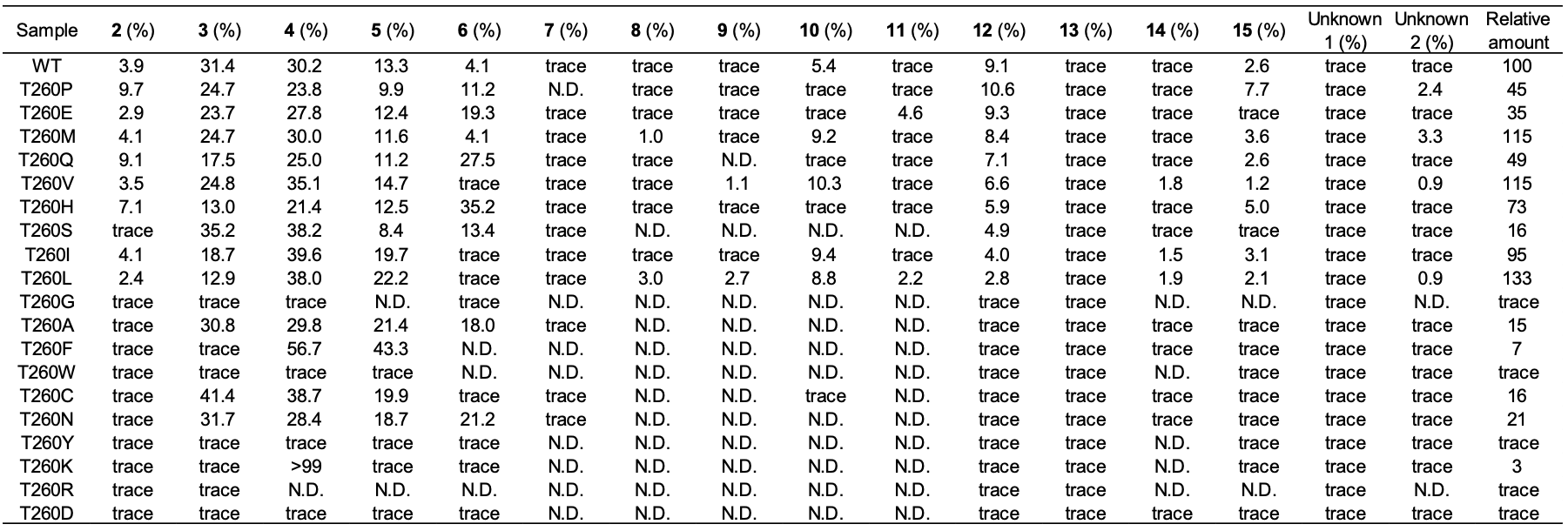


The percentage of derivatives and the relative amount of total reaction products were calculated from the peak area of the total ion chromatogram. The data are presented as the averages of two independent experiments.

**Supplementary Table 10.** Distribution of products of the oxidation of (*S*)-(-)*-*limonene (**1**) by I263 mutants.


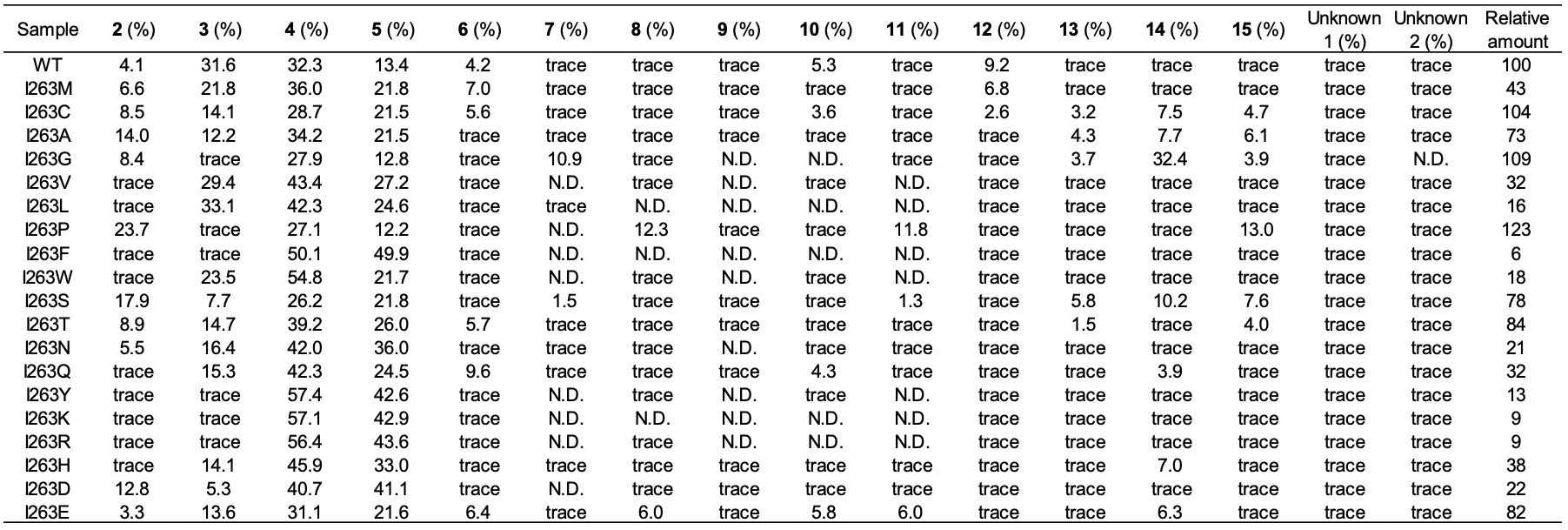


The percentage of derivatives and the relative amount of total reaction products were calculated from the peak area of the total ion chromatogram. The data are presented as the averages of two independent experiments.

**Supplementary Table 11.** Distribution of products of the oxidation of (*S*)-(-)*-*limonene (**1**) by P329 mutants.


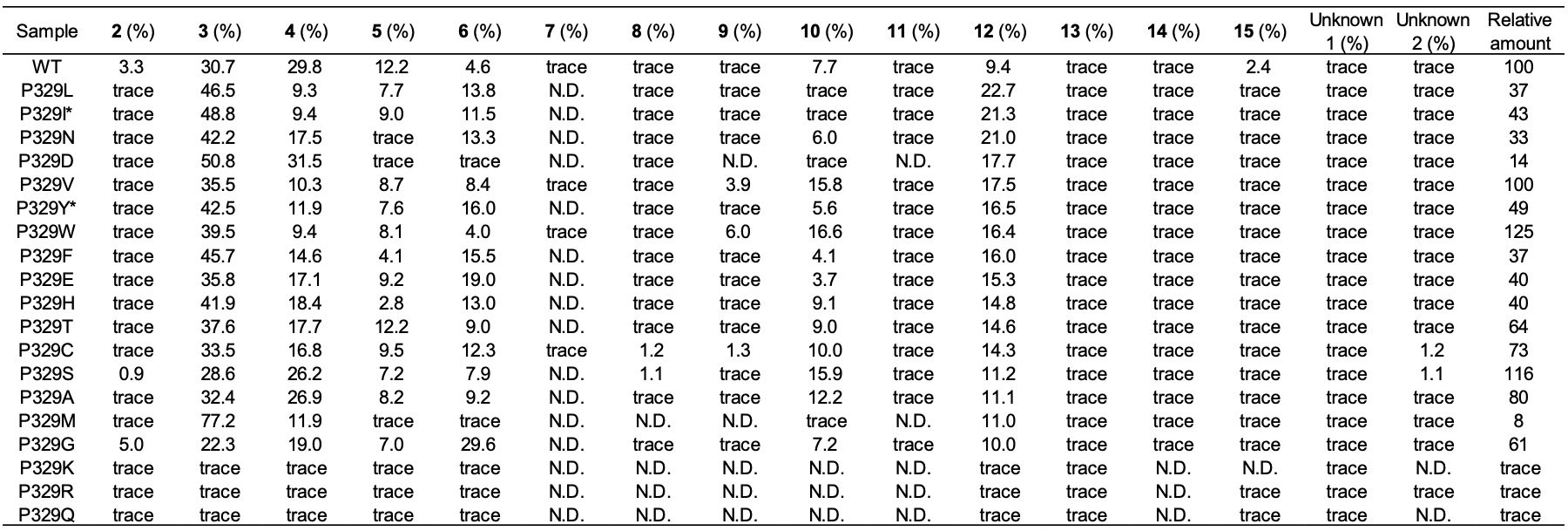


The percentage of derivatives and the relative amount of total reaction products were calculated from the peak area of the total ion chromatogram. The data for mutants with an asterisk are presented as the percentage calculated from one experiment, and the other data are presented as the averages of two independent experiments.

**Supplementary Table 12.** Distribution of products of the oxidation of (*S*)-(-)*-*limonene (**1**) by A330 mutants.


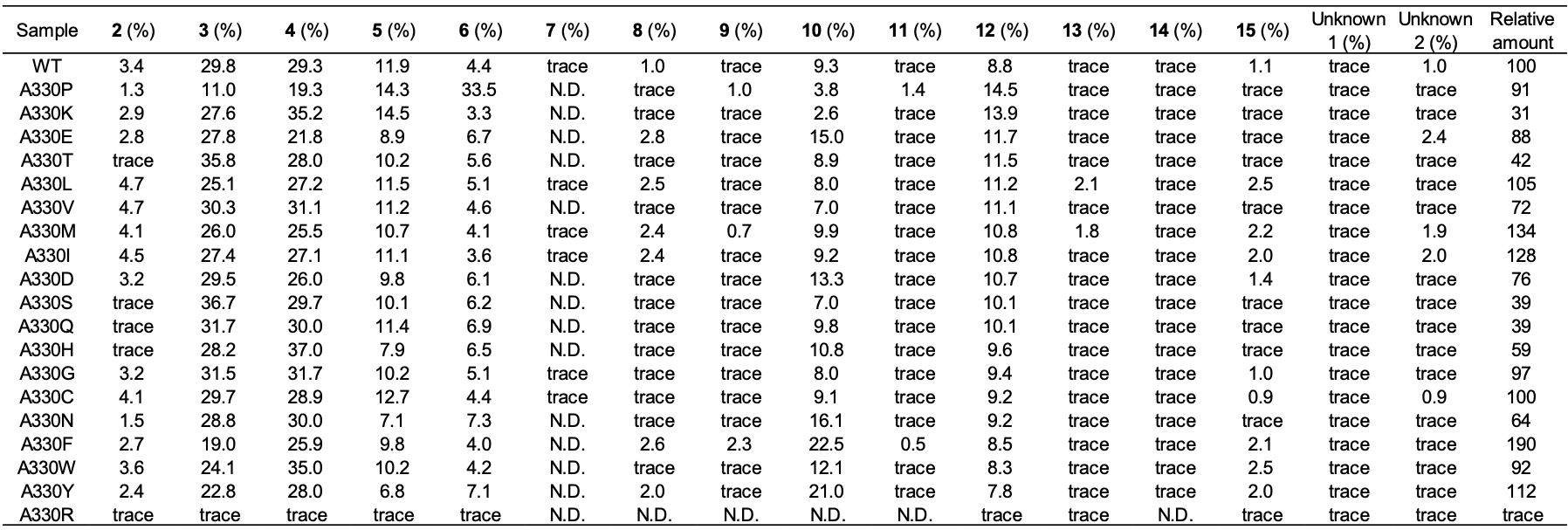


The percentage of derivatives and the relative amount of total reaction products were calculated from the peak area of the total ion chromatogram. The data are presented as the averages of two independent experiments.

**Supplementary Table 13.** Distribution of products of the oxidation of (*S*)-(-)*-*limonene (**1**) by L437 mutants.


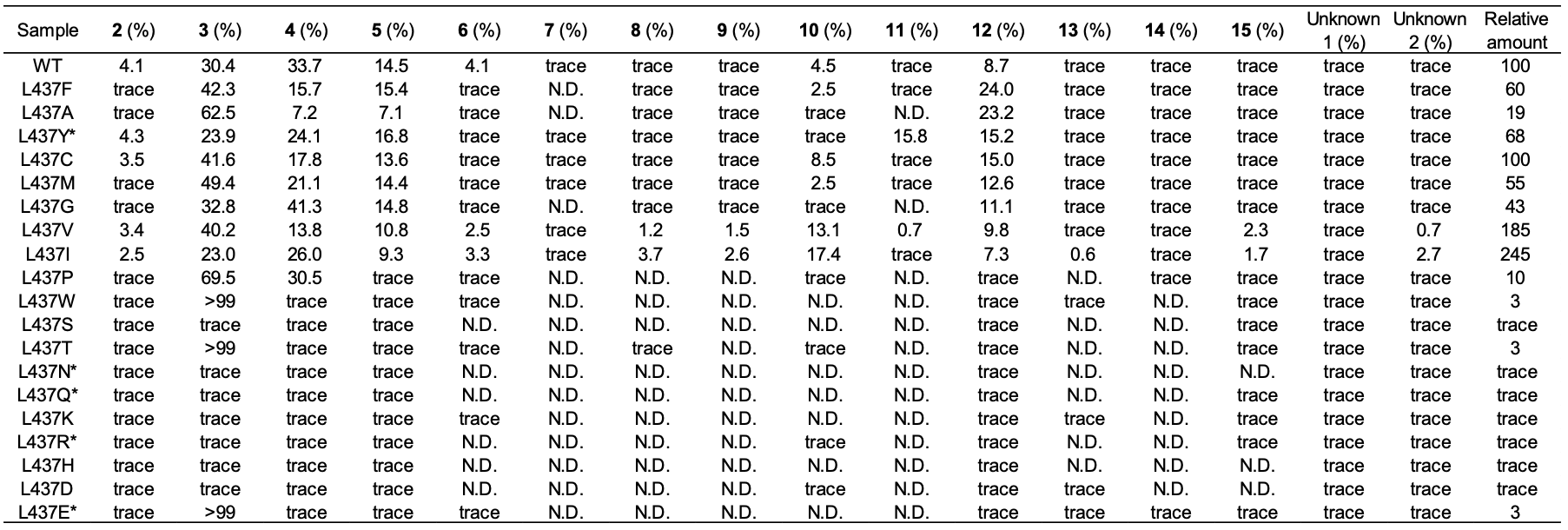


The percentage of derivatives and the relative amount of total reaction products were calculated from the peak area of the total ion chromatogram. The data for mutants with an asterisk are presented as the percentage calculated from one experiment, and the other data are presented as the averages of two independent experiments.

**Supplementary Table 14.** Distribution of products of the oxidation of *p*-cymene (**16**) by A74 mutants.


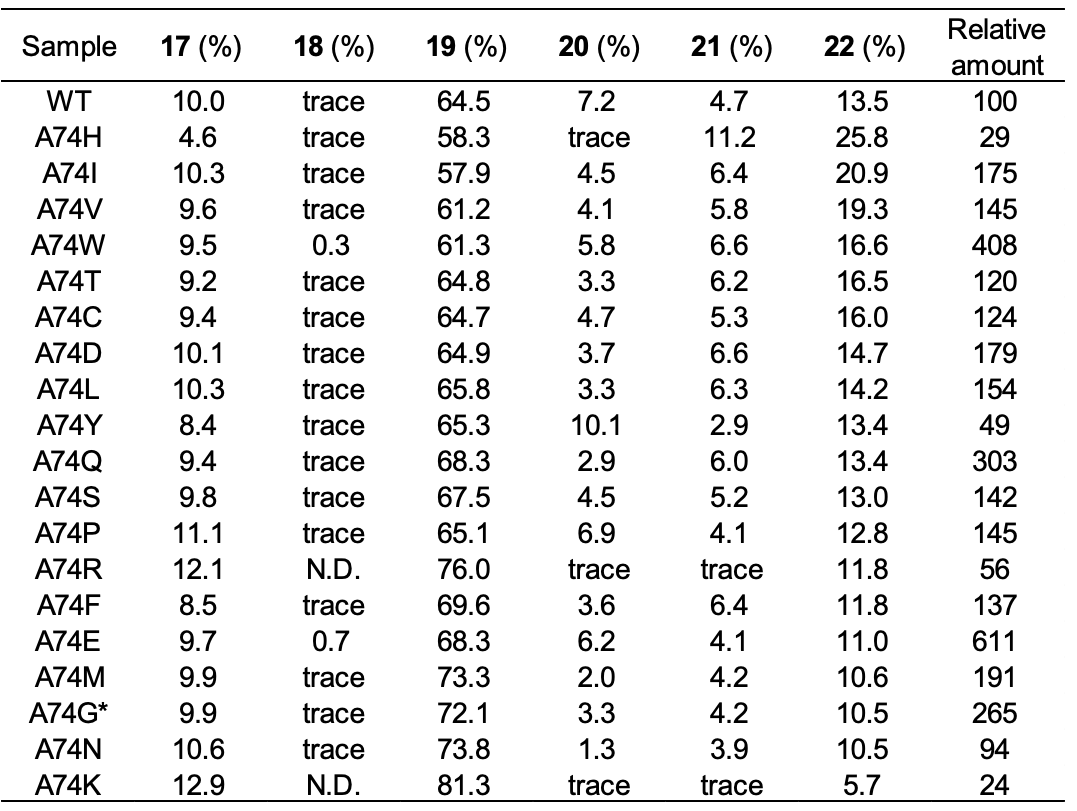


The percentage of derivatives and the relative amount of total reaction products were calculated from the peak area of the total ion chromatogram. The data for mutants with an asterisk are presented as the percentage calculated from one experiment, and the other data are presented as the averages of two independent experiments.

**Supplementary Table 15.** Distribution of products of the oxidation of *p*-cymene (**16**) by L75 mutants.


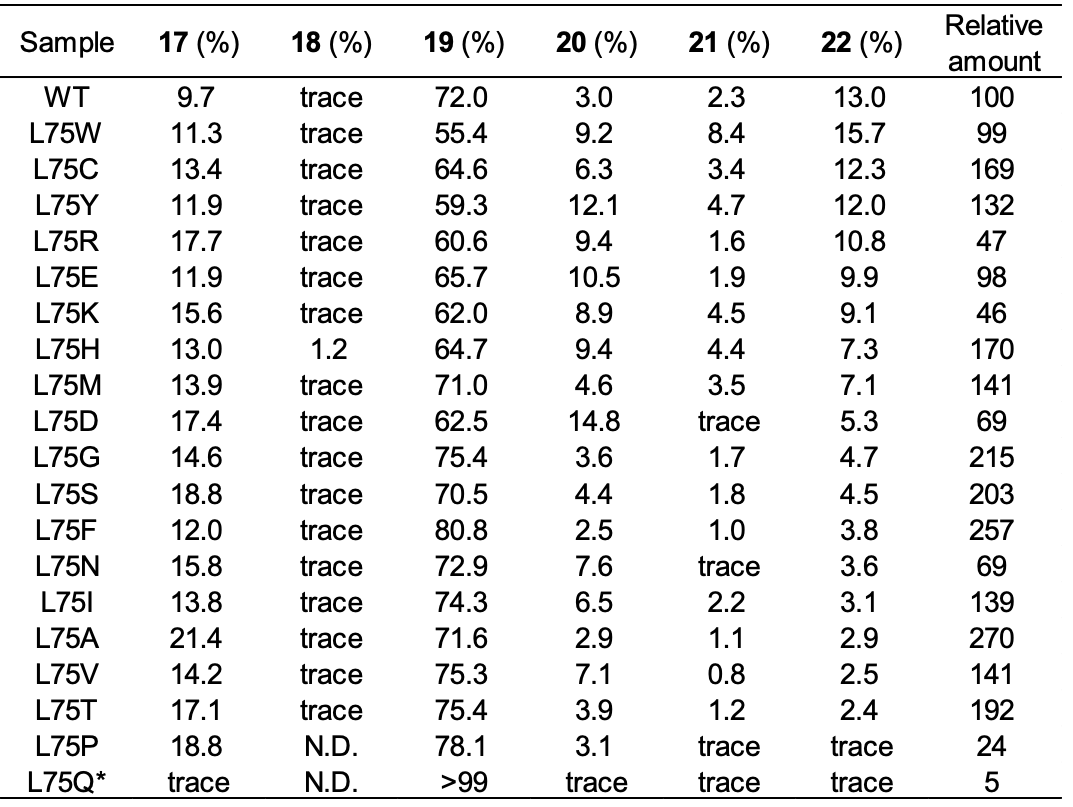


The percentage of derivatives and the relative amount of total reaction products were calculated from the peak area of the total ion chromatogram. The data for mutants with an asterisk are presented as the percentage calculated from one experiment, and the other data are presented as the averages of two independent experiments.

**Supplementary Table 16.** Distribution of products of the oxidation of *p*-cymene (**16**) by I263 mutants.


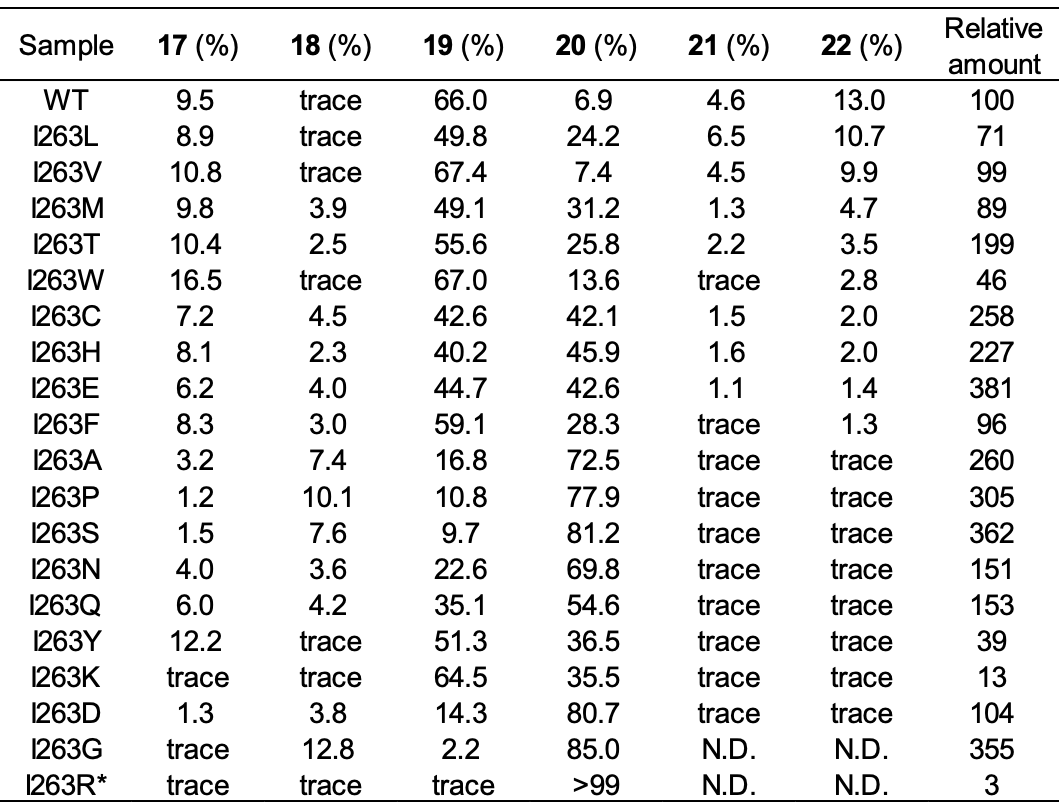


The percentage of derivatives and the relative amount of total reaction products were calculated from the peak area of the total ion chromatogram. The data for mutants with an asterisk are presented as the percentage calculated from one experiment, and the other data are presented as the averages of two independent experiments.

**Supplementary Table 17.** Distribution of products of the oxidation of *p*-cymene (**16**) by P329 mutants.


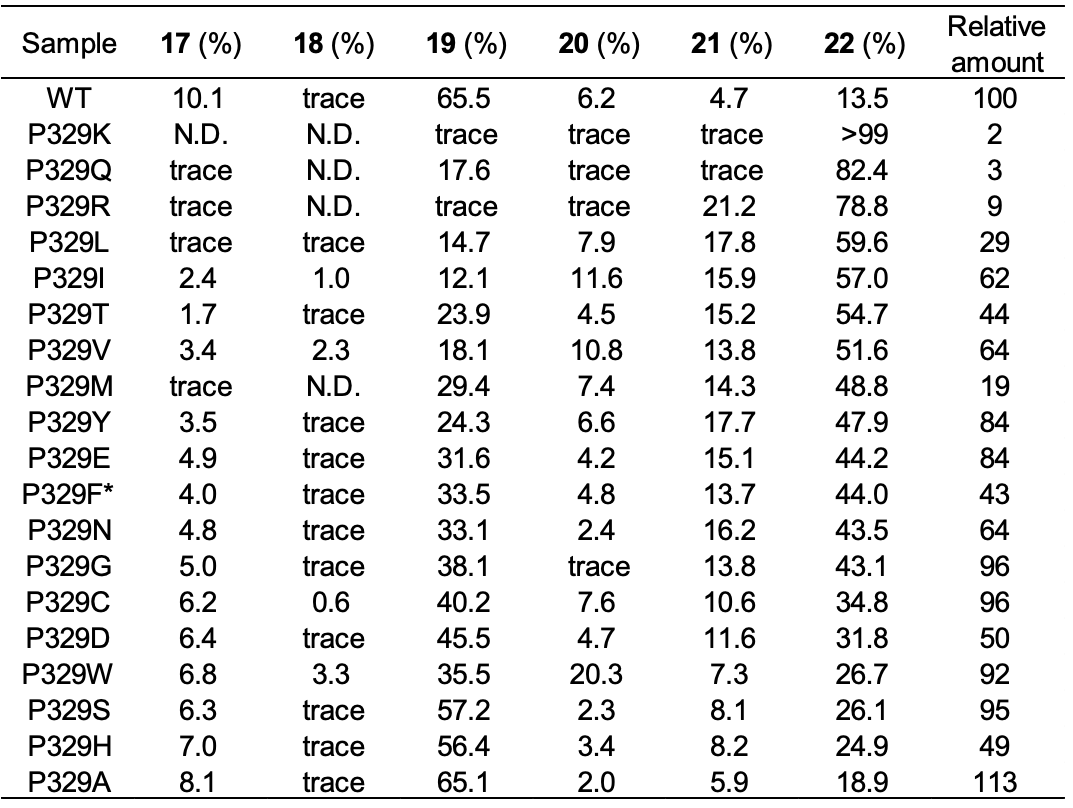


The percentage of derivatives and the relative amount of total reaction products were calculated from the peak area of the total ion chromatogram. The data for mutants with an asterisk are presented as the percentage calculated from one experiment, and the other data are presented as the averages of two independent experiments.

**Supplementary Table 18.** Distribution of products of the oxidation of *p*-cymene (**16**) by A330 mutants.


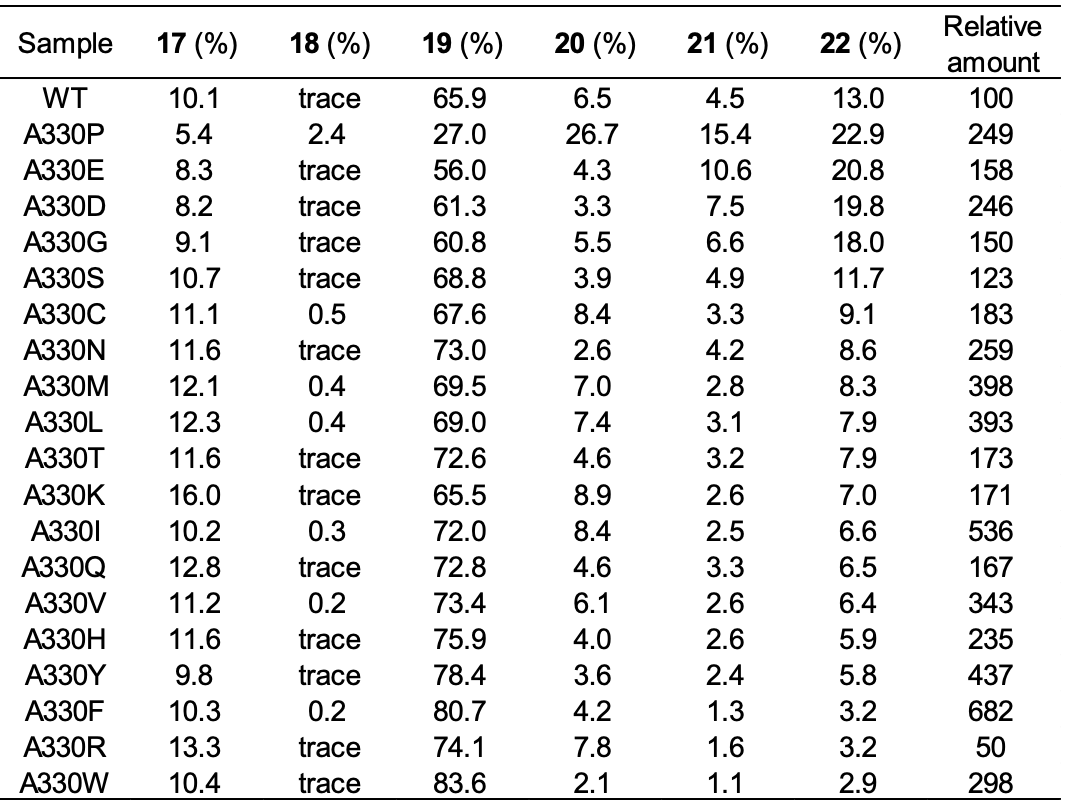


The percentage of derivatives and the relative amount of total reaction products were calculated from the peak area of the total ion chromatogram. The data are presented as the averages of two independent experiments.

**Supplementary Table 19.** Distribution of products of the oxidation of *p*-cymene (**16**) by M354 mutants.


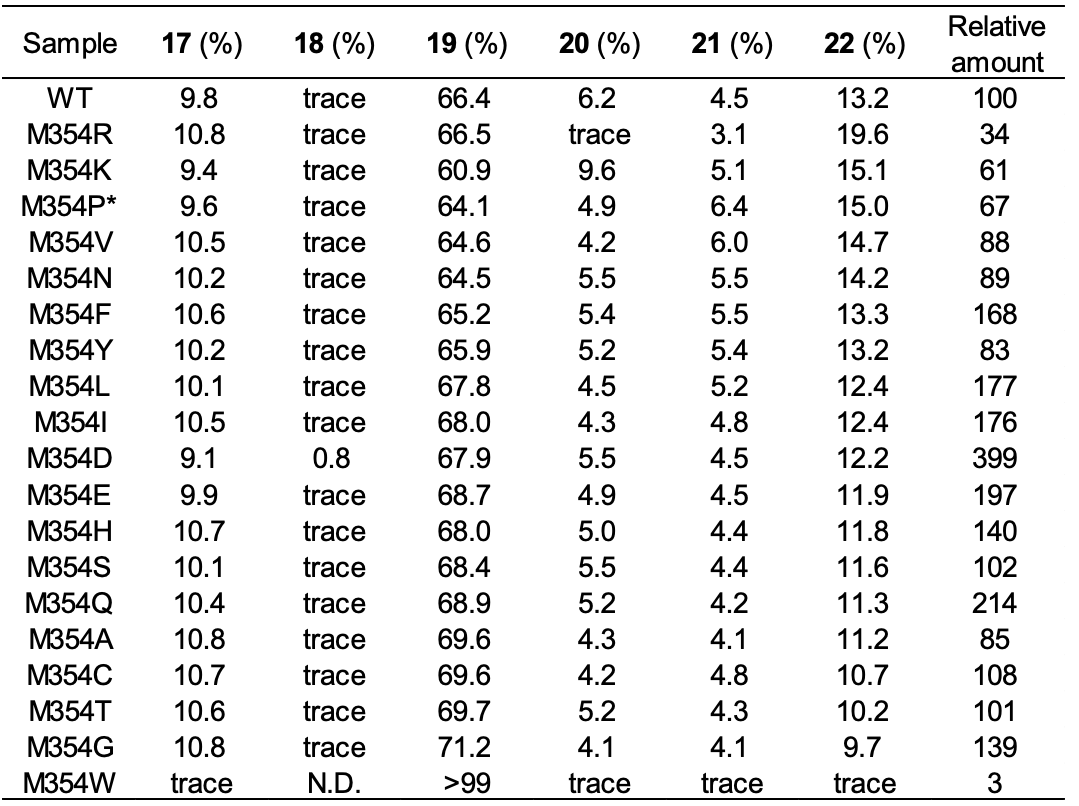


The percentage of derivatives and the relative amount of total reaction products were calculated from the peak area of the total ion chromatogram. The data for mutants with an asterisk are presented as the percentage calculated from one experiment, and the other data are presented as the averages of two independent experiments.

**Supplementary Table 20.** Distribution of products of the oxidation of *p*-cymene (**16**) by L437 mutants.


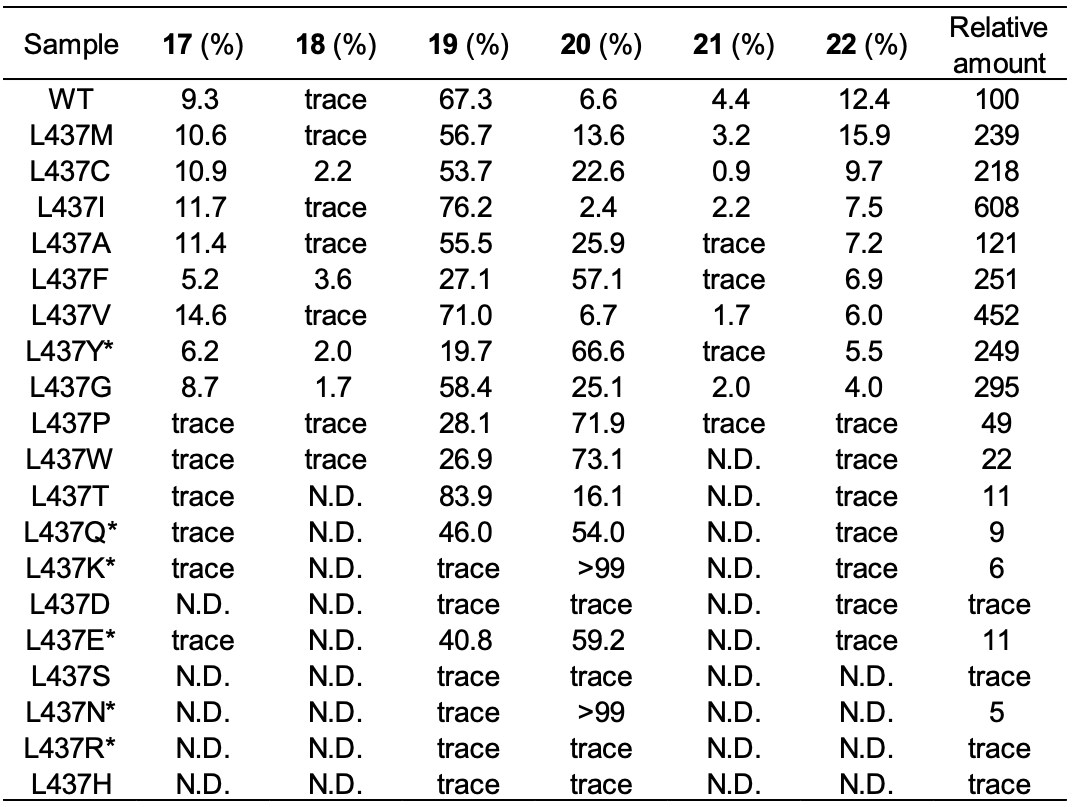


The percentage of derivatives and the relative amount of total reaction products were calculated from the peak area of the total ion chromatogram. The data for mutants with an asterisk are presented as the percentage calculated from one experiment, and the other data are presented as the averages of two independent experiments.

**Supplementary Table 21.** Primers used for PCR.


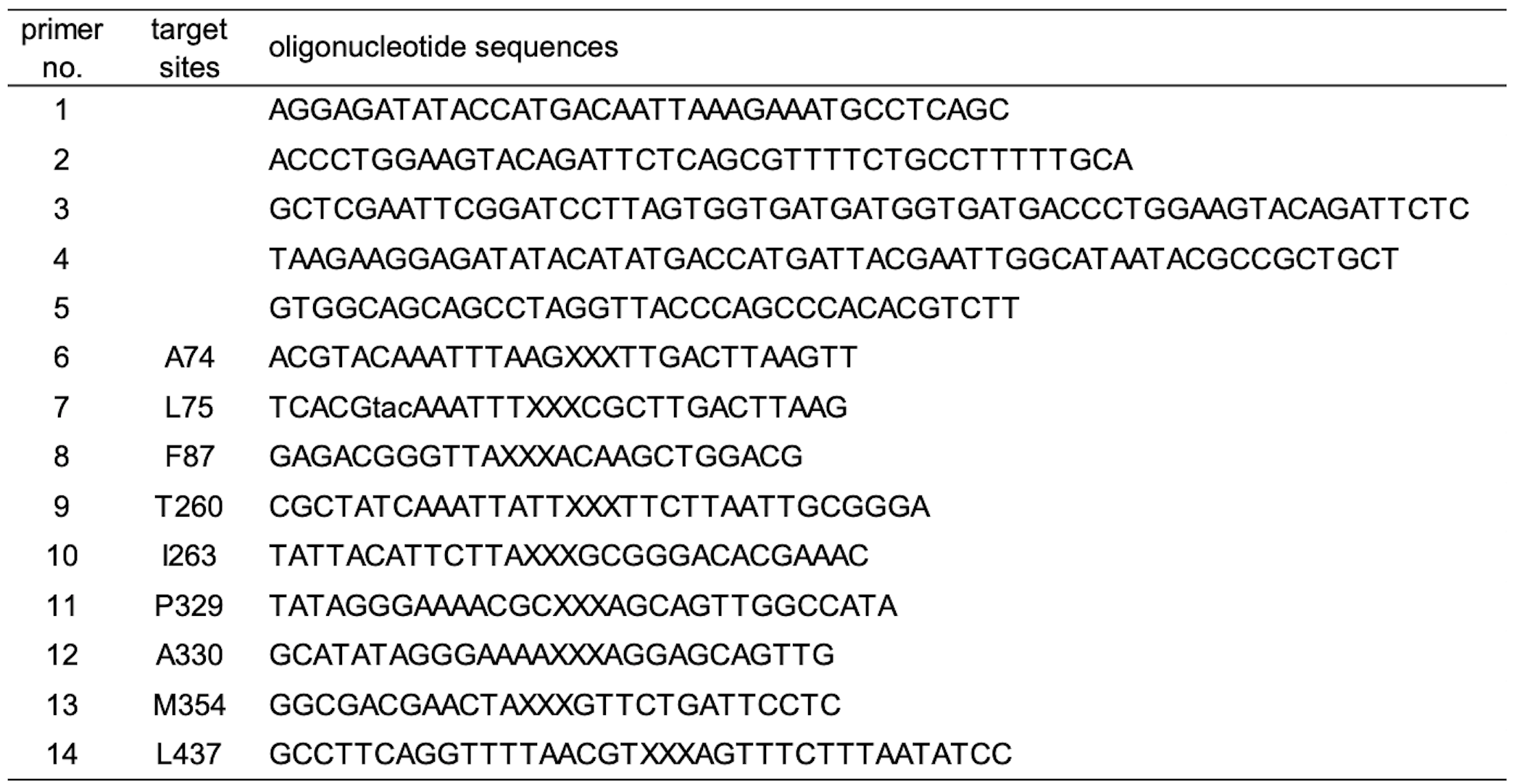


XXX represents codons used for substitutions (Gly: ggt; Ala: gca; Val: gtt; Leu: ctg; Ile: att; Met: atg; Pro: ccg; Phe: ttc; Trp: tgg; Ser: agc; Cys: tgt; Thr: acc; Asn: aat; Gln: cag; Tyr: tat; Lys: aaa; Arg: cgt; His: cat; Asp: gat; Glu: gaa).

**Supplementary Table 22.** Substitution candidate residue scores (*S*_scr_s) and the ranking for *trans*-carveol (**12**) by using ZDOCK. Note that residues with a *S*_scr_ = 0 and C400, the active site of the enzyme, were removed from the ranking.


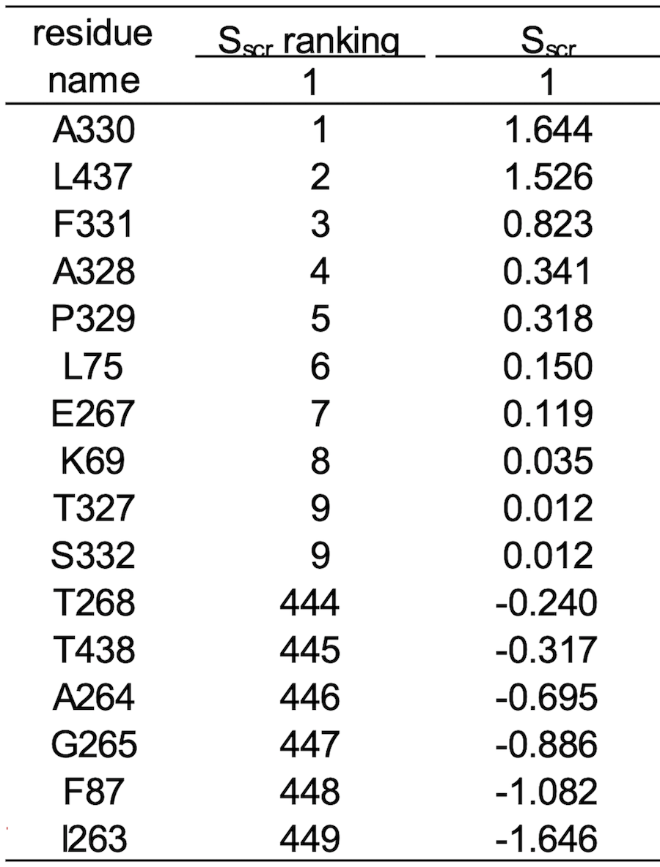


**Supplementary data 1:** RESP charge parameters for (*S*)-(-)*-*limonene

Information regarding the charge of compound atoms.

The charges of the compounds modeled using quantum chemical calculations are described below in Tripos mol2 format.

S-limonene:

@<TRIPOS>MOLECULE

LIM

26 26 1 0 0

SMALL

No charge or current charge

@<TRIPOS>ATOM

1 C1 3.5400 1.4200 0.0000 c3 1 LIM -0.075833

2 H1 3.2140 2.3060 0.5360 hc 1 LIM 0.064191

3 H2 3.2110 1.5570 -1.0290 hc 1 LIM 0.064191

4 C2 5.0470 1.3370 0.0320 c2 1 LIM -0.357134

5 H3 5.5770 2.2590 -0.1420 ha 1 LIM 0.157137

6 C3 5.7350 0.2300 0.2590 c2 1 LIM 0.194138

7 C4 7.2410 0.2110 0.3130 c3 1 LIM -0.432983

8 H4 7.5890 -0.1750 1.2680 hc 1 LIM 0.115486

9 H5 7.6500 -0.4390 -0.4580 hc 1 LIM 0.115486

10 H6 7.6600 1.2010 0.1770 hc 1 LIM 0.115486

11 C5 5.0470 -1.0970 0.4950 c3 1 LIM -0.034494

12 H7 5.5710 -1.8760 -0.0550 hc 1 LIM 0.038035

13 H8 5.1420 -1.3630 1.5470 hc 1 LIM 0.038035

14 C6 3.5730 -1.0820 0.0890 c3 1 LIM -0.237746

15 H9 3.5030 -1.1090 -0.9960 hc 1 LIM 0.084636

16 H10 3.0760 -1.9730 0.4620 hc 1 LIM 0.084636

17 C7 2.8780 0.1790 0.6200 c3 1 LIM 0.093633

18 H11 3.0640 0.2220 1.6900 hc 1 LIM 0.051090

19 C8 1.3700 0.1680 0.4290 c2 1 LIM 0.081415

20 C10 0.5580 0.3920 1.4490 c2 1 LIM -0.580322

21 H15 0.9310 0.5770 2.4410 ha 1 LIM 0.200469

22 H16 -0.5110 0.3990 1.3330 ha 1 LIM 0.200469

23 C9 0.8250 -0.1080 -0.9530 c3 1 LIM -0.149124

24 H12 1.2400 0.5680 -1.6940 hc 1 LIM 0.056368

25 H13 1.0640 -1.1180 -1.2750 hc 1 LIM 0.056368

26 H14 -0.2520 -0.0010 -0.9720 hc 1 LIM 0.056368

@<TRIPOS>BOND

1 2 1 1

2 3 1 1

3 4 1 1

4 5 4 1

5 6 4 2

6 7 6 1

7 8 7 1

8 9 7 1

9 10 7 1

10 11 6 1

11 12 11 1

12 13 11 1

13 14 11 1

14 15 14 1

15 16 14 1

16 17 14 1

17 18 17 1

18 19 17 1

19 20 19 2

20 21 20 1

21 22 20 1

22 23 19 1

23 24 23 1

24 25 23 1

25 26 23 1

26 17 1 1

@<TRIPOS>SUBSTRUCTURE

1 LIM 1 TEMP 0 **** **** 0 ROOT

**Supplementary data 2:** RESP charge parameters for *p*-cymene

Information regarding the charge of compound atoms.

The charges of the compounds modeled using quantum chemical calculations are described below in Tripos mol2 format.

p-cymene

@<TRIPOS>MOLECULE
MOL
   24    24     1     0     0
SMALL
No charge or current charge


@<TRIPOS>ATOM
      1 C3           3.5400     1.4200     0.0000 ca         1 MOL      -0.204480
      2 H2           3.9690     2.4080     0.0000 ha         1 MOL       0.143639
      3 C1           2.1580     1.2860    -0.0000 ca         1 MOL      -0.241703
      4 H1           1.5450     2.1710     0.0000 ha         1 MOL       0.155496
      5 C2           1.5600     0.0370     0.0000 ca         1 MOL       0.238970
      6 C8           0.0580    -0.1250    -0.0000 c3         1 MOL      -0.360411
      7 H5          -0.2780    -0.6730     0.8750 hc         1 MOL       0.100868
      8 H6          -0.4410     0.8380     0.0000 hc         1 MOL       0.100868
      9 H7          -0.2790    -0.6730    -0.8750 hc         1 MOL       0.100868
     10 C5           2.3950    -1.0780    -0.0000 ca         1 MOL      -0.241703
     11 H3           1.9620    -2.0650     0.0000 ha         1 MOL       0.155496
     12 C6           3.7700    -0.9440     0.0000 ca         1 MOL      -0.204480
     13 H4           4.3780    -1.8320    -0.0000 ha         1 MOL       0.143639
     14 C4           4.3740     0.3130    -0.0000 ca         1 MOL       0.001357
     15 C7           5.8880     0.4740     0.0000 c3         1 MOL       0.395136
     16 H8           6.0920     1.5420     0.0000 hc         1 MOL      -0.045496
     17 C10          6.5280    -0.1130    -1.2650 c3         1 MOL      -0.383188
     18 H9           6.3850    -1.1870    -1.3210 hc         1 MOL       0.088052
     19 H10          6.0980     0.3260    -2.1600 hc         1 MOL       0.088052
     20 H11          7.5970     0.0790    -1.2760 hc         1 MOL       0.088052
     21 C9           6.5280    -0.1120     1.2650 c3         1 MOL      -0.383188
     22 H12          6.0990     0.3260     2.1600 hc         1 MOL       0.088052
     23 H13          6.3850    -1.1870     1.3210 hc         1 MOL       0.088052
     24 H14          7.5970     0.0790     1.2760 hc         1 MOL       0.088052
@<TRIPOS>BOND
     1     2     1 1
     2     3     1 ar
     3     4     3 1
     4     5     3 ar
     5     6     5 1
     6     7     6 1
     7     8     6 1
     8     9     6 1
     9    10     5 ar
    10    11    10 1
    11    12    10 ar
    12    13    12 1
    13    14    12 ar
    14    15    14 1
    15    16    15 1
    16    17    15 1
    17    18    17 1

    18    19    17 1
    19    20    17 1
    20    21    15 1
    21    22    21 1
    22    23    21 1
    23    24    21 1
    24    14     1 ar
@<TRIPOS>SUBSTRUCTURE
     1 MOL         1 TEMP              0 ****  ****    0 ROOT
